# DNA Barcoding of German Cuckoo Wasps (Hymenoptera: Chrysididae) Suggests Cryptic Species in Several Widely Distributed Species

**DOI:** 10.3390/insects15110850

**Published:** 2024-10-30

**Authors:** Christian Schmid-Egger, Stefan Schmidt, Paolo Rosa, Oliver Niehuis

**Affiliations:** 1 Fischerstraße 1, 10317 Berlin, Germany; christian@bembix.de; 2 SNSB-Zoologische Staatssammlung München, 81247 Munich, Germany; 3 Laboratory of Zoology, Institute for Biosciences, University of Mons, Place du Parc 20, 7000 Mons, Belgium; 4 Department of Evolutionary Biology and Ecology, Institute of Biology I (Zoology), University of Freiburg, 79104 Freiburg, Germany; oliver.niehuis@biologie.uni-freiburg.de

**Keywords:** DNA barcoding, cytochrome oxidase I, COI, taxonomy, faunistics, hymenoptera, chrysididae

## Abstract

Germany is home to a rich cuckoo wasp fauna (Hymenoptera: Chrysididae) with about 108 species. However, several nomenclatural changes, the lack of identification keys, and the discovery of cryptic species difficult to identify based on external morphology have made the identification of several species a challenge. COI barcoding has been instrumental in the identification of some cuckoo wasp species and could help alleviate some of the above problems, but a reliable large reference database containing the cuckoo wasp barcodes is lacking. We present the COI barcodes of more than 800 specimens of 101 cuckoo wasp species native to Germany to lay the foundation for the barcode-based identification of German species. An analysis of the COI barcode sequences suggested groups that are largely consistent with the current taxonomy of the group. We found a few cases of over- or undersplitting of taxa. In some common species, the high degree of barcode divergence suggests the presence of cryptic species that need to be further assessed by integrative approaches. Our library of cuckoo wasp reference barcodes will enhance researchers’ ability to reliably identify species within this fascinating group of insects, in particular for identifying life stages that offer few or no morphological features for species-level identification.

## Simple Summary

Cuckoo wasps are aculeate hymenopterans, known for their striking metallic colour, which have earned them the nickname “jewel wasps”. In Europe, their larvae are parasitoids or cleptoparasites, targeting other hymenopterans such as sawflies, solitary wasps, and wild bees. With around 3000 species worldwide, 108 are native to Germany. Identifying many of these species based on external morphology is challenging due to the presence of cryptic species, lack of easily recognisable diagnostic features, and their complex taxonomy. While COI barcoding has proven helpful in species identification, its impact has been limited by the absence of a comprehensive reference database. In this study, we present COI barcodes for over 800 specimens, representing 101 cuckoo wasp species native to Germany. We identified potential cases of taxonomic oversplitting and observed barcode divergence in some widespread species, suggesting the presence of cryptic species that warrant further investigation. This barcode library will improve species identification, particularly in life stages that lack clear morphological features, making it a valuable resource for entomologists studying this diverse group of insects.

## 1. Introduction

**Figure 1:**
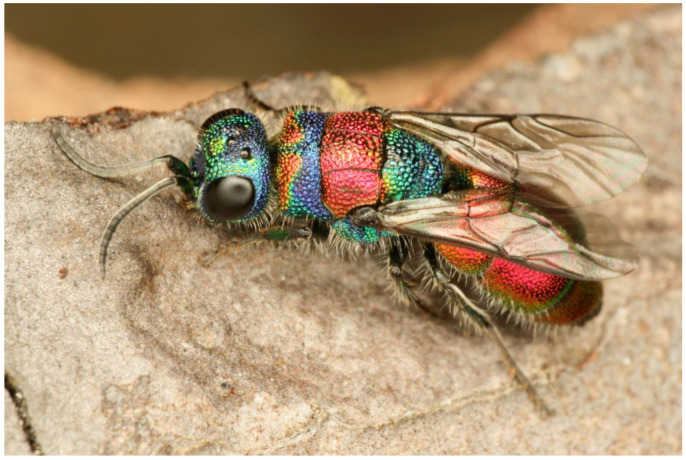
*Chrysis bicolor*, female. Photograph by T. Kwast.

Germany is home to a rich fauna of cuckoo wasps (Hymenoptera: Chrysididae), comprising at least 108 species in 18 currently recognised genera ([1,2,3]; present study). All the cuckoo wasp species recorded in the country are considered to be cleptoparasites (i.e., the cuckoo wasp larva feeds primarily on the prey of the host) or parasitoids (i.e., the cuckoo wasp larva feeds primarily on the host larva) of sawflies (Hymenoptera: Tenthredinidae) and aculeate Hymenoptera (i.e., Astatidae, Bembicidae, Crabronidae, Megachilidae, Pemphredonidae, Pompilidae, Psenidae, Sphecidae, and Vespidae; [4]). This fascinating aspect of their biology, combined with the spectacular iridescent colouration of many species [5], has made cuckoo wasps an attractive group to study (Figure 1). However, various nomenclatural changes in the last two decades (e.g., [3,6,7,8]), the lack of identification keys to all the currently accepted and recorded species in Germany, and the discovery of several cryptic species that are difficult to identify based on external morphological characters alone [9,10] have made the identification of a significant fraction of the fauna a challenge even for experts of this group.

DNA barcoding [11,12] has been instrumental in the identification of some cuckoo wasp species (especially in their male sex, e.g., *Chrysis ignita* species group), in the discovery of cryptic species, and in shedding light on the fascinating biology of some of the species in this family. For instance, the DNA barcoding of morphologically largely featureless larvae found in leafhoppers unexpectedly revealed that the cuckoo wasp *Elampus bidens* (Förster, 1853) does not oviposit directly into its host’s nest (as most other cuckoo wasps do), but rather into the host’s prey before the host hunts the prey to deposit it in the nest as food for its offspring [13]—a behaviour known as the “Trojan Horse strategy” in reference to the ruse used by the ancient Greeks to conquer the city of Troy [14]. Given the rarity of *E. bidens*, the chances of elucidating this fascinating aspect of the species’ biology from field observations alone would have been extremely low. Despite the clear advantages of DNA barcoding for the identification of well-defined species from samples of all the developmental stages and the discovery of cryptic species [11,12,15], a comprehensive reference library of cuckoo wasp DNA barcodes was lacking in Germany.

The existing DNA barcodes of cuckoo wasp species collected in Germany or collected elsewhere but known to occur also in Germany are scattered in the literature and are the result of research projects with very different foci (e.g., addressing specific taxonomic questions, and barcoding faunas outside Germany (e.g., [4,10,16,17,18,19,20])). Although these barcodes are very valuable, the species identity associated with some of the barcodes deposited in Barcode of Life Data Systems (BOLD, www.boldsystems.org (accessed on 24 September 2024)) and GenBank (https://www.ncbi.nlm.nih.gov/genbank/ (accessed on 24 September 2024)) needs to be treated with caution and verified. An example is the different DNA barcodes associated with the species *Pseudomalus violaceus* (Scopoli, 1763) in BOLD, one of which is clearly associated with a misidentified species. We, therefore, decided to create a reference library of cuckoo wasp DNA barcodes based on reliable species identifications.

The project started in 2009 as part of the “Barcoding Fauna Bavarica” (BFB) initiative [21,22] of the Zoologische Staatasammlung München, Germany. Consequently, the initial focus was on species occurring in Bavaria and its immediate surroundings. With the start of the “German Barcode of Life” (GBOL) project in 2012, the focus of the project was expanded to species occurring throughout Germany. The project also included the DNA barcoding of other groups of aculeate Hymenoptera, and the results of these efforts have already been published [23,24,25,26]. During the same period, the last author of this article independently compiled a library of cuckoo wasp barcodes to aid species identifications in projects related to the evolution of cuticular hydrocarbons (e.g., [10,27,28,29]). It made sense to combine the data of these independent efforts. As a result, we are able to present here the DNA barcodes of more than 800 cuckoo wasp specimens, including 101 of the 108 species reliably recorded from Germany.

## 2. Materials and Methods

Given the geographical focus of our study, most of the cuckoo wasp specimens used for barcoding were collected by us in Germany and are deposited in the collection of the Zoologische Staatsammlung München, or in the private collections of Oliver Niehuis (Freiburg i. Br.) or Christian Schmid-Egger (Berlin). Some samples from Austria, the Czech Republic, France, Hungary, Italy, Portugal, Slovakia, Spain, Sweden, and the Netherlands have been included when we were unable to obtain corresponding samples from Germany. Almost all of these samples were collected before October 2014. More recent samples were collected only in countries that have made their genetic resources freely available. Finally, we have included some previously published barcodes (obtained via download from NCBI) to supplement our dataset. Detailed information on the samples used for the BOLD TaxonID Tree (Supplementary Materials S1), like collection site, geographic coordinates, elevation, collector, identifier, voucher depository, and digital images of the sample, is provided in Supplementary Materials S2.

**Figure 2:**
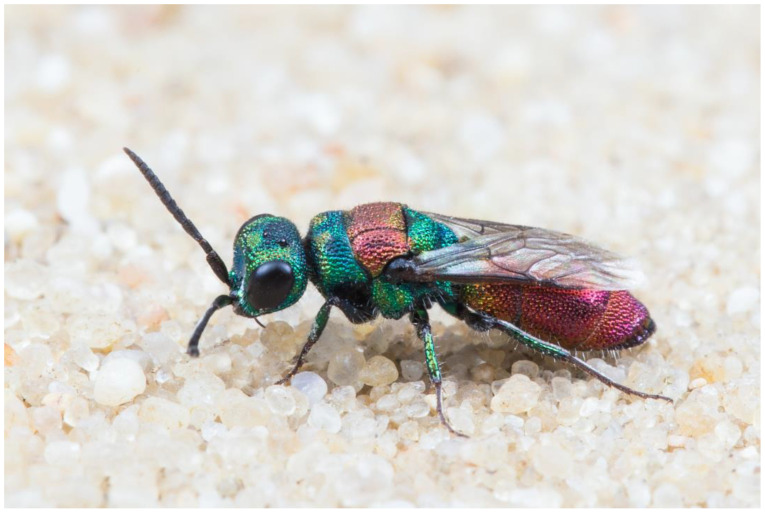
*Chrysis succincta*, female. Photograph by W.-H. Liebig.

**Figure 3:**
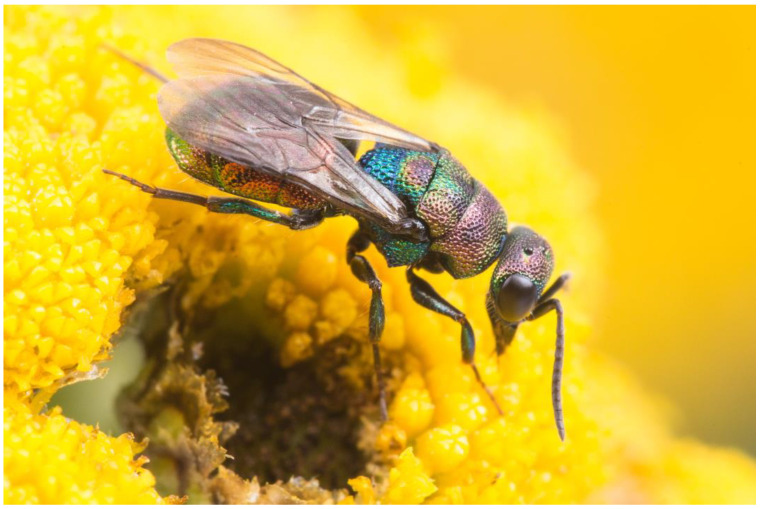
*Hedychrum rutilans*, female. Photograph by W.-H. Liebig.

**Figure 4:**
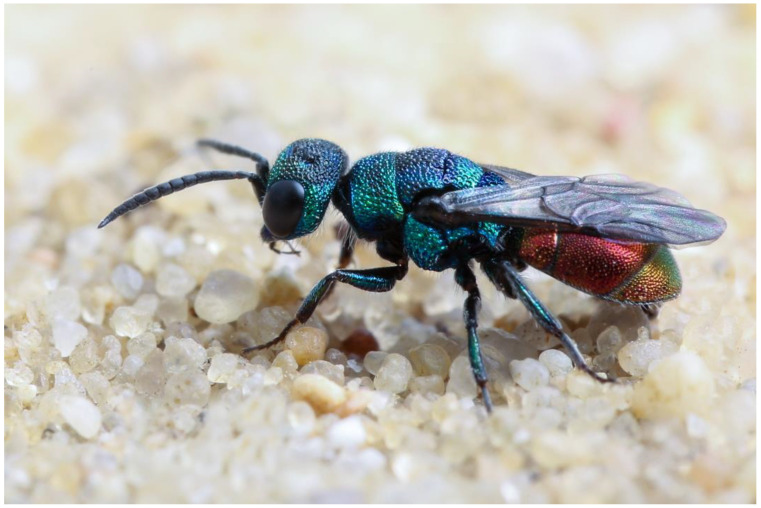
*Hedychridium rossicum*, male. Photograph by W.-H. Liebig.

**Figure 5:**
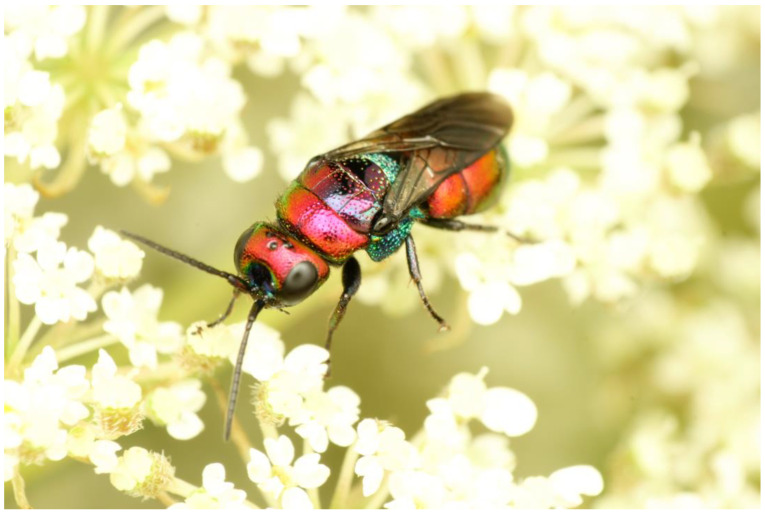
*Holopyga fervida*, male. Photograph by T. Kwast.

**Figure 6:**
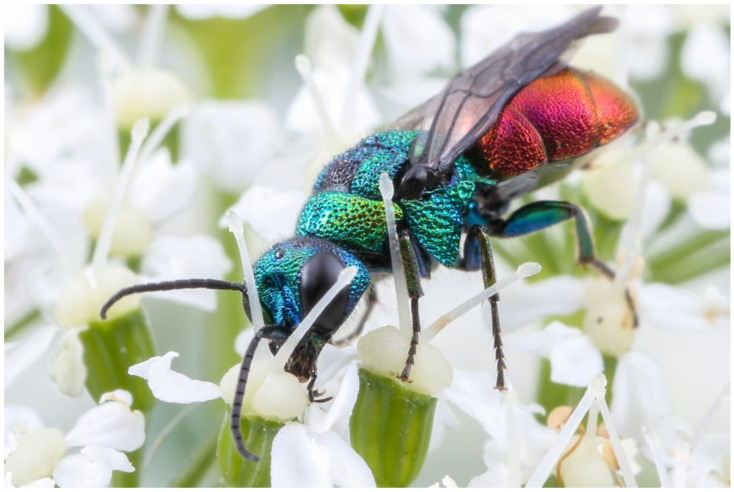
*Holopyga generosa*, male. Photograph by W.-H. Liebig.

Species considered and nomenclature: We have adopted the species list and nomenclature of German cuckoo wasps as presented by Wiesbauer et al. [3]. Additionally, we have included the recently described species *Chrysis parabrevitarsis* Soon et al. (2021) [10] and acknowledged the recent elevation of *Hedychrum rutilans* ssp. *viridiaureum* Tournier (1877) to species status, as proposed by Rosa et al. [18]. Furthermore, we have added *Chrysis lanceolata* Linsenmaier (1959) to our species list due to its confirmed occurrence in Germany, as indicated by our barcoding efforts. All the species currently recorded in Germany are included in this study, with the exception of seven species for which no barcodes could be obtained. Conversely, we included seven species in this project that have not yet been recorded in Germany, although some may potentially be found in the country. These are *Chrysis sculpturata* Mocsáry (1912), *Hedychridium aereolum* du Buysson (1893), *Hedychridium cupratum* (Dahlbom, 1854), *Hedychrum longicolle* Abeille de Perrin (1877), *Holopyga minima* Linsenmaier (1959), *Philoctetes putoni* (du Buysson, 1892), and *Stilbum calens* (Fabricius, 1781). However, these species were not subjected to further analysis. We illustrate six cuckoo wasp species from Germany whose COI barcodes have revealed new taxonomic insights (Figure 1, Figure 2, Figure 3, Figure 4, Figure 5 and Figure 6).

Molecular procedures: Samples collected as part of BFB and GBOL were processed at the Canadian Centre for DNA Barcoding (https://ccdb.ca (accessed on 24 September 2024)) in Guelph, Canada. The facility applied the DNA extraction, COI DNA sequence amplification, and DNA sequencing protocol outlined by Ivanova et al. (2006). The samples processed in the molecular laboratory of the last author followed the protocol of Pauli et al. [30]. 

Data analysis: The COI nucleotide sequences were analysed using the software tools provided by BOLD. The divergence between nucleotide sequences was calculated using the Kimura two-parameter DNA substitution model [31]. The calculated distance values were used to infer a neighbour-joining tree, with the labels of the terminal taxa informing about species identification, sample ID, sex, country, province/state, region, sequence length (bp with the number of ambiguous bases in square brackets), and the Barcode Index Number (BIN) assigned by BOLD. BINs represent COI nucleotide sequence clusters that correlate with biological species entities [32]. Please note that we present the alphanumeric Barcode Index Numbers (BINs) without the “BOLD”: prefix, as it is redundant. However, to search for specific BINs in the BOLD system, the prefix must be added.

## 3. Results

For the present study, the DNA barcode sequences of 806 specimens of cuckoo wasp were analysed (Supplementary Materials S1). All the barcodes are more than 500 nucleotides long and contain less than 1% ambiguous nucleotides and were used for further analyses. The sequences of the complete dataset represent 108 species, including the barcodes of 101 species considered native to Germany, thus representing 94% of the German Chrysididae fauna. The seven additional species are from outside Germany (see Material and Methods). The sequences and distances are presented as a neighbour-joining tree in Supplementary Materials S1, with BIN assignments indicated by different branch colours. Additionally, a list of voucher specimens is provided in Supplementary Materials S2, which includes specimen ID, country of origin, collection date, specimen depository, Barcode Index Number (BIN), and sequencing success. The barcoding statistics, including mean intraspecific distance, maximum intraspecific distance, nearest neighbour species, distance to nearest neighbour species, Barcode Index Number (BIN), country, and number of specimens, are presented in Supplementary Materials S3.

## 4. Discussion

The majority of our barcode clusters are consistent with the current taxonomy of the family (i.e., BINs correspond to accepted species). However, we also uncovered a number of cases in which the barcode sequences differ surprisingly little between bona fide species, or in which the barcode sequences within the currently accepted species differ to a degree often seen between bona fide species. We discuss each of these cases in detail below.

- ***Chrysis bicolor* Lepeletier, 1806 (Figure 1)**

Our barcoding efforts of the samples from Southern Germany, the French Alps, and the Italian Alps revealed a remarkable intraspecific divergence of up to 3.4% and the assignment of our samples to a total of three BINs (AAY6947, AEC7638, and AAY6926) corresponding to the country of origin of the samples. Whether the three BINs represent separate species remains to be investigated and is beyond the scope of the present study. However, if future studies show that the populations of *C. bicolor* from the French Alps and from Germany should be treated as separate species, the name *C. bicolor* would likely refer to BIN AAY6926 from the French Alps, as the neotype of *C. bicolor* designated by Rosa and Xu [33] originates from Saint-Laurent-du-Verdon (Dept. Alpes-de-Haute-Provence, France).

- ***Chrysis leachii* Shuckard, 1837**

Haris [34] synonymized *Chrysis leachii* Shuckard, 1837 with *C. hungarica* Scopoli, 1772, a decision contrary to the opinions of some experts (e.g., PR) on the specific identity of *C. hungarica* (based on its short description) and contrary to the provisions of the ICZN [35]. In such a case, the reversal of precedence should have been applied, as Articles 23.9.1.1 and 23.9.1.2 of the ICZN are satisfied. The name *Chrysis hungarica* had not been used as a valid taxon name after 1899 (effectively since 1772) and should have been considered as a *nomen oblitum*. The putative junior synonym, *Chrysis leachii*, should have been considered a *nomen protectum* because it had been used as a valid name in at least 25 articles published by at least ten authors in the immediately preceding 50 years, covering a period of at least 10 years. Furthermore, Haris [34] synonymized *C. leachii* without designating neotypes for *C. hungarica* and *C. leachii*, whose types are both considered lost according to Backer [36] and Haris [34]. A neotype designation would be necessary because Scopoli’s extremely brief description (only three lines of text) of *C*. *hungarica* could refer to several currently accepted species that could have occurred at the type locality. As a consequence, the following authors deliberately disregarded the taxonomic change suggested by Haris [34] and instead considered the synonymy proposed by Mocsary [37], which included *C*. *hungarica* in the synonymy list of *C. succincta* Linnaeus, 1767, to be valid—the authors of the recent molecular phylogenies of the family Chrysididae [4,30], of books on Chrysididae [3,38], and of various taxonomic and faunistic articles (e.g., [39,40,41,42,43,44,45,46,47]). The cited articles have more than 30 authors and include some of the leading experts on the family.

Given the ongoing debate about the validity of the synonymy of *C*. *hungarica* and the lack of a designated neotype of this taxon, efforts are needed to achieve long-term taxonomic stability. To this end, we decided to designate a neotype of *C. hungarica* consistent with Mocsary’s [37] interpretation of this taxon as a synonym of *Chrysis succincta* Linnaeus, 1767. The identity of *C. succincta* was previously secured in the long term by Rosa and Xu [33], who designated a neotype of this taxon in the Linsenmaier collection (Luzern, CH). To provide a certain synonymy with *Chrysis succincta*, we select as the neotype of *C. hungarica* the neotype of *C. succincta* collected at Bromberg [= Bydgoszcz, Kuyavian-Pomeranian Voivodeship] in Poland on 24 May 1920, by Meyer and deposited in the Linsenmaier collection.

The type locality of *Chrysis hungarica* is Kremnica, formerly situated in the Kingdom of Hungary and now located in Slovakia (Banskobystrický region). Although no material is currently available to us from this area, *C. succincta*, *C. leachii*, and related species, such as *C. lanceolata* Linsenmaier, 1959, have been recorded from Slovakia. The brief description of *C. hungarica* provided by Scopoli 1772 is furthermore fully consistent with this taxon being considered a synonym of *C. succincta*, as first suggested by Mocsary [37]: “*Viridi colore intent caput, thorax, abdominis basis, et margine segmentorum aliarum. Fascia thoracis, et segmenta duo abdominis postica ignito-aurata*” and “*Parva subtus viridis nitens; abdomine subtus concavo fusco-aurato; antennis pedibusque nigris; abdomine minime dentato*”. While the description of the third tergite (i.e., “*abdomine minime dentato*” = “abdomen minimally toothed”) corresponds to both *C. leachii* and *C. succincta*, the description of the metasomal colouration (i.e., “*Fascia thoracis* […] *ignito-aurata” =* “thoracic red fiery band”) corresponds more closely to *C. succincta* than to *C. leachii.* Note that Scopoli did not specify the sex of the specimen/-s he described as *C. hungarica.* Although Mocsáry [37] suggested that the specimen/-s was/were female/-s, the neotype we designate is a male.

- ***Chrysis succincta* Linnaeus, 1767 (Figure 2)**

Rosa and Xu [33] designated a neotype of *Chrysis succincta* collected in Poland and proposed to treat populations of this species from the Alps of Switzerland, Italy, and France as a separate species, which they proposed to name *C. tristicula* Linsenmaier, 1959. While *Chrysis succincta* is locally common in eastern Germany, it has also been historically recorded in southern Germany [1] and thus in relatively close spatial proximity to *C. tristicula*. We, therefore, considered individuals from Saxony and from the French Alps (Millefonts, Dept. Alpes-Maritimes, France; KY430731) in our sampling. We found that the barcodes of the two geographically separated populations are virtually identical (0.2% divergence) and to be assigned to the same BIN (i.e., AEC9270). We, therefore, consider the Alpine populations of *C. tristicula* (previously also known as *C. succincta succinctula sensu* Linsenmaier [48]) to be conspecific with *C. succincta*, as they were prior to the publication of Rosa and Xu [33]. The taxonomic status of *C. tristicula* populations occurring on the Iberian Peninsula and in Morocco remains unaffected by our interpretation and requires further investigation in future studies.

- ***Chrysis ignita* species group**

The *Chrysis ignita* species group represents one of the most taxonomically challenging cuckoo wasp lineages in Europe. Uncertainties in the taxonomic status of different phenotypes have made it the focus of several molecular phylogenetic and taxonomic studies [9,10,16,20,49,50]. Van der Smissen [51] provided a key to the German species of the group. However, the identification of male specimens has remained a major challenge, and the recent descriptions of various cryptic species have further complicated matters.

We were able to barcode 24 of the 27 German species of this group. The three missing species are *Chrysis mediadentata* Linsenmaier, 1951; *C. obtusidens Dufour & Perris*, 1840; and *C. valida* Mocsáry, 1912. We found that the barcodes of the most currently accepted species in the *ignita* species group are assigned to separate BINs. However, we found a few cases of (presumed) oversplitting or lumping which we discuss.

- ***Chrysis mediata* Linsenmaier, 1951 and *Chrysis solida* Haupt, 1956**

*Chrysis mediata* and *C. solida* are morphologically very similar. Confidence in treating them as separate species comes primarily from their use on different hosts: *C. mediata* is a cleptoparasite of the soil-nesting vespid wasps of the genus *Odynerus* Latreille, 1802, whereas *C. solida* is a cleptoparasite of the cavity-nesting vespid wasps primarily of the genus *Ancistrocerus* Wesmael, 1836 and *Euodynerus* Rossi, 1790 [4,9]. The two species also show subtle morphological and chromatic differences: adult *C. mediata* typically has a larger and proportionally broader body than *C. solida*; in frontal view, the eyes of *C. solida* appear proportionally larger than those of *C. mediata*, which in turn makes the cavitas frontalis appear narrower in *C. solida* than in *C. mediata*. Finally, the colouration of the head and mesosoma is typically darker blue in *C. solida* than in *C. mediata*. The latter may also be greenish [49].

Our barcodes of *Chrysis mediata* and *C. solida* clustered into a single BIN (i.e., AAY6949). The visual inspection of the aligned barcode sequences at the nucleotide level revealed a single site where the two species appear to differ consistently. Recently, Dietz et al. [16] examined the same species pair using a nuclear set of barcoding markers and found reliable differences between the two taxa at the nuclear genomic level.

- ***Chrysis ignita* (Linnaeus, 1758) and *Chrysis impressa* Schenck, 1856**

Distinguishing samples of *Chrysis ignita* and *C. impressa* can be difficult, especially if they are males, but the validity of the two species has not been disputed recently. We found that our samples of the two species were, nonetheless, clustered into a single BIN (i.e., AAG0244). The visual inspection of the aligned barcode sequences at the nucleotide level revealed four sites where the two species appear to differ consistently. These differences are reflected in the inferred neighbour-joining tree, in which the two species form separate clades. Dietz et al. [16] also examined this species pair and found reliable differences between the two species at the nuclear genomic level.

- ***Chrysis parietis* Budrys, 2016 and *Chrysis schencki* Linsenmaier, 1968**

*Chrysis parietis* has been described comparatively recently, mainly due to its COI barcode sequences, which differ from those of the other species in the *C. ignita* species group [9]. We found that the COI barcodes of this species are clustered in a single and species-specific BIN, AAU2329. In the neighbour-joining tree, *C. parietis* clusters next to one of the two clades of *C. schencki*. The barcodes of the two *C. schencki* clades differ by up to 2.4% and were assigned to two different BINs (i.e., ABU6375 and ACF6219). Since *C. schencki* is morphologically and chromatically quite variable, the barcode differences could theoretically be due to cryptic species diversity. Dietz et al. [16] included samples from the two *C. schencki* clades in a study evaluating the advantages of a nuclear barcoding approach. They found that the mitochondrial barcode divergence in *C. schencki* does not correspond to nuclear barcode divergence, suggesting that samples with different COI barcodes do not represent reproductively isolated entities. Given this finding, it would be desirable to verify the taxonomic status of *C. parietis* in a similar manner.

- ***Hedychrum viridiaureum* Tournier, 1877**

*Hedychrum viridiaureum* was previously treated as a subspecies of *H. rutilans* Dahlbom, 1854 (Figure 3), until Rosa et al. [18] elevated it to species rank. The authors based their decision on the comparatively large divergence (5.3%) of the COI barcode sequences, which formed two BINs, each containing nucleotide sequences that differ very little from each other. Based on the distribution of the two barcode BINs, they assigned the BIN containing barcodes sampled primarily in southwestern Europe to *H. viridiaureum* and the BIN containing barcodes sampled primarily in eastern and northern Europe to *H. rutilans*. The authors also list the morphological differences and discuss the possible host use differences between the two species.

The barcodes of *Hedychrum rutilans* in the broad sense (including *H. viridiaureum*) from the wasps collected at different locations in Germany revealed the two BINs reported by Rosa et al. [18]. BIN AAM3491 was found exclusively in all the wasps collected in south-western Germany. BIN AAK4643 was found exclusively in all the wasps collected in eastern Germany, suggesting that the distribution of *H. rutilans* in the narrow sense is much more restricted than previously thought. However, we are not aware of any wasps collected in southern Germany that exhibit the morphological features considered characteristic of *H. viridiaureum* by Rosa et al. [18].

Differences in host use by wasps with different barcodes seem highly unlikely in Germany: *Hedychrum rutilans* in the broad sense is known to be a brood parasite of the digger wasps of the genus *Philanthus* Fabricius, 1790 (Aculeata: Philanthidae), of which only two species occur in Germany. One of them, *Philanthus triangulum* (Fabricius, 1775), is widespread and can be found all over the country [52]. The other, *Philanthus coronatus* (Thunberg, 1784), is very rare, occurring only in a few locations in southern Germany [53]. The distribution of the two species that are known (*P. triangulum*) or could potentially be used (*P. coronatus*) as hosts in Germany is inconsistent with the idea that the wasps of each barcode group are specialised on a different host.

While we found a widespread distribution of the *Hedychrum viridiaureum* COI barcode in southwestern Germany, we lack evidence for the occurrence of nuclear genetic information for this taxon, regardless of whether this taxon is considered a species or a subspecies. The homogeneous appearance of *H. rutilans* in Germany suggests a single taxon in the country. The presence of the *H. viridiaureum* COI barcode could be due to incomplete lineage sorting, introgression, or a lack of reproductive isolation between the western and eastern populations of *H. rutilans* in the broader sense. This issue, therefore, requires further investigation. Until then, we conservatively do not consider *H. (rutilans) viridiaureum* as a taxon occurring in Germany. Note that we chose to retain the taxon name *H. viridiaureum* in the COI phylogram to facilitate the visual association of the samples from Germany with this specific barcode.

- ***Hedychridium roseum* (Rossi, 1790)**

We found that the barcodes of *Hedychridium roseum* from Germany are distinct from those of *H. rossicum* Gussakowskij, 1848 (see below), but cluster into two BINs (AAE3259 and ACG6690). The maximum distance between the samples identified as *H. roseum* is 1.9%. While BIN ACG6690 is monophyletic, BIN AAE3259 is paraphyletic. We are currently unaware of any evidence for the presence of cryptic species in this taxon within Germany. A nuclear barcoding approach, such as the one proposed by Eberle et al. [15] and applied by Dietz et al. [16], could provide clues as to whether or not the above-mentioned comparatively small barcode divergence may be related to reproductive isolation.

- ***Hedychridium rossicum* Gussakowskij, 1948**

The taxonomic status of this taxon has been controversial in the past. *Hedychridium rossicum* (= *H. valesiense* Linsenmaier, 1959; [7]) was thought to be a species in which both sexes have an iridescent metasoma, in contrast to *H. roseum*, in which both sexes are thought to have a non-iridescent metasoma. Niehuis [54] noted that the large number of *H. rossicum* specimens in private and museum collections are all males. To assess whether *H. rossicum* is a colour morph of *H. roseum* or whether *H. rossicum* is a valid species whose females have been misidentified as *H. roseum*, he barcoded males with and without iridescent metasoma at sites in Germany and Italy where they occur in sympatry and found that samples from the same site had the same barcode and samples from different sites had different barcodes. The most parsimonious explanation for this observation was that *H. rossicum* is a colour morph of *H. roseum*. Niehuis [1,54], therefore, suggested that *H. rossicum* should be considered conspecific with *H. roseum*. Arens [55] reported morphological discontinuities in the shape of the metapleura of samples of *H. roseum* in the wide sense (including. *H. rossicum*) and argued that these could be used to distinguish the females of *H. roseum* from the females of *H. rossicum*. Unaware of the above barcoding results, he assumed that an iridescent metasoma in the male sex was indicative of *H. rossicum* and a non-iridescent metasoma in the male sex was indicative of *H. roseum*. The barcoding of additional samples in conjunction with the analysis of their cuticular hydrocarbons by O. Niehuis, R. F. Castillo-Cajas, and T. Schmidt (unpublished data) shows that *H. rossicum* and *H. roseum* represent two valid species. However, the widespread assumption that an iridescent metasoma in the male sex is indicative of *H. rossicum* and a non-iridescent metasoma in the male sex is indicative of *H. roseum* (e.g., [55,56]) proved to be incorrect. While the males *of H. roseum* apparently never have an iridescent metasoma, the males of *H. rossicum* can express two different phenotypes: one with an iridescent metasoma and one with a non-iridescent metasoma. As a consequence, the reported records of male *H. roseum* must be considered unreliable, as they could refer to *H. rossicum*.

- ***Holopyga chrysonota* (Förster, 1853)**

We found that the barcodes of *Holopyga chrysonota* differ up to 5.1% from each other and were assigned to two different BINs. One of them (AAY9689) contains samples from Rhineland-Palatinate (Germany) and Valle d’Aosta (Italy), and the other one (AAV7063) contains a single sample from Saxony-Anhalt (Germany). We are currently unaware of any evidence for the presence of a cryptic species in this taxon within Germany. A nuclear barcoding approach, such as the one proposed by Eberle et al. [15] and applied by Dietz et al. [16], could provide clues as to whether or not the barcode divergence may be related to reproductive isolation. Note that until recently, this species was referred to as *Holopyga ignicollis* Dahlbom, 1854 in the literature and that the species previously referred to as *H. chrysonota* is now referred to as *Holopyga similis* Mocsáry, 1889 [57].

- ***Holopyga fervida* (Fabricius, 1781) (Figure 5)**

We found that the barcodes of *Holopyga fervida* differ by up to 7.3%, and they were assigned to three distinct BINs. One BIN (AAX1104) contains a single specimen from Brandenburg, the second (ACV6331) includes two specimens from Baden-Württemberg (Germany), and the third (AAY9735) contains a single specimen from Hesse (Germany) along with samples from the Balearic Islands (Spain). Whether this high level of divergence is due to the presence of a cryptic species among *H. fervida* cannot be answered at this time without additional data (e.g., nuclear barcodes; [15]). Given the great difficulty in distinguishing some bona fide species in this group, at least in the female sex, we consider the presence of an overlooked species quite possible.

- ***Holopyga generosa* (Förster 1853) (Figure 6)**

The barcodes of *Holopyga generosa* are assigned to a total of four BINs (i.e., AAY6927, AAY6928, AAZ6194, and ACC3318). The wasps from three of the four BINs (AAY6928, AAZ6194, and ACC3318) were collected in Germany; the wasps from the fourth BIN were collected exclusively in the French Alps (AAY6927). The barcode divergence between the samples currently assigned to *H. generosa* is remarkably high, reaching more than 13% in some comparisons (i.e., between the wasps from BIN AAY6927 and BIN AAY6928). This high level of divergence strongly suggests the presence of cryptic species. The barcodes presented here will hopefully stimulate future taxonomic studies that assess the presence of cryptic species in this taxon using an integrative approach (e.g., considering data from morphology, nuclear barcoding, and chemical ecology).

- ***Omalus aeneus* (Fabricius, 1787) and *Omalus puncticollis* Mocsáry, 1887**

We were able to study the barcodes of only four specimens of this species group, collected from distant locations (i.e., Germany, Hungary, Italy, and Slovakia). Despite the small sample size, our barcode results highlight two interesting aspects: (1) A sample with all the morphological characteristics of *Omalus aeneus* carries a barcode that is virtually (99.9%) identical to the barcode found in a sample with all the morphological characteristics of *O. puncticollis*; the two samples were also, not surprisingly, assigned to the same BIN (i.e., ACC4462). (2) The three samples in our dataset that are identified as *O. aeneus* differ between 7.0% and 9.1% from each other and were assigned to three different BINs (i.e., ACC4462, ACG9650, and ACQ9469). Such a remarkably high level of divergence was previously reported by Paukkunen et al. [49] in *O. aeneus* samples from Fennoscandia and the Baltic States. It may indicate the presence of cryptic species among samples identified as *O. aeneus*.

Given the degree of variation in the punctuation on the pro- and mesonotum, which is primarily used to separate *O. aeneus* and *O. puncticollis*, Niehuis [1] questioned the taxonomic validity of *O. puncticollis.* At first glance, our barcoding results seem to be consistent with this interpretation. However, using an integrative approach, F. Brunßen and the last author in an unpublished study found a 1:1 association between cuticular hydrocarbon profiles and different barcodes in samples of *O. aeneus* and *O. puncticollis* collected in sympatry or close spatial proximity to each other. Furthermore, the first results from USCO gene barcoding [15] of the same samples (L. Podsiadlowski, O. Niehuis, unpublished study) provided additional evidence for the presence of cryptic species. They also suggest that the punctation on the pro- and mesonotum alone may not be sufficient to reliably separate *O. puncticollis* from some species currently grouped under the name *O. aeneus*.

## 5. Conclusions

Wiesbauer et al. [3] mention Germany in the distribution of 112 Central European cuckoo wasp species. However, one of the listed species (*Hedychridium mosadunense* Lefeber, 1986) is a synonym of *H. femoratum* [58]. Another species, *Chrysura laevigata* (Abeille de Perrin, 1879), was listed on the basis of a specimen identified as this species by W. Linsenmaier (1 female, Würzburg, 12 July 1953, leg. Dr. Enslin, det. ‘59 W. Linsenmaier; Linsemaier collection in Luzern/CH), but a recent re-examination by O. Niehuis and P. Rosa showed it to be *Chrysura dichroa*. A third species, *Hedychridium jucundum* (Mocsáry, 1889), is erroneously listed due to the confusion of locality names. The regular occurrence of two other species seems highly questionable: *Chrysellampus sculpticollis* (Abeille de Perrin, 1878) (single record far away from the nearest known occurrences of this species [59]; *Cleptes aerosus* Förster, 1853 (single record far away from the next known regular sites of this species [60]). The German records of *Hedychrum viridiaureum* Tournier, 1877 are not accepted due to the present examination. At the same time, *Chrysis ragusae* De Stefani, 1888, whose status in Germany was questioned by Wiesbauer et al. [3], has now been recorded several times (L. Bertsch, in lit.; vid. O. Niehuis), confirming that the species is established in the country. A re-examination of the samples identified as *Holopyga inflammata* (Förster, 1853) from Bavaria in the Linsenmaier collection by O. Niehuis and P. Rosa also confirmed the former presence of this species in Germany (1 male, Würzburg, 1 June 1920, leg. Dr. Enslin; 1 male Fränkischer Jura, 21 July 1934, leg. Dr. Enslin). Together with *C. parabrevitarsis*, a species recently described from specimens collected in Germany [10], and *C. lanceolata*, reported for the first time from Germany in this study, the total number of species considered native to Germany is 108. Of these species, we were able to examine the barcodes of 94% either by de novo sequencing them or by considering published barcodes that we consider reliable. The only seven species whose barcodes we did not study are *Chrysis calimorpha* Mocsáry, 1882, *C. mediadentata*, *C. obtusidens*, *C. ragusae*, *C. valida*, and *Cleptes britannicorum* Rosa, 2024. At the same time, our barcode database contains the barcodes of two species mentioned by Wiesbauer et al. [3] in the context of Germany (see above), but which we do not currently consider native (i.e., *C. sculpticollis* and *H. jucundum*).

The proportion of barcoded cuckoo wasp species has reached approximately the same level as that in the studies of other groups of aculeate Hymenoptera [23,24,25,26]. More importantly, we believe that the level of completeness is sufficient for all practical purposes (i.e., the identification of wasps collected in Germany and guiding future taxonomic research on cuckoo wasps). We expect the barcodes of at least five, if not all, of the missing species to be sufficiently distinct that they will not be clustered in the same BIN with other species. Thus, the barcodes of the seven missing species should stand out as unique.

The alpha taxonomy of cuckoo wasps occurring in Germany, in contrast to most other groups of aculeate Hymenoptera, has been quite extensively studied by DNA sequencing [9,15,18,20,49,50,54]. Therefore, we were surprised by the number of species that we were still able to find with barcode diversity that could be indicative of cryptic species. While the presence of cryptic species in the *O. aeneus* species group was already suspected and the group is, therefore, the focus of an integrative taxonomic study (F. Brunßen, O. Niehuis, in lit.), the diversity found in the other taxa (i.e., *H. roseum*, *H. chrysonota*, *H. fervida*, and *H. generosa*) still awaits integrative taxonomic treatment.

We hope that our library of cuckoo wasp reference barcodes will help researchers to more reliably identify species of this interesting group of insects, especially in life stages (e.g., eggs and larvae) that provide little or no morphological characters for species-level identification, and to stimulate integrative taxonomic research on the few remaining problematic taxa. The discovery of *C. lanceolata* in Germany by the DNA barcoding of a specimen previously thought to be a female of *C. cortii* with aberrant colouration (Rhineland-Palatinate, Monsheim, 15 July 2006, leg. G. Reder) is a testament to the usefulness of DNA barcoding. However, our barcoding results from *H. viridiaureum* also remind us that a single marker from the non-recombining mitochondrion alone can be misleading.

We view barcode differences as indications of the possible presence of cryptic species (= unconfirmed candidate species sensu Padial et al. [61]), which should then be evaluated using an integrative approach. This reluctance may sound unsatisfactory to some, who may feel left with the uncertainty of whether or not a taxon actually represents one or more species. However, the specific characteristics of the COI barcode marker alone cannot provide a clear answer. The marker is most powerful when interpreted in combination with nuclear-encoded characters, such as nuclear barcodes, morphology, or cuticular hydrocarbon profiles. Before assessing COI barcode diversity in the problematic taxa mentioned above, the most transparent strategy to deal with the few remaining taxonomic uncertainties is to report the corresponding species with their contained barcode, if this information is available.

## Data Availability

Barcode data can be obtained from BOLD, if desired as a single citable dataset (dx.doi.org/10.5883/DS-CHRYSEUR), and include PCR oligonucleotide primer information and access to DNA trace files.

## Supplementary Materials

The following supporting information can be downloaded at https://www.mdpi.com/article/10.3390/insects15110850/s1, Supplementary Material S1: PDF file. BOLD TaxonID Tree. Supplementary Material S2: List of voucher specimens with specimen ID, country of origin, collection date, specimen depository, Barcode Index Number (BIN), and sequencing success (COI-fragment length in bp, in square brackets number of unresolved bases). Supplementary Material S3: Barcoding statistics, including the mean intraspecific distance, maximum intraspecific distance, nearest neighbour species, distance to the nearest neighbour species, Barcode Index Number (BIN), country, and number of specimens. Asterisks indicate species that share a BIN.
