# Supplementary material for: DNA Barcoding of German Cuckoo Wasps (Hymenoptera: Chrysididae) Suggests Cryptic Species in Several Widely Distributed Species
Source: Insects. 2024 Oct 30;15(11):850. doi: 10.3390/insects15110850 (PMC11594385; doi:10.3390/insects15110850)
Supplement: Supplementary file 1 [file insects-15-00850-s001.zip › Supplementary materials/Supplement 1 Chrysididae tree.pdf]

BOLD TaxonID Tree

Title	:	Tree Result - DS-CHRYSEUR
Date	:	31-Aug-2024
Data Type	:	Nucleotide
Distance Model	:	Kimura 2 Parameter
Marker	:	COI-5P
Colourization	:	Barcode Cluster (BIN)

Label	:	Sample ID
Label	:	Taxon
Label	:	Country
Label	:	Province/State
Label	:	Barcode Cluster (BIN)

Sequence Count	:	804
Species count	:	113
Genus count	:	19
Family count	:	1
Unidentified	:	0

BIN Count	:	125

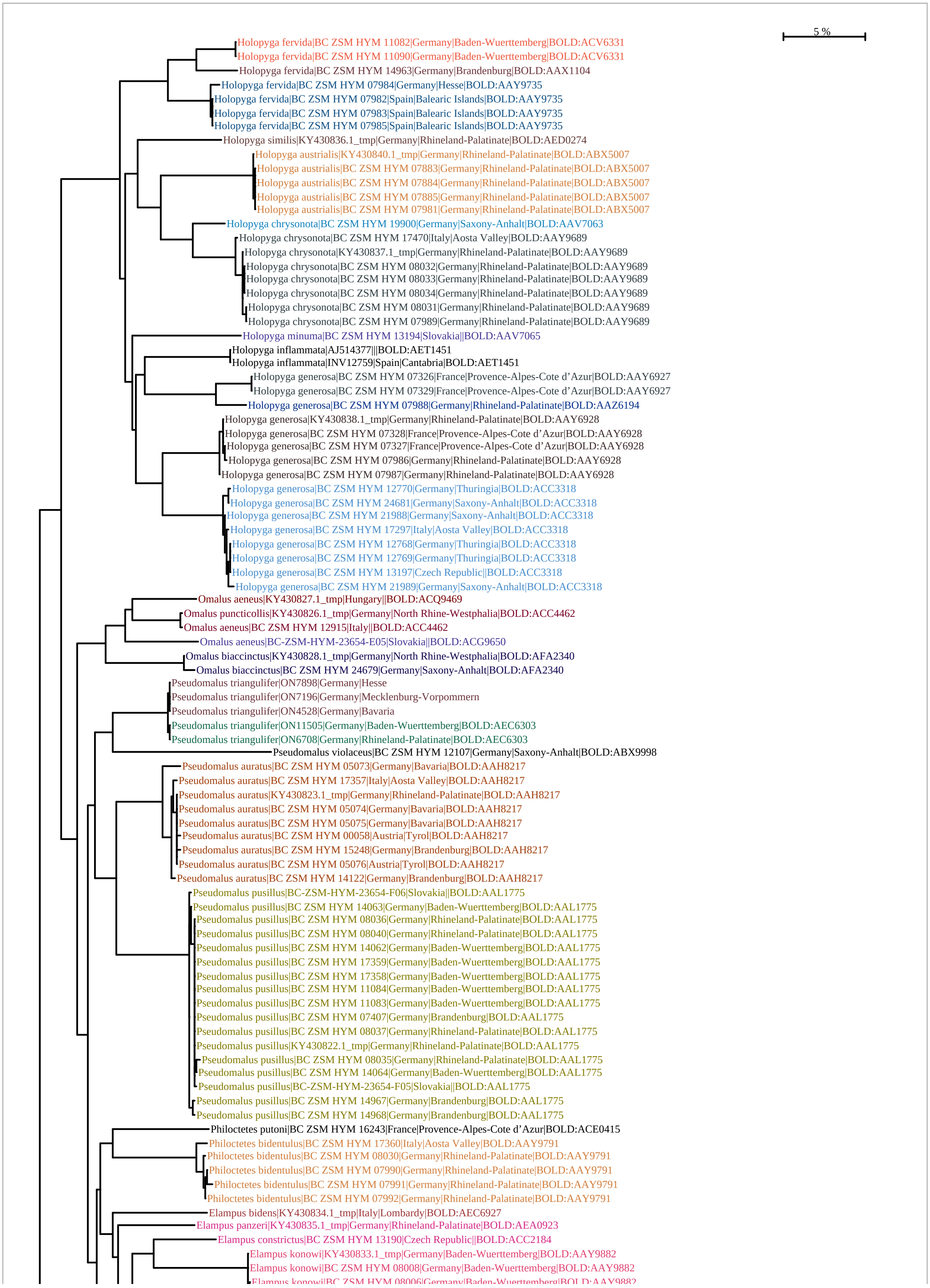
5 %
Holopyga fervida|BC ZSM HYM 11082|Germany|Baden-Wuerttemberg|BOLD:ACV6331
Holopyga fervida|BC ZSM HYM 11090|Germany|Baden-Wuerttemberg|BOLD:ACV6331
Holopyga fervida|BC ZSM HYM 14963|Germany|Brandenburg|BOLD:AAX1104
Holopyga fervida|BC ZSM HYM 07984|Germany|Hesse|BOLD:AAY9735
Holopyga fervida|BC ZSM HYM 07982|Spain|Balearic Islands|BOLD:AAY9735
Holopyga fervida|BC ZSM HYM 07983|Spain|Balearic Islands|BOLD:AAY9735
Holopyga fervida|BC ZSM HYM 07985|Spain|Balearic Islands|BOLD:AAY9735
Holopyga similis|KY430836.1_tmp|Germany|Rhineland-Palatinate|BOLD:AED0274
Holopyga austrialis|KY430840.1_tmp|Germany|Rhineland-Palatinate|BOLD:ABX5007
Holopyga austrialis|BC ZSM HYM 07883|Germany|Rhineland-Palatinate|BOLD:ABX5007
Holopyga austrialis|BC ZSM HYM 07884|Germany|Rhineland-Palatinate|BOLD:ABX5007
Holopyga austrialis|BC ZSM HYM 07885|Germany|Rhineland-Palatinate|BOLD:ABX5007
Holopyga austrialis|BC ZSM HYM 07981|Germany|Rhineland-Palatinate|BOLD:ABX5007
Holopyga chrysonota|BC ZSM HYM 19900|Germany|Saxony-Anhalt|BOLD:AAV7063
Holopyga chrysonota|BC ZSM HYM 17470|Italy|Aosta Valley|BOLD:AAY9689
Holopyga chrysonota|KY430837.1_tmp|Germany|Rhineland-Palatinate|BOLD:AAY9689
Holopyga chrysonota|BC ZSM HYM 08032|Germany|Rhineland-Palatinate|BOLD:AAY9689
Holopyga chrysonota|BC ZSM HYM 08033|Germany|Rhineland-Palatinate|BOLD:AAY9689
Holopyga chrysonota|BC ZSM HYM 08034|Germany|Rhineland-Palatinate|BOLD:AAY9689
Holopyga chrysonota|BC ZSM HYM 08031|Germany|Rhineland-Palatinate|BOLD:AAY9689
Holopyga chrysonota|BC ZSM HYM 07989|Germany|Rhineland-Palatinate|BOLD:AAY9689
Holopyga minuma|BC ZSM HYM 13194|Slovakia||BOLD:AAV7065
Holopyga inflammata|AJ514377|||BOLD:AET1451
Holopyga inflammata|INV12759|Spain|Cantabria|BOLD:AET1451
Holopyga generosa|BC ZSM HYM 07326|France|Provence-Alpes-Cote d'Azur|BOLD:AAY6927
Holopyga generosa|BC ZSM HYM 07329|France|Provence-Alpes-Cote d'Azur|BOLD:AAY6927
Holopyga generosa|BC ZSM HYM 07988|Germany|Rhineland-Palatinate|BOLD:AAZ6194
Holopyga generosa|KY430838.1_tmp|Germany|Rhineland-Palatinate|BOLD:AAY6928
Holopyga generosa|BC ZSM HYM 07328|France|Provence-Alpes-Cote d'Azur|BOLD:AAY6928
Holopyga generosa|BC ZSM HYM 07327|France|Provence-Alpes-Cote d'Azur|BOLD:AAY6928
Holopyga generosa|BC ZSM HYM 07986|Germany|Rhineland-Palatinate|BOLD:AAY6928
Holopyga generosa|BC ZSM HYM 07987|Germany|Rhineland-Palatinate|BOLD:AAY6928
Holopyga generosa|BC ZSM HYM 12770|Germany|Thuringia|BOLD:ACC3318
Holopyga generosa|BC ZSM HYM 24681|Germany|Saxony-Anhalt|BOLD:ACC3318
Holopyga generosa|BC ZSM HYM 21988|Germany|Saxony-Anhalt|BOLD:ACC3318
Holopyga generosa|BC ZSM HYM 17297|Italy|Aosta Valley|BOLD:ACC3318
Holopyga generosa|BC ZSM HYM 12768|Germany|Thuringia|BOLD:ACC3318
Holopyga generosa|BC ZSM HYM 12769|Germany|Thuringia|BOLD:ACC3318
Holopyga generosa|BC ZSM HYM 13197|Czech Republic||BOLD:ACC3318
Holopyga generosa|BC ZSM HYM 21989|Germany|Saxony-Anhalt|BOLD:ACC3318
Omalus aeneus|KY430827.1_tmp|Hungary||BOLD:ACQ9469
Omalus puncticollis|KY430826.1_tmp|Germany|North Rhine-Westphalia|BOLD:ACC4462
Omalus aeneus|BC ZSM HYM 12915|Italy||BOLD:ACC4462
Omalus aeneus|BC-ZSM-HYM-23654-E05|Slovakia||BOLD:ACG9650
Omalus biaccinctus|KY430828.1_tmp|Germany|North Rhine-Westphalia|BOLD:AFA2340
Omalus biaccinctus|BC ZSM HYM 24679|Germany|Saxony-Anhalt|BOLD:AFA2340
Pseudomalus triangulifer|ON7898|Germany|Hesse
Pseudomalus triangulifer|ON7196|Germany|Mecklenburg-Vorpommern
Pseudomalus triangulifer|ON4528|Germany|Bavaria
Pseudomalus triangulifer|ON11505|Germany|Baden-Wuerttemberg|BOLD:AEC6303
Pseudomalus triangulifer|ON6708|Germany|Rhineland-Palatinate|BOLD:AEC6303
Pseudomalus violaceus|BC ZSM HYM 12107|Germany|Saxony-Anhalt|BOLD:ABX9998
Pseudomalus auratus|BC ZSM HYM 05073|Germany|Bavaria|BOLD:AAH8217
Pseudomalus auratus|BC ZSM HYM 17357|Italy|Aosta Valley|BOLD:AAH8217
Pseudomalus auratus|KY430823.1_tmp|Germany|Rhineland-Palatinate|BOLD:AAH8217
Pseudomalus auratus|BC ZSM HYM 05074|Germany|Bavaria|BOLD:AAH8217
Pseudomalus auratus|BC ZSM HYM 05075|Germany|Bavaria|BOLD:AAH8217
Pseudomalus auratus|BC ZSM HYM 00058|Austria|Tyrol|BOLD:AAH8217
Pseudomalus auratus|BC ZSM HYM 15248|Germany|Brandenburg|BOLD:AAH8217
Pseudomalus auratus|BC ZSM HYM 05076|Austria|Tyrol|BOLD:AAH8217
Pseudomalus auratus|BC ZSM HYM 14122|Germany|Brandenburg|BOLD:AAH8217
Pseudomalus pusillus|BC-ZSM-HYM-23654-F06|Slovakia||BOLD:AAL1775
Pseudomalus pusillus|BC ZSM HYM 14063|Germany|Baden-Wuerttemberg|BOLD:AAL1775
Pseudomalus pusillus|BC ZSM HYM 08036|Germany|Rhineland-Palatinate|BOLD:AAL1775
Pseudomalus pusillus|BC ZSM HYM 08040|Germany|Rhineland-Palatinate|BOLD:AAL1775
Pseudomalus pusillus|BC ZSM HYM 14062|Germany|Baden-Wuerttemberg|BOLD:AAL1775
Pseudomalus pusillus|BC ZSM HYM 17359|Germany|Baden-Wuerttemberg|BOLD:AAL1775
Pseudomalus pusillus|BC ZSM HYM 17358|Germany|Baden-Wuerttemberg|BOLD:AAL1775
Pseudomalus pusillus|BC ZSM HYM 11084|Germany|Baden-Wuerttemberg|BOLD:AAL1775
Pseudomalus pusillus|BC ZSM HYM 11083|Germany|Baden-Wuerttemberg|BOLD:AAL1775
Pseudomalus pusillus|BC ZSM HYM 07407|Germany|Brandenburg|BOLD:AAL1775
Pseudomalus pusillus|BC ZSM HYM 08037|Germany|Rhineland-Palatinate|BOLD:AAL1775
Pseudomalus pusillus|KY430822.1_tmp|Germany|Rhineland-Palatinate|BOLD:AAL1775
Pseudomalus pusillus|BC ZSM HYM 08035|Germany|Rhineland-Palatinate|BOLD:AAL1775
Pseudomalus pusillus|BC ZSM HYM 14064|Germany|Baden-Wuerttemberg|BOLD:AAL1775
Pseudomalus pusillus|BC-ZSM-HYM-23654-F05|Slovakia||BOLD:AAL1775
Pseudomalus pusillus|BC ZSM HYM 14967|Germany|Brandenburg|BOLD:AAL1775
Pseudomalus pusillus|BC ZSM HYM 14968|Germany|Brandenburg|BOLD:AAL1775
Philoctetes putoni|BC ZSM HYM 16243|France|Provence-Alpes-Cote d'Azur|BOLD:ACE0415
Philoctetes bidentulus|BC ZSM HYM 17360|Italy|Aosta Valley|BOLD:AAY9791
Philoctetes bidentulus|BC ZSM HYM 08030|Germany|Rhineland-Palatinate|BOLD:AAY9791
Philoctetes bidentulus|BC ZSM HYM 07990|Germany|Rhineland-Palatinate|BOLD:AAY9791
Philoctetes bidentulus|BC ZSM HYM 07991|Germany|Rhineland-Palatinate|BOLD:AAY9791
Philoctetes bidentulus|BC ZSM HYM 07992|Germany|Rhineland-Palatinate|BOLD:AAY9791
Elampus bidens|KY430834.1_tmp|Italy|Lombardy|BOLD:AEC6927
Elampus panzeri|KY430835.1_tmp|Germany|Rhineland-Palatinate|BOLD:AEA0923
Elampus constrictus|BC ZSM HYM 13190|Czech Republic||BOLD:ACC2184
Elampus konowi|KY430833.1_tmp|Germany|Baden-Wuerttemberg|BOLD:AAY9882
Elampus konowi|BC ZSM HYM 08008|Germany|Baden-Wuerttemberg|BOLD:AAY9882
Elampus konowi|BC ZSM HYM 08006|Germany|Baden-Wuerttemberg|BOLD:AAY9882

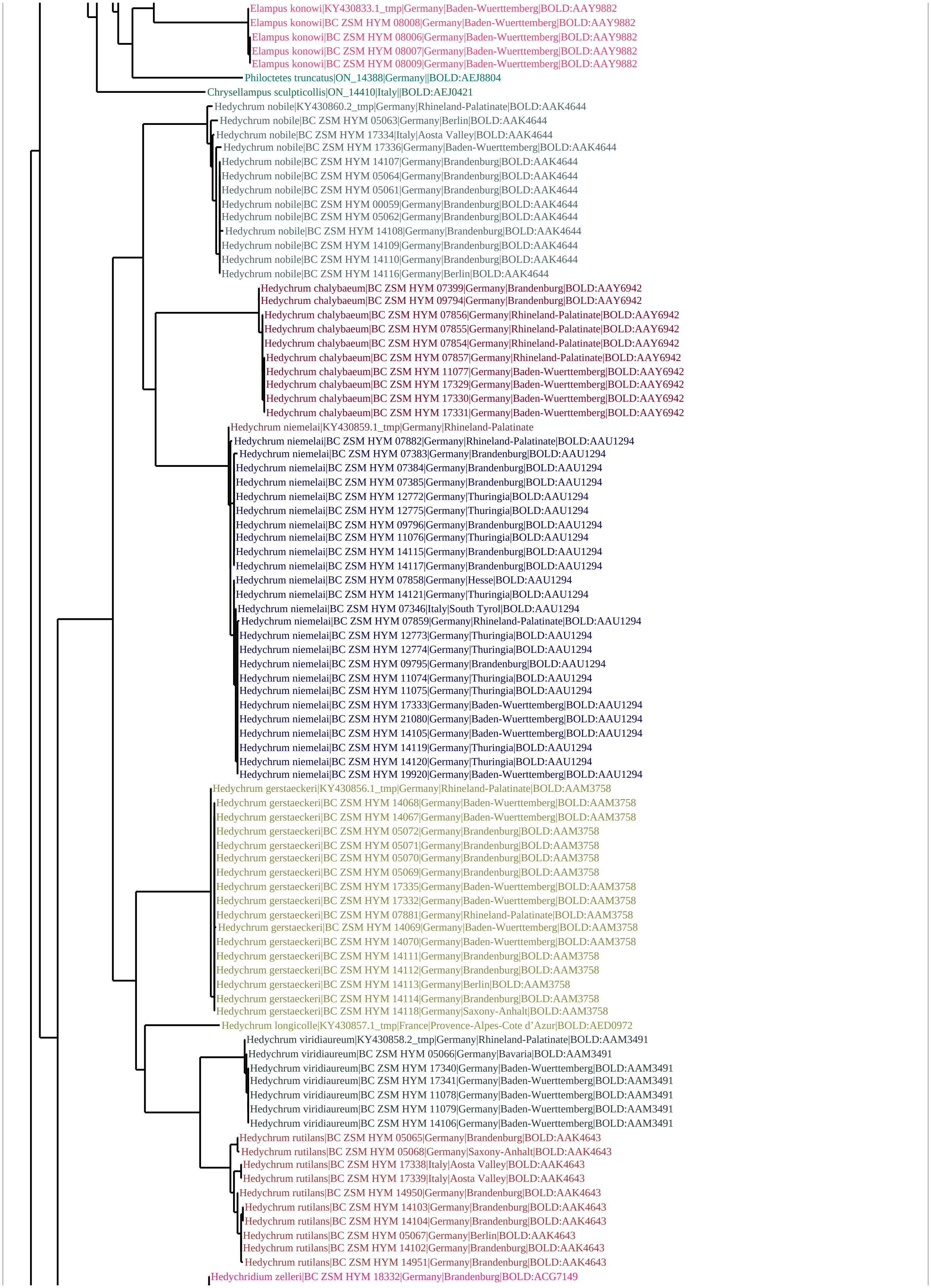
Elampus konowi|KY430833.1_tmp|Germany|Baden-Wuerttemberg|BOLD:AAY9882
Elampus konowi|BC ZSM HYM 08008|Germany|Baden-Wuerttemberg|BOLD:AAY9882
Elampus konowi|BC ZSM HYM 08006|Germany|Baden-Wuerttemberg|BOLD:AAY9882
Elampus konowi|BC ZSM HYM 08007|Germany|Baden-Wuerttemberg|BOLD:AAY9882
Elampus konowi|BC ZSM HYM 08009|Germany|Baden-Wuerttemberg|BOLD:AAY9882
Philoctetes truncatus|ON_14388|Germany||BOLD:AEJ8804
Chrysellampus sculpticollis|ON_14410|Italy||BOLD:AEJ0421
Hedychrum nobile|KY430860.2_tmp|Germany|Rhineland-Palatinate|BOLD:AAK4644
Hedychrum nobile|BC ZSM HYM 05063|Germany|Berlin|BOLD:AAK4644
Hedychrum nobile|BC ZSM HYM 17334|Italy|Aosta Valley|BOLD:AAK4644
Hedychrum nobile|BC ZSM HYM 17336|Germany|Baden-Wuerttemberg|BOLD:AAK4644
Hedychrum nobile|BC ZSM HYM 14107|Germany|Brandenburg|BOLD:AAK4644
Hedychrum nobile|BC ZSM HYM 05064|Germany|Brandenburg|BOLD:AAK4644
Hedychrum nobile|BC ZSM HYM 05061|Germany|Brandenburg|BOLD:AAK4644
Hedychrum nobile|BC ZSM HYM 00059|Germany|Brandenburg|BOLD:AAK4644
Hedychrum nobile|BC ZSM HYM 05062|Germany|Brandenburg|BOLD:AAK4644
Hedychrum nobile|BC ZSM HYM 14108|Germany|Brandenburg|BOLD:AAK4644
Hedychrum nobile|BC ZSM HYM 14109|Germany|Brandenburg|BOLD:AAK4644
Hedychrum nobile|BC ZSM HYM 14110|Germany|Brandenburg|BOLD:AAK4644
Hedychrum nobile|BC ZSM HYM 14116|Germany|Berlin|BOLD:AAK4644
Hedychrum chalybaeum|BC ZSM HYM 07399|Germany|Brandenburg|BOLD:AAY6942
Hedychrum chalybaeum|BC ZSM HYM 09794|Germany|Brandenburg|BOLD:AAY6942
Hedychrum chalybaeum|BC ZSM HYM 07856|Germany|Rhineland-Palatinate|BOLD:AAY6942
Hedychrum chalybaeum|BC ZSM HYM 07855|Germany|Rhineland-Palatinate|BOLD:AAY6942
Hedychrum chalybaeum|BC ZSM HYM 07854|Germany|Rhineland-Palatinate|BOLD:AAY6942
Hedychrum chalybaeum|BC ZSM HYM 07857|Germany|Rhineland-Palatinate|BOLD:AAY6942
Hedychrum chalybaeum|BC ZSM HYM 11077|Germany|Baden-Wuerttemberg|BOLD:AAY6942
Hedychrum chalybaeum|BC ZSM HYM 17329|Germany|Baden-Wuerttemberg|BOLD:AAY6942
Hedychrum chalybaeum|BC ZSM HYM 17330|Germany|Baden-Wuerttemberg|BOLD:AAY6942
Hedychrum chalybaeum|BC ZSM HYM 17331|Germany|Baden-Wuerttemberg|BOLD:AAY6942
Hedychrum niemelai|KY430859.1_tmp|Germany|Rhineland-Palatinate
Hedychrum niemelai|BC ZSM HYM 07882|Germany|Rhineland-Palatinate|BOLD:AAU1294
Hedychrum niemelai|BC ZSM HYM 07383|Germany|Brandenburg|BOLD:AAU1294
Hedychrum niemelai|BC ZSM HYM 07384|Germany|Brandenburg|BOLD:AAU1294
Hedychrum niemelai|BC ZSM HYM 07385|Germany|Brandenburg|BOLD:AAU1294
Hedychrum niemelai|BC ZSM HYM 12772|Germany|Thuringia|BOLD:AAU1294
Hedychrum niemelai|BC ZSM HYM 12775|Germany|Thuringia|BOLD:AAU1294
Hedychrum niemelai|BC ZSM HYM 09796|Germany|Brandenburg|BOLD:AAU1294
Hedychrum niemelai|BC ZSM HYM 11076|Germany|Thuringia|BOLD:AAU1294
Hedychrum niemelai|BC ZSM HYM 14115|Germany|Brandenburg|BOLD:AAU1294
Hedychrum niemelai|BC ZSM HYM 14117|Germany|Brandenburg|BOLD:AAU1294
Hedychrum niemelai|BC ZSM HYM 07858|Germany|Hesse|BOLD:AAU1294
Hedychrum niemelai|BC ZSM HYM 14121|Germany|Thuringia|BOLD:AAU1294
Hedychrum niemelai|BC ZSM HYM 07346|Italy|South Tyrol|BOLD:AAU1294
Hedychrum niemelai|BC ZSM HYM 07859|Germany|Rhineland-Palatinate|BOLD:AAU1294
Hedychrum niemelai|BC ZSM HYM 12773|Germany|Thuringia|BOLD:AAU1294
Hedychrum niemelai|BC ZSM HYM 12774|Germany|Thuringia|BOLD:AAU1294
Hedychrum niemelai|BC ZSM HYM 09795|Germany|Brandenburg|BOLD:AAU1294
Hedychrum niemelai|BC ZSM HYM 11074|Germany|Thuringia|BOLD:AAU1294
Hedychrum niemelai|BC ZSM HYM 11075|Germany|Thuringia|BOLD:AAU1294
Hedychrum niemelai|BC ZSM HYM 17333|Germany|Baden-Wuerttemberg|BOLD:AAU1294
Hedychrum niemelai|BC ZSM HYM 21080|Germany|Baden-Wuerttemberg|BOLD:AAU1294
Hedychrum niemelai|BC ZSM HYM 14105|Germany|Baden-Wuerttemberg|BOLD:AAU1294
Hedychrum niemelai|BC ZSM HYM 14119|Germany|Thuringia|BOLD:AAU1294
Hedychrum niemelai|BC ZSM HYM 14120|Germany|Thuringia|BOLD:AAU1294
Hedychrum niemelai|BC ZSM HYM 19920|Germany|Baden-Wuerttemberg|BOLD:AAU1294
Hedychrum gerstaeckeri|KY430856.1_tmp|Germany|Rhineland-Palatinate|BOLD:AAM3758
Hedychrum gerstaeckeri|BC ZSM HYM 14068|Germany|Baden-Wuerttemberg|BOLD:AAM3758
Hedychrum gerstaeckeri|BC ZSM HYM 14067|Germany|Baden-Wuerttemberg|BOLD:AAM3758
Hedychrum gerstaeckeri|BC ZSM HYM 05072|Germany|Brandenburg|BOLD:AAM3758
Hedychrum gerstaeckeri|BC ZSM HYM 05071|Germany|Brandenburg|BOLD:AAM3758
Hedychrum gerstaeckeri|BC ZSM HYM 05070|Germany|Brandenburg|BOLD:AAM3758
Hedychrum gerstaeckeri|BC ZSM HYM 05069|Germany|Brandenburg|BOLD:AAM3758
Hedychrum gerstaeckeri|BC ZSM HYM 17335|Germany|Baden-Wuerttemberg|BOLD:AAM3758
Hedychrum gerstaeckeri|BC ZSM HYM 17332|Germany|Baden-Wuerttemberg|BOLD:AAM3758
Hedychrum gerstaeckeri|BC ZSM HYM 07881|Germany|Rhineland-Palatinate|BOLD:AAM3758
Hedychrum gerstaeckeri|BC ZSM HYM 14069|Germany|Baden-Wuerttemberg|BOLD:AAM3758
Hedychrum gerstaeckeri|BC ZSM HYM 14070|Germany|Baden-Wuerttemberg|BOLD:AAM3758
Hedychrum gerstaeckeri|BC ZSM HYM 14111|Germany|Brandenburg|BOLD:AAM3758
Hedychrum gerstaeckeri|BC ZSM HYM 14112|Germany|Brandenburg|BOLD:AAM3758
Hedychrum gerstaeckeri|BC ZSM HYM 14113|Germany|Berlin|BOLD:AAM3758
Hedychrum gerstaeckeri|BC ZSM HYM 14114|Germany|Brandenburg|BOLD:AAM3758
Hedychrum gerstaeckeri|BC ZSM HYM 14118|Germany|Saxony-Anhalt|BOLD:AAM3758
Hedychrum longicolle|KY430857.1_tmp|France|Provence-Alpes-Cote d'Azur|BOLD:AED0972
Hedychrum viridiaureum|KY430858.2_tmp|Germany|Rhineland-Palatinate|BOLD:AAM3491
Hedychrum viridiaureum|BC ZSM HYM 05066|Germany|Bavaria|BOLD:AAM3491
Hedychrum viridiaureum|BC ZSM HYM 17340|Germany|Baden-Wuerttemberg|BOLD:AAM3491
Hedychrum viridiaureum|BC ZSM HYM 17341|Germany|Baden-Wuerttemberg|BOLD:AAM3491
Hedychrum viridiaureum|BC ZSM HYM 11078|Germany|Baden-Wuerttemberg|BOLD:AAM3491
Hedychrum viridiaureum|BC ZSM HYM 11079|Germany|Baden-Wuerttemberg|BOLD:AAM3491
Hedychrum viridiaureum|BC ZSM HYM 14106|Germany|Baden-Wuerttemberg|BOLD:AAM3491
Hedychrum rutilans|BC ZSM HYM 05065|Germany|Brandenburg|BOLD:AAK4643
Hedychrum rutilans|BC ZSM HYM 05068|Germany|Saxony-Anhalt|BOLD:AAK4643
Hedychrum rutilans|BC ZSM HYM 17338|Italy|Aosta Valley|BOLD:AAK4643
Hedychrum rutilans|BC ZSM HYM 17339|Italy|Aosta Valley|BOLD:AAK4643
Hedychrum rutilans|BC ZSM HYM 14950|Germany|Brandenburg|BOLD:AAK4643
Hedychrum rutilans|BC ZSM HYM 14103|Germany|Brandenburg|BOLD:AAK4643
Hedychrum rutilans|BC ZSM HYM 14104|Germany|Brandenburg|BOLD:AAK4643
Hedychrum rutilans|BC ZSM HYM 05067|Germany|Berlin|BOLD:AAK4643
Hedychrum rutilans|BC ZSM HYM 14102|Germany|Brandenburg|BOLD:AAK4643
Hedychrum rutilans|BC ZSM HYM 14951|Germany|Brandenburg|BOLD:AAK4643
Hedychridium zelleri|BC ZSM HYM 18332|Germany|Brandenburg|BOLD:ACG7149

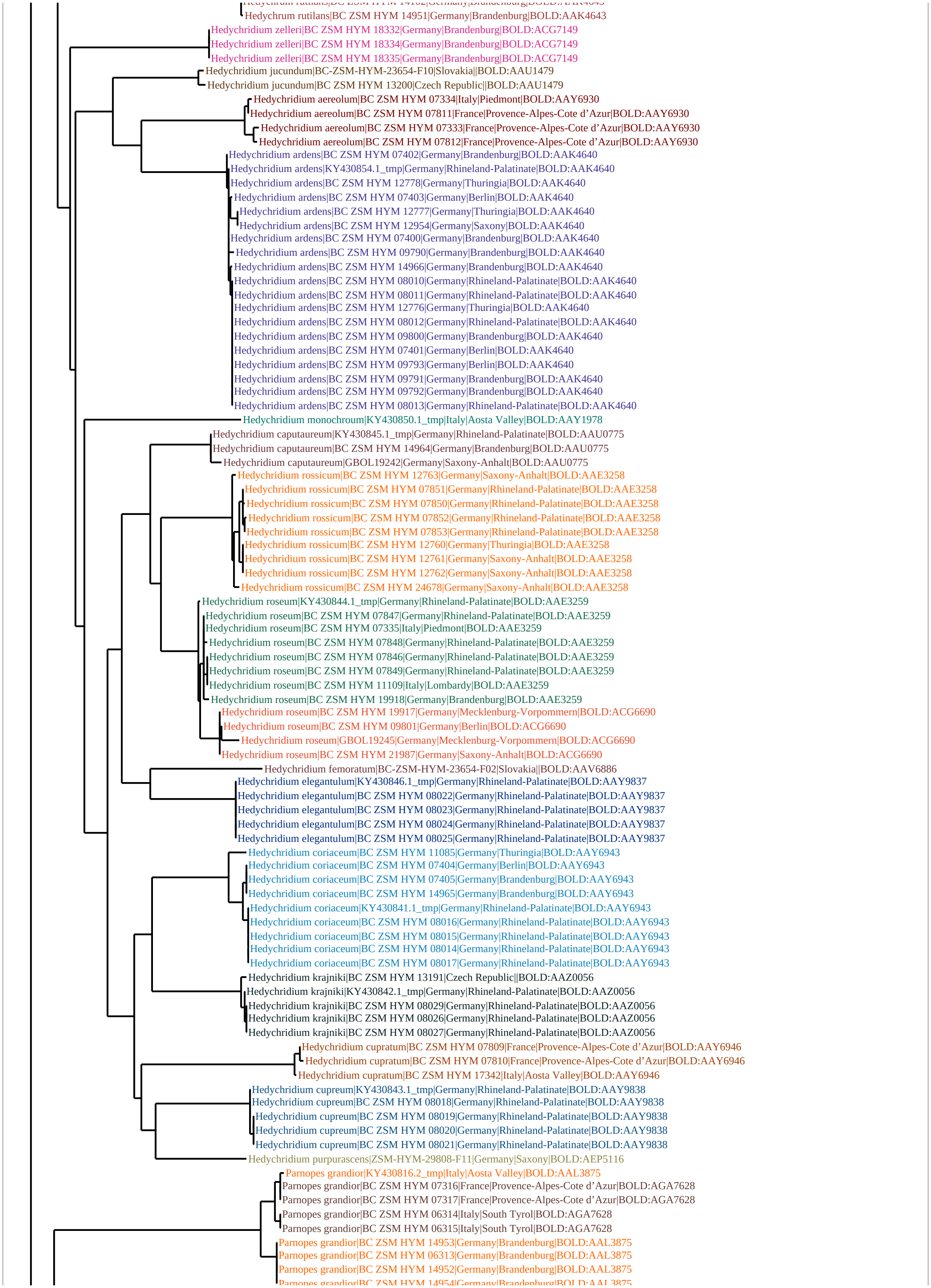
Hedychrum rutilans|BC ZSM HYM 14951|Germany|Brandenburg|BOLD:AAK4643
Hedychridium zelleri|BC ZSM HYM 18332|Germany|Brandenburg|BOLD:ACG7149
Hedychridium zelleri|BC ZSM HYM 18334|Germany|Brandenburg|BOLD:ACG7149
Hedychridium zelleri|BC ZSM HYM 18335|Germany|Brandenburg|BOLD:ACG7149
Hedychridium jucundum|BC-ZSM-HYM-23654-F10|Slovakia||BOLD:AAU1479
Hedychridium jucundum|BC ZSM HYM 13200|Czech Republic||BOLD:AAU1479
Hedychridium aereolum|BC ZSM HYM 07334|Italy|Piedmont|BOLD:AAY6930
Hedychridium aereolum|BC ZSM HYM 07811|France|Provence-Alpes-Cote d’Azur|BOLD:AAY6930
Hedychridium aereolum|BC ZSM HYM 07333|France|Provence-Alpes-Cote d’Azur|BOLD:AAY6930
Hedychridium aereolum|BC ZSM HYM 07812|France|Provence-Alpes-Cote d’Azur|BOLD:AAY6930
Hedychridium ardens|BC ZSM HYM 07402|Germany|Brandenburg|BOLD:AAK4640
Hedychridium ardens|KY430854.1_tmp|Germany|Rhineland-Palatinate|BOLD:AAK4640
Hedychridium ardens|BC ZSM HYM 12778|Germany|Thuringia|BOLD:AAK4640
Hedychridium ardens|BC ZSM HYM 07403|Germany|Berlin|BOLD:AAK4640
Hedychridium ardens|BC ZSM HYM 12777|Germany|Thuringia|BOLD:AAK4640
Hedychridium ardens|BC ZSM HYM 12954|Germany|Saxony|BOLD:AAK4640
Hedychridium ardens|BC ZSM HYM 07400|Germany|Brandenburg|BOLD:AAK4640
Hedychridium ardens|BC ZSM HYM 09790|Germany|Brandenburg|BOLD:AAK4640
Hedychridium ardens|BC ZSM HYM 14966|Germany|Brandenburg|BOLD:AAK4640
Hedychridium ardens|BC ZSM HYM 08010|Germany|Rhineland-Palatinate|BOLD:AAK4640
Hedychridium ardens|BC ZSM HYM 08011|Germany|Rhineland-Palatinate|BOLD:AAK4640
Hedychridium ardens|BC ZSM HYM 12776|Germany|Thuringia|BOLD:AAK4640
Hedychridium ardens|BC ZSM HYM 08012|Germany|Rhineland-Palatinate|BOLD:AAK4640
Hedychridium ardens|BC ZSM HYM 09800|Germany|Brandenburg|BOLD:AAK4640
Hedychridium ardens|BC ZSM HYM 07401|Germany|Berlin|BOLD:AAK4640
Hedychridium ardens|BC ZSM HYM 09793|Germany|Berlin|BOLD:AAK4640
Hedychridium ardens|BC ZSM HYM 09791|Germany|Brandenburg|BOLD:AAK4640
Hedychridium ardens|BC ZSM HYM 09792|Germany|Brandenburg|BOLD:AAK4640
Hedychridium ardens|BC ZSM HYM 08013|Germany|Rhineland-Palatinate|BOLD:AAK4640
Hedychridium monochroum|KY430850.1_tmp|Italy|Aosta Valley|BOLD:AAY1978
Hedychridium caputaureum|KY430845.1_tmp|Germany|Rhineland-Palatinate|BOLD:AAU0775
Hedychridium caputaureum|BC ZSM HYM 14964|Germany|Brandenburg|BOLD:AAU0775
Hedychridium caputaureum|GBOL19242|Germany|Saxony-Anhalt|BOLD:AAU0775
Hedychridium rossicum|BC ZSM HYM 12763|Germany|Saxony-Anhalt|BOLD:AAE3258
Hedychridium rossicum|BC ZSM HYM 07851|Germany|Rhineland-Palatinate|BOLD:AAE3258
Hedychridium rossicum|BC ZSM HYM 07850|Germany|Rhineland-Palatinate|BOLD:AAE3258
Hedychridium rossicum|BC ZSM HYM 07852|Germany|Rhineland-Palatinate|BOLD:AAE3258
Hedychridium rossicum|BC ZSM HYM 07853|Germany|Rhineland-Palatinate|BOLD:AAE3258
Hedychridium rossicum|BC ZSM HYM 12760|Germany|Thuringia|BOLD:AAE3258
Hedychridium rossicum|BC ZSM HYM 12761|Germany|Saxony-Anhalt|BOLD:AAE3258
Hedychridium rossicum|BC ZSM HYM 12762|Germany|Saxony-Anhalt|BOLD:AAE3258
Hedychridium rossicum|BC ZSM HYM 24678|Germany|Saxony-Anhalt|BOLD:AAE3258
Hedychridium roseum|KY430844.1_tmp|Germany|Rhineland-Palatinate|BOLD:AAE3259
Hedychridium roseum|BC ZSM HYM 07847|Germany|Rhineland-Palatinate|BOLD:AAE3259
Hedychridium roseum|BC ZSM HYM 07335|Italy|Piedmont|BOLD:AAE3259
Hedychridium roseum|BC ZSM HYM 07848|Germany|Rhineland-Palatinate|BOLD:AAE3259
Hedychridium roseum|BC ZSM HYM 07846|Germany|Rhineland-Palatinate|BOLD:AAE3259
Hedychridium roseum|BC ZSM HYM 07849|Germany|Rhineland-Palatinate|BOLD:AAE3259
Hedychridium roseum|BC ZSM HYM 11109|Italy|Lombardy|BOLD:AAE3259
Hedychridium roseum|BC ZSM HYM 19918|Germany|Brandenburg|BOLD:AAE3259
Hedychridium roseum|BC ZSM HYM 19917|Germany|Mecklenburg-Vorpommern|BOLD:ACG6690
Hedychridium roseum|BC ZSM HYM 09801|Germany|Berlin|BOLD:ACG6690
Hedychridium roseum|GBOL19245|Germany|Mecklenburg-Vorpommern|BOLD:ACG6690
Hedychridium roseum|BC ZSM HYM 21987|Germany|Saxony-Anhalt|BOLD:ACG6690
Hedychridium femoratum|BC-ZSM-HYM-23654-F02|Slovakia||BOLD:AAV6886
Hedychridium elegantulum|KY430846.1_tmp|Germany|Rhineland-Palatinate|BOLD:AAY9837
Hedychridium elegantulum|BC ZSM HYM 08022|Germany|Rhineland-Palatinate|BOLD:AAY9837
Hedychridium elegantulum|BC ZSM HYM 08023|Germany|Rhineland-Palatinate|BOLD:AAY9837
Hedychridium elegantulum|BC ZSM HYM 08024|Germany|Rhineland-Palatinate|BOLD:AAY9837
Hedychridium elegantulum|BC ZSM HYM 08025|Germany|Rhineland-Palatinate|BOLD:AAY9837
Hedychridium coriaceum|BC ZSM HYM 11085|Germany|Thuringia|BOLD:AAY6943
Hedychridium coriaceum|BC ZSM HYM 07404|Germany|Berlin|BOLD:AAY6943
Hedychridium coriaceum|BC ZSM HYM 07405|Germany|Brandenburg|BOLD:AAY6943
Hedychridium coriaceum|BC ZSM HYM 14965|Germany|Brandenburg|BOLD:AAY6943
Hedychridium coriaceum|KY430841.1_tmp|Germany|Rhineland-Palatinate|BOLD:AAY6943
Hedychridium coriaceum|BC ZSM HYM 08016|Germany|Rhineland-Palatinate|BOLD:AAY6943
Hedychridium coriaceum|BC ZSM HYM 08015|Germany|Rhineland-Palatinate|BOLD:AAY6943
Hedychridium coriaceum|BC ZSM HYM 08014|Germany|Rhineland-Palatinate|BOLD:AAY6943
Hedychridium coriaceum|BC ZSM HYM 08017|Germany|Rhineland-Palatinate|BOLD:AAY6943
Hedychridium krajniki|BC ZSM HYM 13191|Czech Republic||BOLD:AAZ0056
Hedychridium krajniki|KY430842.1_tmp|Germany|Rhineland-Palatinate|BOLD:AAZ0056
Hedychridium krajniki|BC ZSM HYM 08029|Germany|Rhineland-Palatinate|BOLD:AAZ0056
Hedychridium krajniki|BC ZSM HYM 08026|Germany|Rhineland-Palatinate|BOLD:AAZ0056
Hedychridium krajniki|BC ZSM HYM 08027|Germany|Rhineland-Palatinate|BOLD:AAZ0056
Hedychridium cupratum|BC ZSM HYM 07809|France|Provence-Alpes-Cote d’Azur|BOLD:AAY6946
Hedychridium cupratum|BC ZSM HYM 07810|France|Provence-Alpes-Cote d’Azur|BOLD:AAY6946
Hedychridium cupratum|BC ZSM HYM 17342|Italy|Aosta Valley|BOLD:AAY6946
Hedychridium cupreum|KY430843.1_tmp|Germany|Rhineland-Palatinate|BOLD:AAY9838
Hedychridium cupreum|BC ZSM HYM 08018|Germany|Rhineland-Palatinate|BOLD:AAY9838
Hedychridium cupreum|BC ZSM HYM 08019|Germany|Rhineland-Palatinate|BOLD:AAY9838
Hedychridium cupreum|BC ZSM HYM 08020|Germany|Rhineland-Palatinate|BOLD:AAY9838
Hedychridium cupreum|BC ZSM HYM 08021|Germany|Rhineland-Palatinate|BOLD:AAY9838
Hedychridium purpurascens|ZSM-HYM-29808-F11|Germany|Saxony|BOLD:AEP5116
Parnopes grandior|KY430816.2_tmp|Italy|Aosta Valley|BOLD:AAL3875
Parnopes grandior|BC ZSM HYM 07316|France|Provence-Alpes-Cote d’Azur|BOLD:AGA7628
Parnopes grandior|BC ZSM HYM 07317|France|Provence-Alpes-Cote d’Azur|BOLD:AGA7628
Parnopes grandior|BC ZSM HYM 06314|Italy|South Tyrol|BOLD:AGA7628
Parnopes grandior|BC ZSM HYM 06315|Italy|South Tyrol|BOLD:AGA7628
Parnopes grandior|BC ZSM HYM 14953|Germany|Brandenburg|BOLD:AAL3875
Parnopes grandior|BC ZSM HYM 06313|Germany|Brandenburg|BOLD:AAL3875
Parnopes grandior|BC ZSM HYM 14952|Germany|Brandenburg|BOLD:AAL3875
Parnopes grandior|BC ZSM HYM 14954|Germany|Brandenburg|BOLD:AAL3875

Parnopes grandior|BC ZSM HYM 06313|Germany|Brandenburg|BOLD:AAL3875
Parnopes grandior|BC ZSM HYM 14952|Germany|Brandenburg|BOLD:AAL3875
Parnopes grandior|BC ZSM HYM 14954|Germany|Brandenburg|BOLD:AAL3875
Cleptes striatipleuris|KY430863.1_tmp|Germany|Rhineland-Palatinate|BOLD:AAU2238
Cleptes semiauratus|KY430862.1_tmp|Germany|Rhineland-Palatinate|BOLD:AAJ3895
Cleptes semiauratus|BC ZSM HYM 05079|Germany|Bavaria|BOLD:AAJ3895
Cleptes semiauratus|BC ZSM HYM 05080|Germany|Bavaria|BOLD:AAJ3895
Cleptes semiauratus|BC ZSM HYM 20587|Germany|Baden-Wuerttemberg|BOLD:AAM4041
Cleptes semiauratus|BC ZSM HYM 05077|Germany|Bavaria|BOLD:AAM4041
Cleptes semiauratus|BC ZSM HYM 05078|Germany|Bavaria|BOLD:AAM4041
Cleptes semiauratus|BC ZSM HYM 14123|Germany|Bavaria|BOLD:AAM4041
Cleptes splendidus|KY430861.1_tmp|Germany|Rhineland-Palatinate|BOLD:AAR9529
Cleptes splendidus|BC ZSM HYM 08004|Germany|Rhineland-Palatinate|BOLD:AAR9529
Cleptes nitidulus|BC ZSM HYM 08002|Germany|Rhineland-Palatinate|BOLD:AAZ1160
Cleptes nitidulus|BC ZSM HYM 24684|Germany|Saxony-Anhalt|BOLD:AAZ1160
Cleptes nitidulus|BC ZSM HYM 24683|Germany|Saxony-Anhalt|BOLD:AAZ1160
Cleptes nitidulus|BC ZSM HYM 24682|Germany|Saxony-Anhalt|BOLD:AAZ1160
Cleptes nitidulus|BC ZSM HYM 08003|Germany|Rhineland-Palatinate|BOLD:AAZ1160
Cleptes nitidulus|BC ZSM HYM 08005|Germany|Rhineland-Palatinate|BOLD:AAZ1160
Chrysis solida|BC-ZSM-HYM-29771-E02|Germany|Brandenburg|BOLD:AAY6949
Chrysis solida|BC-ZSM-HYM-29774-E09|Germany|Berlin|BOLD:AAY6949
Chrysis solida|ON7665|Italy|Emilia-Romagna|BOLD:AAY6949
Chrysis solida|ON7536|Italy|Aosta Valley|BOLD:AAY6949
Chrysis solida|ON4803|Germany|Brandenburg|BOLD:AAY6949
Chrysis solida|ON7667|Italy|Aosta Valley|BOLD:AAY6949
Chrysis solida|ON7669|Italy|Aosta Valley|BOLD:AAY6949
Chrysis solida|ON9120|Germany|Baden-Wuerttemberg|BOLD:AAY6949
Chrysis solida|KY430766.1_tmp|Germany|Schleswig-Holstein|BOLD:AAY6949
Chrysis solida|ON7197|Germany|Mecklenburg-Vorpommern|BOLD:AAY6949
Chrysis solida|ON7189|Germany|Mecklenburg-Vorpommern|BOLD:AAY6949
Chrysis solida|BC-ZSM-HYM-29774-H07|Germany|Saxony|BOLD:AAY6949
Chrysis solida|BC-ZSM-HYM-29771-D11|Germany|Thuringia|BOLD:AAY6949
Chrysis solida|ON7664|Italy|Emilia-Romagna|BOLD:AAY6949
Chrysis solida|ON7535|Italy|Aosta Valley|BOLD:AAY6949
Chrysis solida|BC-ZSM-HYM-29774-E10|Germany||BOLD:AAY6949
Chrysis solida|BC-ZSM-HYM-29774-H06|Germany|Saxony|BOLD:AAY6949
Chrysis solida|ON9764|Germany|Baden-Wuerttemberg|BOLD:AAY6949
Chrysis solida|ON7775|Germany|Bavaria|BOLD:AAY6949
Chrysis solida|ON0112|Germany|Rhineland-Palatinate|BOLD:AAY6949
Chrysis solida|ON5248|Sweden|Skane|BOLD:AAY6949
Chrysis solida|BC ZSM HYM 12754|Germany|Thuringia|BOLD:AAY6949
Chrysis solida|BC ZSM HYM 12753|Germany|Thuringia|BOLD:AAY6949
Chrysis solida|BC ZSM HYM 12752|Germany|Thuringia|BOLD:AAY6949
Chrysis solida|BC-ZSM-HYM-29774-G07|Germany||BOLD:AAY6949
Chrysis solida|BC-ZSM-HYM-29771-D10|Germany|Thuringia|BOLD:AAY6949
Chrysis mediata|ON5538|Germany|Bavaria|BOLD:AAY6949
Chrysis mediata|ON0748|Germany|Rhineland-Palatinate|BOLD:AAY6949
Chrysis mediata|BC-ZSM-HYM-29774-H09|Germany||BOLD:AAY6949
Chrysis mediata|BC-ZSM-HYM-29774-G10|Germany||BOLD:AAY6949
Chrysis mediata|BC ZSM HYM 12154|Germany|Saxony-Anhalt|BOLD:AAY6949
Chrysis mediata|ON2244|Germany|Baden-Wuerttemberg|BOLD:AAY6949
Chrysis mediata|ON4629|Germany|Baden-Wuerttemberg|BOLD:AAY6949
Chrysis mediata|ON0605|Germany|Rhineland-Palatinate|BOLD:AAY6949
Chrysis mediata|ON0122|Germany|Rhineland-Palatinate|BOLD:AAY6949
Chrysis mediata|BC ZSM HYM 07862|Germany|Rhineland-Palatinate|BOLD:AAY6949
Chrysis mediata|BC ZSM HYM 07863|Germany|Rhineland-Palatinate|BOLD:AAY6949
Chrysis mediata|BC ZSM HYM 07864|Germany|Rhineland-Palatinate|BOLD:AAY6949
Chrysis mediata|BC ZSM HYM 07865|Germany|Rhineland-Palatinate|BOLD:AAY6949
Chrysis mediata|ON1867|Germany|Baden-Wuerttemberg|BOLD:AAY6949
Chrysis mediata|ON0463|Germany|Rhineland-Palatinate|BOLD:AAY6949
Chrysis mediata|ON6150|Germany|Hesse|BOLD:AAY6949
Chrysis mediata|ON7924|Germany|Baden-Wuerttemberg|BOLD:AAY6949
Chrysis mediata|ON1489|Germany|Rhineland-Palatinate|BOLD:AAY6949
Chrysis mediata|KY430767.1_tmp|Italy|Aosta Valley|BOLD:AAY6949
Chrysis schencki|ON9719|Germany|Baden-Wuerttemberg|BOLD:ACF6219
Chrysis schencki|ON11515|Germany|Baden-Wuerttemberg|BOLD:ACF6219
Chrysis schencki|ON11496|Germany|Baden-Wuerttemberg|BOLD:ACF6219
Chrysis schencki|ON11110|Germany|Baden-Wuerttemberg|BOLD:ACF6219
Chrysis schencki|ON11085|Germany|Baden-Wuerttemberg|BOLD:ACF6219
Chrysis schencki|ON11079|Germany|Baden-Wuerttemberg|BOLD:ACF6219
Chrysis schencki|ON11413|Germany|Baden-Wuerttemberg|BOLD:ACF6219
Chrysis schencki|ON9713|Germany|Baden-Wuerttemberg|BOLD:ACF6219
Chrysis schencki|ON9712|Germany|Baden-Wuerttemberg|BOLD:ACF6219
Chrysis schencki|ON9738|Germany|Baden-Wuerttemberg|BOLD:ACF6219
Chrysis schencki|ON9789|Germany|Baden-Wuerttemberg|BOLD:ACF6219
Chrysis schencki|ON11395|Germany|Baden-Wuerttemberg|BOLD:ACF6219
Chrysis schencki|ON11201|Germany|Baden-Wuerttemberg|BOLD:ACF6219
Chrysis schencki|ON11492|Germany|Baden-Wuerttemberg|BOLD:ACF6219
Chrysis schencki|ON11491|Germany|Baden-Wuerttemberg|BOLD:ACF6219
Chrysis schencki|ON11490|Germany|Baden-Wuerttemberg|BOLD:ACF6219
Chrysis schencki|ON11489|Germany|Baden-Wuerttemberg|BOLD:ACF6219
Chrysis schencki|ON11488|Germany|Baden-Wuerttemberg|BOLD:ACF6219
Chrysis schencki|ON11535|Germany|Baden-Wuerttemberg|BOLD:ACF6219
Chrysis schencki|ON9711|Germany|Baden-Wuerttemberg|BOLD:ACF6219
Chrysis schencki|ON11531|Germany|Baden-Wuerttemberg|BOLD:ACF6219
Chrysis schencki|ON11523|Germany|Baden-Wuerttemberg|BOLD:ACF6219
Chrysis schencki|ON11516|Germany|Baden-Wuerttemberg|BOLD:ACF6219
Chrysis schencki|ON11469|Germany|Baden-Wuerttemberg|BOLD:ACF6219
Chrysis schencki|ON11078|Germany|Baden-Wuerttemberg|BOLD:ACF6219
Chrysis schencki|BC-ZSM-HYM-29774-G05|Germany||BOLD:ACF6219
Chrysis schencki|BC-ZSM-HYM-29774-F03|Germany||BOLD:ACF6219
Chrysis schencki|BC-ZSM-HYM-29774-E12|Germany|Berlin|BOLD:ACF6219

Chrysis schencki|BC-ZSM-HYM-29774-G05|Germany||BOLD:ACF6219
Chrysis schencki|BC-ZSM-HYM-29774-F03|Germany||BOLD:ACF6219
Chrysis schencki|BC-ZSM-HYM-29774-E12|Germany|Berlin|BOLD:ACF6219
Chrysis schencki|BC-ZSM-HYM-29774-F01|Germany|Berlin|BOLD:ACF6219
Chrysis schencki|BC-ZSM-HYM-29774-H11|Germany||BOLD:ACF6219
Chrysis schencki|KY430764.1_tmp|Sweden|Kalmar|BOLD:ACF6219
Chrysis schencki|BC ZSM HYM 12759|Germany|Thuringia|BOLD:ACF6219
Chrysis schencki|ON9717|Germany|Baden-Wuerttemberg|BOLD:ACF6219
Chrysis schencki|ON9737|Germany|Baden-Wuerttemberg|BOLD:ACF6219
Chrysis schencki|BC-ZSM-HYM-29774-E11|Germany|Berlin|BOLD:ACF6219
Chrysis schencki|BC-ZSM-HYM-29771-H11|Germany|Brandenburg|BOLD:ACF6219
Chrysis schencki|ON11387|Germany|Baden-Wuerttemberg|BOLD:ABU6375
Chrysis schencki|ON11502|Germany|Baden-Wuerttemberg|BOLD:ABU6375
Chrysis schencki|ON9715|Germany|Baden-Wuerttemberg|BOLD:ABU6375
Chrysis schencki|ON9725|Germany|Baden-Wuerttemberg|BOLD:ABU6375
Chrysis schencki|ON11472|Germany|Baden-Wuerttemberg|BOLD:ABU6375
Chrysis schencki|ON9718|Germany|Baden-Wuerttemberg|BOLD:ABU6375
Chrysis schencki|ON10498|Germany|Baden-Wuerttemberg|BOLD:ABU6375
Chrysis schencki|ON11112|Germany|Baden-Wuerttemberg|BOLD:ABU6375
Chrysis schencki|BC-ZSM-HYM-29774-G04|Germany|Saxony|BOLD:ABU6375
Chrysis parietis|ON3462|Hungary|Gyor-Moson-Sopron|BOLD:AAU2329
Chrysis parietis|ON3468|Germany|Baden-Wuerttemberg
Chrysis parietis|ON3469|Germany|Baden-Wuerttemberg|BOLD:AAU2329
Chrysis parietis|ON7396|Germany|Baden-Wuerttemberg|BOLD:AAU2329
Chrysis parietis|ON1473|Germany|Rhineland-Palatinate|BOLD:AAU2329
Chrysis parietis|BC-ZSM-HYM-29771-E03|Germany|Brandenburg|BOLD:AAU2329
Chrysis parietis|ON_14624|Germany|Baden-Wuerttemberg|BOLD:AAU2329
Chrysis parietis|BC ZSM HYM 08000|Germany|Rhineland-Palatinate|BOLD:AAU2329
Chrysis parietis|BC-ZSM-HYM-29774-H01|Germany|Saxony|BOLD:AAU2329
Chrysis parietis|BC-ZSM-HYM-29774-F07|Germany|Berlin|BOLD:AAU2329
Chrysis parietis|BC-ZSM-HYM-29774-H10|Germany||BOLD:AAU2329
Chrysis parietis|BC ZSM HYM 07998|Germany|Rhineland-Palatinate|BOLD:AAU2329
Chrysis parietis|ON_14623|Germany|Baden-Wuerttemberg|BOLD:AAU2329
Chrysis leptomandibularis|BC-ZSM-HYM-29774-F04|Germany|Berlin|BOLD:ACQ4597
Chrysis leptomandibularis|KY430765.1_tmp|Italy|Lombardy|BOLD:ACQ4597
Chrysis terminata|GBOL 01977|France|Auvergne-Rhones-Alpes|BOLD:ABY5626
Chrysis terminata|BC-ZSM-HYM-29774-H02|Germany||BOLD:ABY5626
Chrysis terminata|BC ZSM HYM 11091|Germany|Thuringia|BOLD:ABY5626
Chrysis terminata|BC-ZSM-HYM-29774-H08|Germany|Saxony|BOLD:ABY5626
Chrysis terminata|KY430760.1_tmp|Germany|Rhineland-Palatinate|BOLD:ABY5626
Chrysis terminata|ON9404|Germany|Baden-Wuerttemberg|BOLD:ABY5626
Chrysis terminata|ON7242|Germany|North Rhine-Westphalia|BOLD:ABY5626
Chrysis terminata|ON5241|Sweden|Kalmar|BOLD:ABY5626
Chrysis terminata|ON7214|Germany|North Rhine-Westphalia|BOLD:ABY5626
Chrysis terminata|BC-ZSM-HYM-29774-F12|Germany|North Rhine-Westphalia|BOLD:ABY5626
Chrysis terminata|BC ZSM HYM 12749|Germany|Thuringia|BOLD:ABY5626
Chrysis terminata|BC ZSM HYM 12738|Germany|Thuringia|BOLD:ABY5626
Chrysis terminata|BC-ZSM-HYM-29774-F02|Germany|Berlin|BOLD:ABY5626
Chrysis terminata|BC-ZSM-HYM-29771-E08|Germany|Baden-Wuerttemberg|BOLD:ABY5626
Chrysis terminata|BC-ZSM-HYM-29774-F11|Germany|Saxony|BOLD:ABY5626
Chrysis terminata|BC-ZSM-HYM-29774-F10|Germany|Berlin|BOLD:ABY5626
Chrysis terminata|BC-ZSM-HYM-29774-G12|Germany|Saxony|BOLD:ABY5626
Chrysis terminata|BC-ZSM-HYM-29771-E04|Germany|Saxony-Anhalt|BOLD:ABY5626
Chrysis terminata|BC ZSM HYM 11092|Germany|Thuringia|BOLD:ABY5626
Chrysis terminata|BC ZSM HYM 12739|Germany|Thuringia|BOLD:ABY5626
Chrysis terminata|BC ZSM HYM 12740|Germany|Thuringia|BOLD:ABY5626
Chrysis terminata|BC ZSM HYM 12741|Germany|Thuringia|BOLD:ABY5626
Chrysis terminata|BC-ZSM-HYM-29771-E07|Germany|Saxony-Anhalt|BOLD:ABY5626
Chrysis terminata|BC-ZSM-HYM-27533-B10|Germany|Bavaria|BOLD:ABY5626
Chrysis terminata|ON7569|Italy|Aosta Valley|BOLD:ABY5626
Chrysis terminata|ON5953|Germany|Hesse|BOLD:ABY5626
Chrysis terminata|BC-ZSM-HYM-29771-H09|Germany|Berlin|BOLD:ABY5626
Chrysis terminata|BC-ZSM-HYM-27533-B12|Germany|Bavaria|BOLD:ABY5626
Chrysis impressa|ON9041|Netherlands|Gelderland|BOLD:AAG0244
Chrysis impressa|ON9033|Netherlands|Gelderland|BOLD:AAG0244
Chrysis impressa|KY430763.1_tmp|Sweden|Kalmar|BOLD:AAG0244
Chrysis impressa|ON8138|Germany|Hesse|BOLD:AAG0244
Chrysis impressa|ON8207|Germany|Hesse|BOLD:AAG0244
Chrysis impressa|BC-ZSM-HYM-29774-H05|Germany||BOLD:AAG0244
Chrysis impressa|BC ZSM HYM 12751|Germany|Thuringia|BOLD:AAG0244
Chrysis impressa|BC ZSM HYM 12743|Germany|Thuringia|BOLD:AAG0244
Chrysis impressa|BC ZSM HYM 07999|Germany|Hesse|BOLD:AAG0244
Chrysis ignita|ON2127|Germany|Baden-Wuerttemberg|BOLD:AAG0244
Chrysis ignita|ON4360|Germany|Rhineland-Palatinate|BOLD:AAG0244
Chrysis ignita|ON0609|Germany|Rhineland-Palatinate|BOLD:AAG0244
Chrysis ignita|KY430761.1_tmp|Germany|Rhineland-Palatinate|BOLD:AAG0244
Chrysis ignita|ON0759|Germany|Rhineland-Palatinate|BOLD:AAG0244
Chrysis ignita|ON0752|Germany|Rhineland-Palatinate|BOLD:AAG0244
Chrysis ignita|ON0613|Germany|Rhineland-Palatinate|BOLD:AAG0244
Chrysis ignita|ON0614|Germany|Rhineland-Palatinate|BOLD:AAG0244
Chrysis ignita|ON0756|Germany|Rhineland-Palatinate|BOLD:AAG0244
Chrysis ignita|ON6146|Germany|Hesse|BOLD:AAG0244
Chrysis ignita|ON6945|Germany|Rhineland-Palatinate|BOLD:AAG0244
Chrysis pseudobrevitarsis|ON4612|Germany|Bavaria|BOLD:ACG6983
Chrysis pseudobrevitarsis|ON4619|Germany|Bavaria|BOLD:ACG6983
Chrysis pseudobrevitarsis|ON7777|Germany|Bavaria|BOLD:ACG6983
Chrysis pseudobrevitarsis|ON9821|Germany|Mecklenburg-Vorpommern|BOLD:ACG6983
Chrysis brevitarsis|KU854911|||BOLD:ACG7211
Chrysis brevitarsis|JX292241|Sweden||BOLD:ACG7211
Chrysis parabrevitarsis|ON4673|Germany|Hesse|BOLD:ACF7346
Chrysis parabrevitarsis|ON5964|Germany|Rhineland-Palatinate|BOLD:ACG6749
Chrysis parabrevitarsis|ON8235|Germany|Rhineland-Palatinate|BOLD:ACG6749
Chrysis parabrevitarsis|ON7911|Germany|Rhineland-Palatinate|BOLD:ACG6749

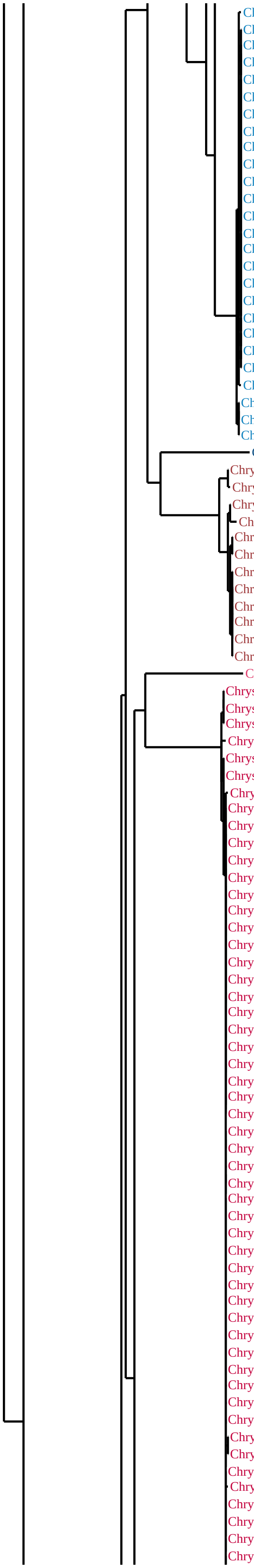

Chrysis parabrevitarsis|ON5964|Germany|Rhineland-Palatinate|BOLD:ACG6749
Chrysis parabrevitarsis|ON8235|Germany|Rhineland-Palatinate|BOLD:ACG6749
Chrysis parabrevitarsis|ON7911|Germany|Rhineland-Palatinate|BOLD:ACG6749
Chrysis parabrevitarsis|KY430768.1_tmp|Germany|Rhineland-Palatinate|BOLD:ACG6749
Chrysis parabrevitarsis|ON6006|Germany|Rhineland-Palatinate|BOLD:ACG6749
Chrysis parabrevitarsis|ON4679|Germany|Hesse|BOLD:ACG6749
Chrysis parabrevitarsis|ON4657|Germany|Hesse|BOLD:ACG6749
Chrysis parabrevitarsis|ON4560|Germany|Bavaria|BOLD:ACG6749
Chrysis parabrevitarsis|ON4559|Germany|Bavaria|BOLD:ACG6749
Chrysis parabrevitarsis|ON4445|Germany|Rhineland-Palatinate|BOLD:ACG6749
Chrysis parabrevitarsis|ON4440|Germany|Rhineland-Palatinate|BOLD:ACG6749
Chrysis parabrevitarsis|ON4394|Germany|Hesse|BOLD:ACG6749
Chrysis parabrevitarsis|ON3242|Germany|Rhineland-Palatinate|BOLD:ACG6749
Chrysis parabrevitarsis|ON3197|Germany|Rhineland-Palatinate|BOLD:ACG6749
Chrysis parabrevitarsis|ON3194|Germany|Rhineland-Palatinate|BOLD:ACG6749
Chrysis parabrevitarsis|ON3143|Germany|Rhineland-Palatinate|BOLD:ACG6749
Chrysis parabrevitarsis|ON6955|Germany|Hesse|BOLD:ACG6749
Chrysis parabrevitarsis|ON6711|Germany|Rhineland-Palatinate|BOLD:ACG6749
Chrysis parabrevitarsis|ON5349|Germany|Hesse|BOLD:ACG6749
Chrysis parabrevitarsis|ON4557|Germany|Bavaria|BOLD:ACG6749
Chrysis parabrevitarsis|ON4411|Germany|Hesse|BOLD:ACG6749
Chrysis parabrevitarsis|BC ZSM HYM 12742|Germany|Thuringia|BOLD:ACG6749
Chrysis parabrevitarsis|BC ZSM HYM 12748|Germany|Thuringia|BOLD:ACG6749
Chrysis parabrevitarsis|ON4369|Germany|Rhineland-Palatinate|BOLD:ACG6749
Chrysis parabrevitarsis|ON4668|Germany|Hesse|BOLD:ACG6749
Chrysis parabrevitarsis|BC ZSM HYM 12745|Germany|Thuringia|BOLD:ACG6749
Chrysis sculpturata|ON1269|France|Provence-Alpes-Cote d'Azur|BOLD:ABU6373
Chrysis horridula|BC ZSM HYM 15251|Germany|Brandenburg|BOLD:AAU2328
Chrysis horridula|BC ZSM HYM 12747|Germany|Thuringia|BOLD:AAU2328
Chrysis horridula|ON1257|France|Provence-Alpes-Cote d'Azur|BOLD:AAU2328
Chrysis horridula|BC-ZSM-HYM-29774-F06|Germany|Berlin|BOLD:AAU2328
Chrysis horridula|BC-ZSM-HYM-29774-F09|Germany|Saxony|BOLD:AAU2328
Chrysis horridula|BC-ZSM-HYM-29771-E06|Germany|Saxony-Anhalt|BOLD:AAU2328
Chrysis horridula|BC-ZSM-HYM-29774-G09|Germany||BOLD:AAU2328
Chrysis horridula|BC-ZSM-HYM-29774-F08|Germany|Saxony|BOLD:AAU2328
Chrysis horridula|BC-ZSM-HYM-29774-G08|Germany||BOLD:AAU2328
Chrysis horridula|BC-ZSM-HYM-29771-E01|Germany|Saxony-Anhalt|BOLD:AAU2328
Chrysis horridula|BC ZSM HYM 12746|Germany|Thuringia|BOLD:AAU2328
Chrysis horridula|BC ZSM HYM 19913|Germany|Saxony-Anhalt|BOLD:AAU2328
Chrysis immaculata|KY430762.1_tmp|Germany|North Rhine-Westphalia|BOLD:AEC7328
Chrysis corusca|ON9734|Germany|Baden-Wuerttemberg|BOLD:ACF7605
Chrysis corusca|ON9710|Germany|Baden-Wuerttemberg|BOLD:ACF7605
Chrysis corusca|ON9735|Germany|Baden-Wuerttemberg|BOLD:ACF7605
Chrysis corusca|ON11421|Germany|Baden-Wuerttemberg|BOLD:ACF7605
Chrysis corusca|ON10534|Germany|Baden-Wuerttemberg|BOLD:ACF7605
Chrysis corusca|BC-ZSM-HYM-29771-D12|Germany|Hamburg|BOLD:ACF7605
Chrysis corusca|ON5232|Sweden|Kalmar|BOLD:ACF7605
Chrysis corusca|ON11480|Germany|Baden-Wuerttemberg|BOLD:ACF7605
Chrysis corusca|ON10547|Germany|Baden-Wuerttemberg|BOLD:ACF7605
Chrysis corusca|ON11084|Germany|Baden-Wuerttemberg|BOLD:ACF7605
Chrysis corusca|ON11206|Germany|Baden-Wuerttemberg|BOLD:ACF7605
Chrysis corusca|ON9793|Germany|Baden-Wuerttemberg|BOLD:ACF7605
Chrysis corusca|ON9792|Germany|Baden-Wuerttemberg|BOLD:ACF7605
Chrysis corusca|ON9786|Germany|Baden-Wuerttemberg|BOLD:ACF7605
Chrysis corusca|ON10493|Germany|Baden-Wuerttemberg|BOLD:ACF7605
Chrysis corusca|ON10497|Germany|Baden-Wuerttemberg|BOLD:ACF7605
Chrysis corusca|ON9785|Germany|Baden-Wuerttemberg|BOLD:ACF7605
Chrysis corusca|ON10538|Germany|Baden-Wuerttemberg|BOLD:ACF7605
Chrysis corusca|ON11111|Germany|Baden-Wuerttemberg|BOLD:ACF7605
Chrysis corusca|ON11075|Germany|Baden-Wuerttemberg|BOLD:ACF7605
Chrysis corusca|ON11466|Germany|Baden-Wuerttemberg|BOLD:ACF7605
Chrysis corusca|ON11477|Germany|Baden-Wuerttemberg|BOLD:ACF7605
Chrysis corusca|ON11506|Germany|Baden-Wuerttemberg|BOLD:ACF7605
Chrysis corusca|ON11508|Germany|Baden-Wuerttemberg|BOLD:ACF7605
Chrysis corusca|ON11518|Germany|Baden-Wuerttemberg|BOLD:ACF7605
Chrysis corusca|ON11532|Germany|Baden-Wuerttemberg|BOLD:ACF7605
Chrysis corusca|ON11204|Germany|Baden-Wuerttemberg|BOLD:ACF7605
Chrysis corusca|ON11205|Germany|Baden-Wuerttemberg|BOLD:ACF7605
Chrysis corusca|ON11194|Germany|Baden-Wuerttemberg|BOLD:ACF7605
Chrysis corusca|ON11202|Germany|Baden-Wuerttemberg|BOLD:ACF7605
Chrysis corusca|KY430769.1_tmp|Germany|Hesse|BOLD:ACF7605
Chrysis corusca|ON11198|Germany|Baden-Wuerttemberg|BOLD:ACF7605
Chrysis corusca|ON11192|Germany|Baden-Wuerttemberg|BOLD:ACF7605
Chrysis corusca|ON11114|Germany|Baden-Wuerttemberg|BOLD:ACF7605
Chrysis corusca|ON11188|Germany|Baden-Wuerttemberg|BOLD:ACF7605
Chrysis corusca|ON9736|Germany|Baden-Wuerttemberg|BOLD:ACF7605
Chrysis corusca|ON9739|Germany|Baden-Wuerttemberg|BOLD:ACF7605
Chrysis corusca|ON9720|Germany|Baden-Wuerttemberg|BOLD:ACF7605
Chrysis corusca|ON9781|Germany|Baden-Wuerttemberg|BOLD:ACF7605
Chrysis corusca|ON9733|Germany|Baden-Wuerttemberg|BOLD:ACF7605
Chrysis corusca|ON9731|Germany|Baden-Wuerttemberg|BOLD:ACF7605
Chrysis corusca|ON9740|Germany|Baden-Wuerttemberg|BOLD:ACF7605
Chrysis corusca|ON9724|Germany|Baden-Wuerttemberg|BOLD:ACF7605
Chrysis corusca|ON9779|Germany|Baden-Wuerttemberg|BOLD:ACF7605
Chrysis corusca|ON11199|Germany|Baden-Wuerttemberg|BOLD:ACF7605
Chrysis corusca|ON11210|Germany|Baden-Wuerttemberg|BOLD:ACF7605
Chrysis corusca|ON1700|Germany|Rhineland-Palatinate|BOLD:ACF7605
Chrysis corusca|BC-ZSM-HYM-29774-G01|Germany|North Rhine-Westphalia|BOLD:ACF7605
Chrysis corusca|BC-ZSM-HYM-29774-G02|Germany|North Rhine-Westphalia|BOLD:ACF7605
Chrysis corusca|ON9721|Germany|Baden-Wuerttemberg|BOLD:ACF7605
Chrysis corusca|ON11203|Germany|Baden-Wuerttemberg|BOLD:ACF7605
Chrysis corusca|ON9778|Germany|Baden-Wuerttemberg|BOLD:ACF7605

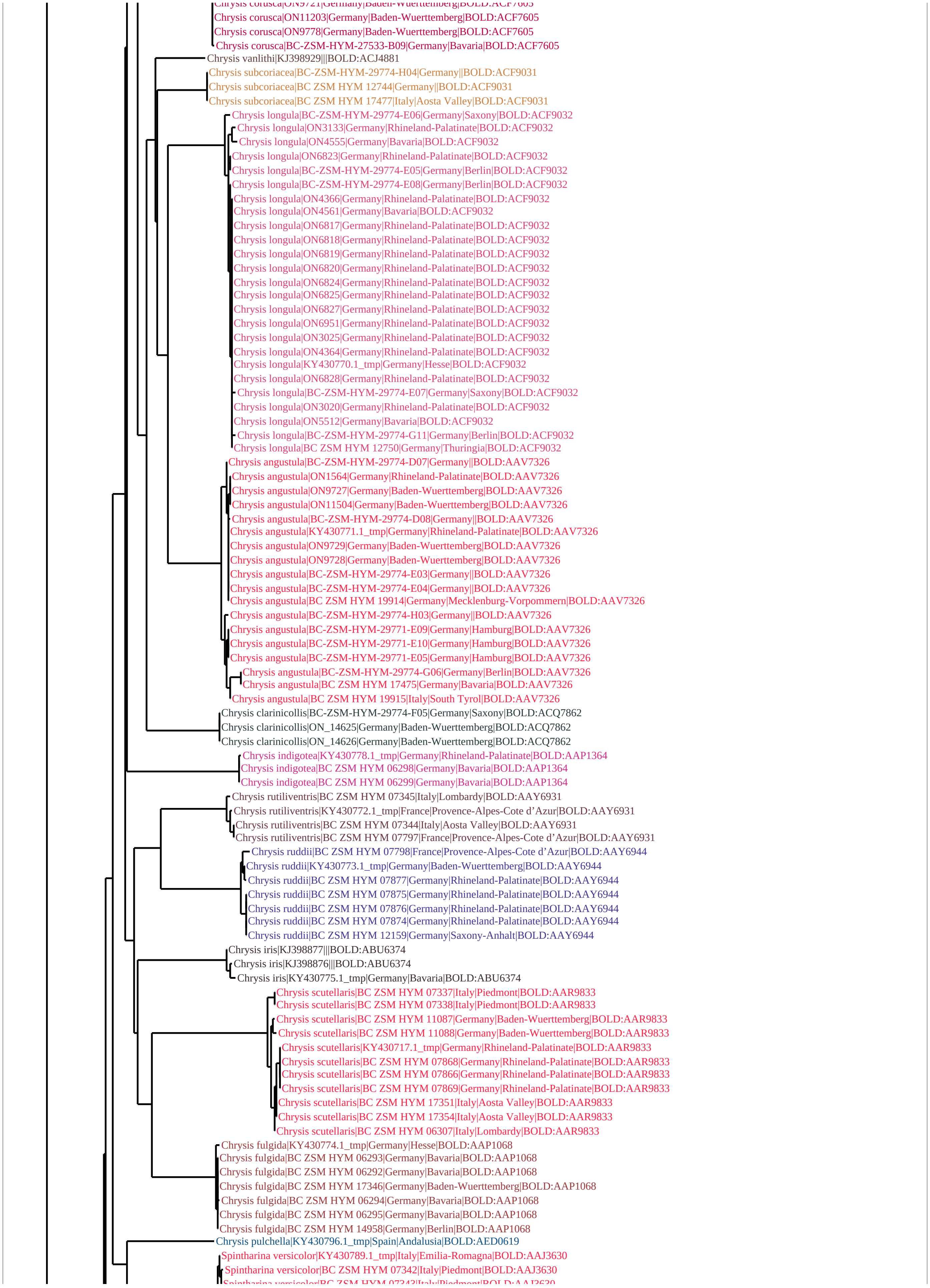
Chrysis cor usca|ON9721|Germany|Baden-Wuerttemberg|BOLD:ACF7605
Chrysis corusca|ON11203|Germany|Baden-Wuerttemberg|BOLD:ACF7605
Chrysis corusca|ON9778|Germany|Baden-Wuerttemberg|BOLD:ACF7605
Chrysis corusca|BC-ZSM-HYM-27533-B09|Germany|Bavaria|BOLD:ACF7605
Chrysis vanlithi|KJ398929|||BOLD:ACJ4881
Chrysis subcoriacea|BC-ZSM-HYM-29774-H04|Germany||BOLD:ACF9031
Chrysis subcoriacea|BC ZSM HYM 12744|Germany||BOLD:ACF9031
Chrysis subcoriacea|BC ZSM HYM 17477|Italy|Aosta Valley|BOLD:ACF9031
Chrysis longula|BC-ZSM-HYM-29774-E06|Germany|Saxony|BOLD:ACF9032
Chrysis longula|ON3133|Germany|Rhineland-Palatinate|BOLD:ACF9032
Chrysis longula|ON4555|Germany|Bavaria|BOLD:ACF9032
Chrysis longula|ON6823|Germany|Rhineland-Palatinate|BOLD:ACF9032
Chrysis longula|BC-ZSM-HYM-29774-E05|Germany|Berlin|BOLD:ACF9032
Chrysis longula|BC-ZSM-HYM-29774-E08|Germany|Berlin|BOLD:ACF9032
Chrysis longula|ON4366|Germany|Rhineland-Palatinate|BOLD:ACF9032
Chrysis longula|ON4561|Germany|Bavaria|BOLD:ACF9032
Chrysis longula|ON6817|Germany|Rhineland-Palatinate|BOLD:ACF9032
Chrysis longula|ON6818|Germany|Rhineland-Palatinate|BOLD:ACF9032
Chrysis longula|ON6819|Germany|Rhineland-Palatinate|BOLD:ACF9032
Chrysis longula|ON6820|Germany|Rhineland-Palatinate|BOLD:ACF9032
Chrysis longula|ON6824|Germany|Rhineland-Palatinate|BOLD:ACF9032
Chrysis longula|ON6825|Germany|Rhineland-Palatinate|BOLD:ACF9032
Chrysis longula|ON6827|Germany|Rhineland-Palatinate|BOLD:ACF9032
Chrysis longula|ON6951|Germany|Rhineland-Palatinate|BOLD:ACF9032
Chrysis longula|ON3025|Germany|Rhineland-Palatinate|BOLD:ACF9032
Chrysis longula|ON4364|Germany|Rhineland-Palatinate|BOLD:ACF9032
Chrysis longula|KY430770.1_tmp|Germany|Hesse|BOLD:ACF9032
Chrysis longula|ON6828|Germany|Rhineland-Palatinate|BOLD:ACF9032
Chrysis longula|BC-ZSM-HYM-29774-E07|Germany|Saxony|BOLD:ACF9032
Chrysis longula|ON3020|Germany|Rhineland-Palatinate|BOLD:ACF9032
Chrysis longula|ON5512|Germany|Bavaria|BOLD:ACF9032
Chrysis longula|BC-ZSM-HYM-29774-G11|Germany|Berlin|BOLD:ACF9032
Chrysis longula|BC ZSM HYM 12750|Germany|Thuringia|BOLD:ACF9032
Chrysis angustula|BC-ZSM-HYM-29774-D07|Germany||BOLD:AAV7326
Chrysis angustula|ON1564|Germany|Rhineland-Palatinate|BOLD:AAV7326
Chrysis angustula|ON9727|Germany|Baden-Wuerttemberg|BOLD:AAV7326
Chrysis angustula|ON11504|Germany|Baden-Wuerttemberg|BOLD:AAV7326
Chrysis angustula|BC-ZSM-HYM-29774-D08|Germany||BOLD:AAV7326
Chrysis angustula|KY430771.1_tmp|Germany|Rhineland-Palatinate|BOLD:AAV7326
Chrysis angustula|ON9729|Germany|Baden-Wuerttemberg|BOLD:AAV7326
Chrysis angustula|ON9728|Germany|Baden-Wuerttemberg|BOLD:AAV7326
Chrysis angustula|BC-ZSM-HYM-29774-E03|Germany||BOLD:AAV7326
Chrysis angustula|BC-ZSM-HYM-29774-E04|Germany||BOLD:AAV7326
Chrysis angustula|BC ZSM HYM 19914|Germany|Mecklenburg-Vorpommern|BOLD:AAV7326
Chrysis angustula|BC-ZSM-HYM-29774-H03|Germany||BOLD:AAV7326
Chrysis angustula|BC-ZSM-HYM-29771-E09|Germany|Hamburg|BOLD:AAV7326
Chrysis angustula|BC-ZSM-HYM-29771-E10|Germany|Hamburg|BOLD:AAV7326
Chrysis angustula|BC-ZSM-HYM-29771-E05|Germany|Hamburg|BOLD:AAV7326
Chrysis angustula|BC-ZSM-HYM-29774-G06|Germany|Berlin|BOLD:AAV7326
Chrysis angustula|BC ZSM HYM 17475|Germany|Bavaria|BOLD:AAV7326
Chrysis angustula|BC ZSM HYM 19915|Italy|South Tyrol|BOLD:AAV7326
Chrysis clarinicollis|BC-ZSM-HYM-29774-F05|Germany|Saxony|BOLD:ACQ7862
Chrysis clarinicollis|ON_14625|Germany|Baden-Wuerttemberg|BOLD:ACQ7862
Chrysis clarinicollis|ON_14626|Germany|Baden-Wuerttemberg|BOLD:ACQ7862
Chrysis indigotea|KY430778.1_tmp|Germany|Rhineland-Palatinate|BOLD:AAP1364
Chrysis indigotea|BC ZSM HYM 06298|Germany|Bavaria|BOLD:AAP1364
Chrysis indigotea|BC ZSM HYM 06299|Germany|Bavaria|BOLD:AAP1364
Chrysis rutiliventris|BC ZSM HYM 07345|Italy|Lombardy|BOLD:AAY6931
Chrysis rutiliventris|KY430772.1_tmp|France|Provence-Alpes-Cote d'Azur|BOLD:AAY6931
Chrysis rutiliventris|BC ZSM HYM 07344|Italy|Aosta Valley|BOLD:AAY6931
Chrysis rutiliventris|BC ZSM HYM 07797|France|Provence-Alpes-Cote d'Azur|BOLD:AAY6931
Chrysis ruddii|BC ZSM HYM 07798|France|Provence-Alpes-Cote d'Azur|BOLD:AAY6944
Chrysis ruddii|KY430773.1_tmp|Germany|Baden-Wuerttemberg|BOLD:AAY6944
Chrysis ruddii|BC ZSM HYM 07877|Germany|Rhineland-Palatinate|BOLD:AAY6944
Chrysis ruddii|BC ZSM HYM 07875|Germany|Rhineland-Palatinate|BOLD:AAY6944
Chrysis ruddii|BC ZSM HYM 07876|Germany|Rhineland-Palatinate|BOLD:AAY6944
Chrysis ruddii|BC ZSM HYM 07874|Germany|Rhineland-Palatinate|BOLD:AAY6944
Chrysis ruddii|BC ZSM HYM 12159|Germany|Saxony-Anhalt|BOLD:AAY6944
Chrysis iris|KJ398877|||BOLD:ABU6374
Chrysis iris|KJ398876|||BOLD:ABU6374
Chrysis iris|KY430775.1_tmp|Germany|Bavaria|BOLD:ABU6374
Chrysis scutellaris|BC ZSM HYM 07337|Italy|Piedmont|BOLD:AAR9833
Chrysis scutellaris|BC ZSM HYM 07338|Italy|Piedmont|BOLD:AAR9833
Chrysis scutellaris|BC ZSM HYM 11087|Germany|Baden-Wuerttemberg|BOLD:AAR9833
Chrysis scutellaris|BC ZSM HYM 11088|Germany|Baden-Wuerttemberg|BOLD:AAR9833
Chrysis scutellaris|KY430717.1_tmp|Germany|Rhineland-Palatinate|BOLD:AAR9833
Chrysis scutellaris|BC ZSM HYM 07868|Germany|Rhineland-Palatinate|BOLD:AAR9833
Chrysis scutellaris|BC ZSM HYM 07866|Germany|Rhineland-Palatinate|BOLD:AAR9833
Chrysis scutellaris|BC ZSM HYM 07869|Germany|Rhineland-Palatinate|BOLD:AAR9833
Chrysis scutellaris|BC ZSM HYM 17351|Italy|Aosta Valley|BOLD:AAR9833
Chrysis scutellaris|BC ZSM HYM 17354|Italy|Aosta Valley|BOLD:AAR9833
Chrysis scutellaris|BC ZSM HYM 06307|Italy|Lombardy|BOLD:AAR9833
Chrysis fulgida|KY430774.1_tmp|Germany|Hesse|BOLD:AAP1068
Chrysis fulgida|BC ZSM HYM 06293|Germany|Bavaria|BOLD:AAP1068
Chrysis fulgida|BC ZSM HYM 06292|Germany|Bavaria|BOLD:AAP1068
Chrysis fulgida|BC ZSM HYM 17346|Germany|Baden-Wuerttemberg|BOLD:AAP1068
Chrysis fulgida|BC ZSM HYM 06294|Germany|Bavaria|BOLD:AAP1068
Chrysis fulgida|BC ZSM HYM 06295|Germany|Bavaria|BOLD:AAP1068
Chrysis fulgida|BC ZSM HYM 14958|Germany|Berlin|BOLD:AAP1068
Chrysis pulchella|KY430796.1_tmp|Spain|Andalusia|BOLD:AED0619
Spintharina versicolor|KY430789.1_tmp|Italy|Emilia-Romagna|BOLD:AAJ3630
Spintharina versicolor|BC ZSM HYM 07342|Italy|Piedmont|BOLD:AAJ3630
Spintharina versicolor|BC ZSM HYM 07343|Italy|Piedmont|BOLD:AAJ3630

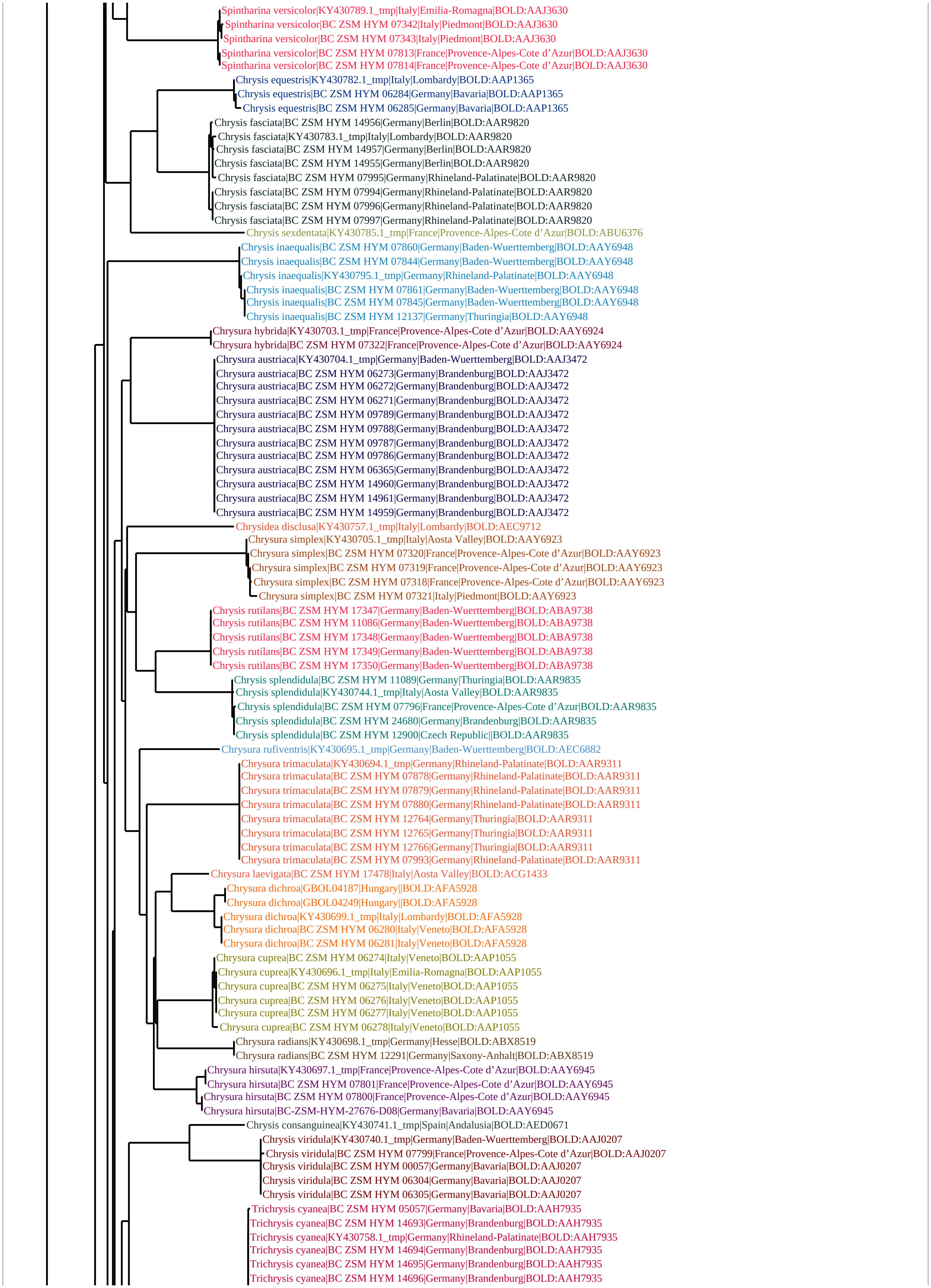
Spintharina versicolor|KY430789.1_tmp|Italy|Emilia-Romagna|BOLD:AAJ3630
Spintharina versicolor|BC ZSM HYM 07342|Italy|Piedmont|BOLD:AAJ3630
Spintharina versicolor|BC ZSM HYM 07343|Italy|Piedmont|BOLD:AAJ3630
Spintharina versicolor|BC ZSM HYM 07813|France|Provence-Alpes-Cote d'Azur|BOLD:AAJ3630
Spintharina versicolor|BC ZSM HYM 07814|France|Provence-Alpes-Cote d'Azur|BOLD:AAJ3630
Chrysis equestris|KY430782.1_tmp|Italy|Lombardy|BOLD:AAP1365
Chrysis equestris|BC ZSM HYM 06284|Germany|Bavaria|BOLD:AAP1365
Chrysis equestris|BC ZSM HYM 06285|Germany|Bavaria|BOLD:AAP1365
Chrysis fasciata|BC ZSM HYM 14956|Germany|Berlin|BOLD:AAR9820
Chrysis fasciata|KY430783.1_tmp|Italy|Lombardy|BOLD:AAR9820
Chrysis fasciata|BC ZSM HYM 14957|Germany|Berlin|BOLD:AAR9820
Chrysis fasciata|BC ZSM HYM 14955|Germany|Berlin|BOLD:AAR9820
Chrysis fasciata|BC ZSM HYM 07995|Germany|Rhineland-Palatinate|BOLD:AAR9820
Chrysis fasciata|BC ZSM HYM 07994|Germany|Rhineland-Palatinate|BOLD:AAR9820
Chrysis fasciata|BC ZSM HYM 07996|Germany|Rhineland-Palatinate|BOLD:AAR9820
Chrysis fasciata|BC ZSM HYM 07997|Germany|Rhineland-Palatinate|BOLD:AAR9820
Chrysis sexdentata|KY430785.1_tmp|France|Provence-Alpes-Cote d'Azur|BOLD:ABU6376
Chrysis inaequalis|BC ZSM HYM 07860|Germany|Baden-Wuerttemberg|BOLD:AAY6948
Chrysis inaequalis|BC ZSM HYM 07844|Germany|Baden-Wuerttemberg|BOLD:AAY6948
Chrysis inaequalis|KY430795.1_tmp|Germany|Rhineland-Palatinate|BOLD:AAY6948
Chrysis inaequalis|BC ZSM HYM 07861|Germany|Baden-Wuerttemberg|BOLD:AAY6948
Chrysis inaequalis|BC ZSM HYM 07845|Germany|Baden-Wuerttemberg|BOLD:AAY6948
Chrysis inaequalis|BC ZSM HYM 12137|Germany|Thuringia|BOLD:AAY6948
Chrysura hybrida|KY430703.1_tmp|France|Provence-Alpes-Cote d'Azur|BOLD:AAY6924
Chrysura hybrida|BC ZSM HYM 07322|France|Provence-Alpes-Cote d'Azur|BOLD:AAY6924
Chrysura austriaca|KY430704.1_tmp|Germany|Baden-Wuerttemberg|BOLD:AAJ3472
Chrysura austriaca|BC ZSM HYM 06273|Germany|Brandenburg|BOLD:AAJ3472
Chrysura austriaca|BC ZSM HYM 06272|Germany|Brandenburg|BOLD:AAJ3472
Chrysura austriaca|BC ZSM HYM 06271|Germany|Brandenburg|BOLD:AAJ3472
Chrysura austriaca|BC ZSM HYM 09789|Germany|Brandenburg|BOLD:AAJ3472
Chrysura austriaca|BC ZSM HYM 09788|Germany|Brandenburg|BOLD:AAJ3472
Chrysura austriaca|BC ZSM HYM 09787|Germany|Brandenburg|BOLD:AAJ3472
Chrysura austriaca|BC ZSM HYM 09786|Germany|Brandenburg|BOLD:AAJ3472
Chrysura austriaca|BC ZSM HYM 06365|Germany|Brandenburg|BOLD:AAJ3472
Chrysura austriaca|BC ZSM HYM 14960|Germany|Brandenburg|BOLD:AAJ3472
Chrysura austriaca|BC ZSM HYM 14961|Germany|Brandenburg|BOLD:AAJ3472
Chrysura austriaca|BC ZSM HYM 14959|Germany|Brandenburg|BOLD:AAJ3472
Chrysidea disclusa|KY430757.1_tmp|Italy|Lombardy|BOLD:AEC9712
Chrysura simplex|KY430705.1_tmp|Italy|Aosta Valley|BOLD:AAY6923
Chrysura simplex|BC ZSM HYM 07320|France|Provence-Alpes-Cote d'Azur|BOLD:AAY6923
Chrysura simplex|BC ZSM HYM 07319|France|Provence-Alpes-Cote d'Azur|BOLD:AAY6923
Chrysura simplex|BC ZSM HYM 07318|France|Provence-Alpes-Cote d'Azur|BOLD:AAY6923
Chrysura simplex|BC ZSM HYM 07321|Italy|Piedmont|BOLD:AAY6923
Chrysis rutilans|BC ZSM HYM 17347|Germany|Baden-Wuerttemberg|BOLD:ABA9738
Chrysis rutilans|BC ZSM HYM 11086|Germany|Baden-Wuerttemberg|BOLD:ABA9738
Chrysis rutilans|BC ZSM HYM 17348|Germany|Baden-Wuerttemberg|BOLD:ABA9738
Chrysis rutilans|BC ZSM HYM 17349|Germany|Baden-Wuerttemberg|BOLD:ABA9738
Chrysis rutilans|BC ZSM HYM 17350|Germany|Baden-Wuerttemberg|BOLD:ABA9738
Chrysis splendidula|BC ZSM HYM 11089|Germany|Thuringia|BOLD:AAR9835
Chrysis splendidula|KY430744.1_tmp|Italy|Aosta Valley|BOLD:AAR9835
Chrysis splendidula|BC ZSM HYM 07796|France|Provence-Alpes-Cote d'Azur|BOLD:AAR9835
Chrysis splendidula|BC ZSM HYM 24680|Germany|Brandenburg|BOLD:AAR9835
Chrysis splendidula|BC ZSM HYM 12900|Czech Republic||BOLD:AAR9835
Chrysura rufiventris|KY430695.1_tmp|Germany|Baden-Wuerttemberg|BOLD:AEC6882
Chrysura trimaculata|KY430694.1_tmp|Germany|Rhineland-Palatinate|BOLD:AAR9311
Chrysura trimaculata|BC ZSM HYM 07878|Germany|Rhineland-Palatinate|BOLD:AAR9311
Chrysura trimaculata|BC ZSM HYM 07879|Germany|Rhineland-Palatinate|BOLD:AAR9311
Chrysura trimaculata|BC ZSM HYM 07880|Germany|Rhineland-Palatinate|BOLD:AAR9311
Chrysura trimaculata|BC ZSM HYM 12764|Germany|Thuringia|BOLD:AAR9311
Chrysura trimaculata|BC ZSM HYM 12765|Germany|Thuringia|BOLD:AAR9311
Chrysura trimaculata|BC ZSM HYM 12766|Germany|Thuringia|BOLD:AAR9311
Chrysura trimaculata|BC ZSM HYM 07993|Germany|Rhineland-Palatinate|BOLD:AAR9311
Chrysura laevigata|BC ZSM HYM 17478|Italy|Aosta Valley|BOLD:ACG1433
Chrysura dichroa|GBOL04187|Hungary||BOLD:AFA5928
Chrysura dichroa|GBOL04249|Hungary||BOLD:AFA5928
Chrysura dichroa|KY430699.1_tmp|Italy|Lombardy|BOLD:AFA5928
Chrysura dichroa|BC ZSM HYM 06280|Italy|Veneto|BOLD:AFA5928
Chrysura dichroa|BC ZSM HYM 06281|Italy|Veneto|BOLD:AFA5928
Chrysura cuprea|BC ZSM HYM 06274|Italy|Veneto|BOLD:AAP1055
Chrysura cuprea|KY430696.1_tmp|Italy|Emilia-Romagna|BOLD:AAP1055
Chrysura cuprea|BC ZSM HYM 06275|Italy|Veneto|BOLD:AAP1055
Chrysura cuprea|BC ZSM HYM 06276|Italy|Veneto|BOLD:AAP1055
Chrysura cuprea|BC ZSM HYM 06277|Italy|Veneto|BOLD:AAP1055
Chrysura cuprea|BC ZSM HYM 06278|Italy|Veneto|BOLD:AAP1055
Chrysura radians|KY430698.1_tmp|Germany|Hesse|BOLD:ABX8519
Chrysura radians|BC ZSM HYM 12291|Germany|Saxony-Anhalt|BOLD:ABX8519
Chrysura hirsuta|KY430697.1_tmp|France|Provence-Alpes-Cote d'Azur|BOLD:AAY6945
Chrysura hirsuta|BC ZSM HYM 07801|France|Provence-Alpes-Cote d'Azur|BOLD:AAY6945
Chrysura hirsuta|BC ZSM HYM 07800|France|Provence-Alpes-Cote d'Azur|BOLD:AAY6945
Chrysura hirsuta|BC-ZSM-HYM-27676-D08|Germany|Bavaria|BOLD:AAY6945
Chrysis consanguinea|KY430741.1_tmp|Spain|Andalusia|BOLD:AED0671
Chrysis viridula|KY430740.1_tmp|Germany|Baden-Wuerttemberg|BOLD:AAJ0207
Chrysis viridula|BC ZSM HYM 07799|France|Provence-Alpes-Cote d'Azur|BOLD:AAJ0207
Chrysis viridula|BC ZSM HYM 00057|Germany|Bavaria|BOLD:AAJ0207
Chrysis viridula|BC ZSM HYM 06304|Germany|Bavaria|BOLD:AAJ0207
Chrysis viridula|BC ZSM HYM 06305|Germany|Bavaria|BOLD:AAJ0207
Trichrysis cyanea|BC ZSM HYM 05057|Germany|Bavaria|BOLD:AAH7935
Trichrysis cyanea|BC ZSM HYM 14693|Germany|Brandenburg|BOLD:AAH7935
Trichrysis cyanea|KY430758.1_tmp|Germany|Rhineland-Palatinate|BOLD:AAH7935
Trichrysis cyanea|BC ZSM HYM 14694|Germany|Brandenburg|BOLD:AAH7935
Trichrysis cyanea|BC ZSM HYM 14695|Germany|Brandenburg|BOLD:AAH7935
Trichrysis cyanea|BC ZSM HYM 14696|Germany|Brandenburg|BOLD:AAH7935

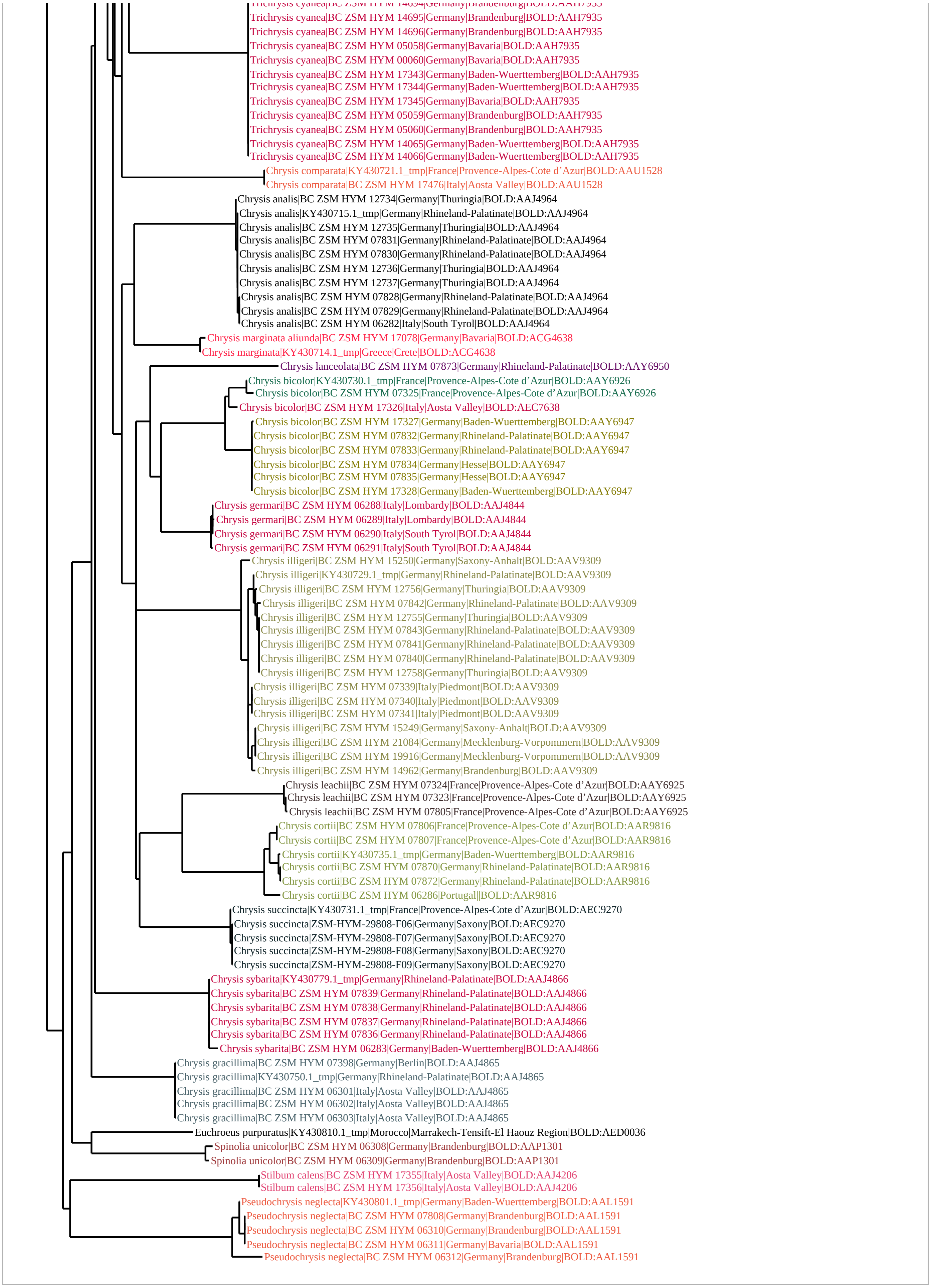
Trichrysis cyanea|BC ZSM HYM 14694|Germany|Brandenburg|BOLD:AAH7935
Trichrysis cyanea|BC ZSM HYM 14695|Germany|Brandenburg|BOLD:AAH7935
Trichrysis cyanea|BC ZSM HYM 14696|Germany|Brandenburg|BOLD:AAH7935
Trichrysis cyanea|BC ZSM HYM 05058|Germany|Bavaria|BOLD:AAH7935
Trichrysis cyanea|BC ZSM HYM 00060|Germany|Bavaria|BOLD:AAH7935
Trichrysis cyanea|BC ZSM HYM 17343|Germany|Baden-Wuerttemberg|BOLD:AAH7935
Trichrysis cyanea|BC ZSM HYM 17344|Germany|Baden-Wuerttemberg|BOLD:AAH7935
Trichrysis cyanea|BC ZSM HYM 17345|Germany|Bavaria|BOLD:AAH7935
Trichrysis cyanea|BC ZSM HYM 05059|Germany|Brandenburg|BOLD:AAH7935
Trichrysis cyanea|BC ZSM HYM 05060|Germany|Brandenburg|BOLD:AAH7935
Trichrysis cyanea|BC ZSM HYM 14065|Germany|Baden-Wuerttemberg|BOLD:AAH7935
Trichrysis cyanea|BC ZSM HYM 14066|Germany|Baden-Wuerttemberg|BOLD:AAH7935
Chrysis comparata|KY430721.1_tmp|France|Provence-Alpes-Cote d'Azur|BOLD:AAU1528
Chrysis comparata|BC ZSM HYM 17476|Italy|Aosta Valley|BOLD:AAU1528
Chrysis analis|BC ZSM HYM 12734|Germany|Thuringia|BOLD:AAJ4964
Chrysis analis|KY430715.1_tmp|Germany|Rhineland-Palatinate|BOLD:AAJ4964
Chrysis analis|BC ZSM HYM 12735|Germany|Thuringia|BOLD:AAJ4964
Chrysis analis|BC ZSM HYM 07831|Germany|Rhineland-Palatinate|BOLD:AAJ4964
Chrysis analis|BC ZSM HYM 07830|Germany|Rhineland-Palatinate|BOLD:AAJ4964
Chrysis analis|BC ZSM HYM 12736|Germany|Thuringia|BOLD:AAJ4964
Chrysis analis|BC ZSM HYM 12737|Germany|Thuringia|BOLD:AAJ4964
Chrysis analis|BC ZSM HYM 07828|Germany|Rhineland-Palatinate|BOLD:AAJ4964
Chrysis analis|BC ZSM HYM 07829|Germany|Rhineland-Palatinate|BOLD:AAJ4964
Chrysis analis|BC ZSM HYM 06282|Italy|South Tyrol|BOLD:AAJ4964
Chrysis marginata aliunda|BC ZSM HYM 17078|Germany|Bavaria|BOLD:ACG4638
Chrysis marginata|KY430714.1_tmp|Greece|Crete|BOLD:ACG4638
Chrysis lanceolata|BC ZSM HYM 07873|Germany|Rhineland-Palatinate|BOLD:AAY6950
Chrysis bicolor|KY430730.1_tmp|France|Provence-Alpes-Cote d'Azur|BOLD:AAY6926
Chrysis bicolor|BC ZSM HYM 07325|France|Provence-Alpes-Cote d'Azur|BOLD:AAY6926
Chrysis bicolor|BC ZSM HYM 17326|Italy|Aosta Valley|BOLD:AEC7638
Chrysis bicolor|BC ZSM HYM 17327|Germany|Baden-Wuerttemberg|BOLD:AAY6947
Chrysis bicolor|BC ZSM HYM 07832|Germany|Rhineland-Palatinate|BOLD:AAY6947
Chrysis bicolor|BC ZSM HYM 07833|Germany|Rhineland-Palatinate|BOLD:AAY6947
Chrysis bicolor|BC ZSM HYM 07834|Germany|Hesse|BOLD:AAY6947
Chrysis bicolor|BC ZSM HYM 07835|Germany|Hesse|BOLD:AAY6947
Chrysis bicolor|BC ZSM HYM 17328|Germany|Baden-Wuerttemberg|BOLD:AAY6947
Chrysis germari|BC ZSM HYM 06288|Italy|Lombardy|BOLD:AAJ4844
Chrysis germari|BC ZSM HYM 06289|Italy|Lombardy|BOLD:AAJ4844
Chrysis germari|BC ZSM HYM 06290|Italy|South Tyrol|BOLD:AAJ4844
Chrysis germari|BC ZSM HYM 06291|Italy|South Tyrol|BOLD:AAJ4844
Chrysis illigeri|BC ZSM HYM 15250|Germany|Saxony-Anhalt|BOLD:AAV9309
Chrysis illigeri|KY430729.1_tmp|Germany|Rhineland-Palatinate|BOLD:AAV9309
Chrysis illigeri|BC ZSM HYM 12756|Germany|Thuringia|BOLD:AAV9309
Chrysis illigeri|BC ZSM HYM 07842|Germany|Rhineland-Palatinate|BOLD:AAV9309
Chrysis illigeri|BC ZSM HYM 12755|Germany|Thuringia|BOLD:AAV9309
Chrysis illigeri|BC ZSM HYM 07843|Germany|Rhineland-Palatinate|BOLD:AAV9309
Chrysis illigeri|BC ZSM HYM 07841|Germany|Rhineland-Palatinate|BOLD:AAV9309
Chrysis illigeri|BC ZSM HYM 07840|Germany|Rhineland-Palatinate|BOLD:AAV9309
Chrysis illigeri|BC ZSM HYM 12758|Germany|Thuringia|BOLD:AAV9309
Chrysis illigeri|BC ZSM HYM 07339|Italy|Piedmont|BOLD:AAV9309
Chrysis illigeri|BC ZSM HYM 07340|Italy|Piedmont|BOLD:AAV9309
Chrysis illigeri|BC ZSM HYM 07341|Italy|Piedmont|BOLD:AAV9309
Chrysis illigeri|BC ZSM HYM 15249|Germany|Saxony-Anhalt|BOLD:AAV9309
Chrysis illigeri|BC ZSM HYM 21084|Germany|Mecklenburg-Vorpommern|BOLD:AAV9309
Chrysis illigeri|BC ZSM HYM 19916|Germany|Mecklenburg-Vorpommern|BOLD:AAV9309
Chrysis illigeri|BC ZSM HYM 14962|Germany|Brandenburg|BOLD:AAV9309
Chrysis leachii|BC ZSM HYM 07324|France|Provence-Alpes-Cote d'Azur|BOLD:AAY6925
Chrysis leachii|BC ZSM HYM 07323|France|Provence-Alpes-Cote d'Azur|BOLD:AAY6925
Chrysis leachii|BC ZSM HYM 07805|France|Provence-Alpes-Cote d'Azur|BOLD:AAY6925
Chrysis cortii|BC ZSM HYM 07806|France|Provence-Alpes-Cote d'Azur|BOLD:AAR9816
Chrysis cortii|BC ZSM HYM 07807|France|Provence-Alpes-Cote d'Azur|BOLD:AAR9816
Chrysis cortii|KY430735.1_tmp|Germany|Baden-Wuerttemberg|BOLD:AAR9816
Chrysis cortii|BC ZSM HYM 07870|Germany|Rhineland-Palatinate|BOLD:AAR9816
Chrysis cortii|BC ZSM HYM 07872|Germany|Rhineland-Palatinate|BOLD:AAR9816
Chrysis cortii|BC ZSM HYM 06286|Portugal||BOLD:AAR9816
Chrysis succincta|KY430731.1_tmp|France|Provence-Alpes-Cote d'Azur|BOLD:AEC9270
Chrysis succincta|ZSM-HYM-29808-F06|Germany|Saxony|BOLD:AEC9270
Chrysis succincta|ZSM-HYM-29808-F07|Germany|Saxony|BOLD:AEC9270
Chrysis succincta|ZSM-HYM-29808-F08|Germany|Saxony|BOLD:AEC9270
Chrysis succincta|ZSM-HYM-29808-F09|Germany|Saxony|BOLD:AEC9270
Chrysis sybarita|KY430779.1_tmp|Germany|Rhineland-Palatinate|BOLD:AAJ4866
Chrysis sybarita|BC ZSM HYM 07839|Germany|Rhineland-Palatinate|BOLD:AAJ4866
Chrysis sybarita|BC ZSM HYM 07838|Germany|Rhineland-Palatinate|BOLD:AAJ4866
Chrysis sybarita|BC ZSM HYM 07837|Germany|Rhineland-Palatinate|BOLD:AAJ4866
Chrysis sybarita|BC ZSM HYM 07836|Germany|Rhineland-Palatinate|BOLD:AAJ4866
Chrysis sybarita|BC ZSM HYM 06283|Germany|Baden-Wuerttemberg|BOLD:AAJ4866
Chrysis gracillima|BC ZSM HYM 07398|Germany|Berlin|BOLD:AAJ4865
Chrysis gracillima|KY430750.1_tmp|Germany|Rhineland-Palatinate|BOLD:AAJ4865
Chrysis gracillima|BC ZSM HYM 06301|Italy|Aosta Valley|BOLD:AAJ4865
Chrysis gracillima|BC ZSM HYM 06302|Italy|Aosta Valley|BOLD:AAJ4865
Chrysis gracillima|BC ZSM HYM 06303|Italy|Aosta Valley|BOLD:AAJ4865
Euchroeus purpuratus|KY430810.1_tmp|Morocco|Marrakech-Tensift-El Haouz Region|BOLD:AED0036
Spinolia unicolor|BC ZSM HYM 06308|Germany|Brandenburg|BOLD:AAP1301
Spinolia unicolor|BC ZSM HYM 06309|Germany|Brandenburg|BOLD:AAP1301
Stilbum calens|BC ZSM HYM 17355|Italy|Aosta Valley|BOLD:AAJ4206
Stilbum calens|BC ZSM HYM 17356|Italy|Aosta Valley|BOLD:AAJ4206
Pseudochrysis neglecta|KY430801.1_tmp|Germany|Baden-Wuerttemberg|BOLD:AAL1591
Pseudochrysis neglecta|BC ZSM HYM 07808|Germany|Brandenburg|BOLD:AAL1591
Pseudochrysis neglecta|BC ZSM HYM 06310|Germany|Brandenburg|BOLD:AAL1591
Pseudochrysis neglecta|BC ZSM HYM 06311|Germany|Bavaria|BOLD:AAL1591
Pseudochrysis neglecta|BC ZSM HYM 06312|Germany|Brandenburg|BOLD:AAL1591
