# Supplementary material for: DNA Barcoding of German Cuckoo Wasps (Hymenoptera: Chrysididae) Suggests Cryptic Species in Several Widely Distributed Species
Source: Insects. 2024 Oct 30;15(11):850. doi: 10.3390/insects15110850 (PMC11594385; doi:10.3390/insects15110850)
Supplement: Supplementary file 1 [file insects-15-00850-s001.zip › Supplementary materials/Supplement 2 Chrysididae vouchers.pdf]

Supplement 2. List of voucher specimens with specimen ID, country of origin, collection date, specimen depository, Barcode Index Number (BIN) and sequencing success (COI-fragment length in bp, in square brackets number of unresolved bases).

Species	Specimen ID	Country	Collection Date	Depository	BIN	COI-5P
**Chrysellampus sculpticollis**	ON_14410	Italy	16-Jun-2019	Private Collection of Oliver Niehuis	BOLD:AEJ0421	675[0n]
**Chrysidea disclusa**	KY430757.1_tmp	Italy	21-May-2011	Private Collection of Oliver Niehuis	BOLD:AEC9712	675[0n]
**Chrysis analis**	KY430715.1_tmp	Germany	26-Jun-2010	Private Collection of Oliver Niehuis	BOLD:AAJ4964	675[0n]
**Chrysis analis**	BC ZSM HYM 07828	Germany	06-Jul-2010	SNSB, Zoologische Staatssammlung Muenchen	BOLD:AAJ4964	658[0n]
**Chrysis analis**	BC ZSM HYM 07829	Germany	03-Jul-2010	SNSB, Zoologische Staatssammlung Muenchen	BOLD:AAJ4964	658[0n]
**Chrysis analis**	BC ZSM HYM 07830	Germany	24-Jun-2009	SNSB, Zoologische Staatssammlung Muenchen	BOLD:AAJ4964	658[0n]
**Chrysis analis**	BC ZSM HYM 07831	Germany	24-Jun-2009	SNSB, Zoologische Staatssammlung Muenchen	BOLD:AAJ4964	658[0n]
**Chrysis analis**	BC ZSM HYM 12734	Germany	04-Jul-2011	SNSB, Zoologische Staatssammlung Muenchen	BOLD:AAJ4964	658[0n]
**Chrysis analis**	BC ZSM HYM 12735	Germany	04-Jul-2011	SNSB, Zoologische Staatssammlung Muenchen	BOLD:AAJ4964	658[0n]
**Chrysis analis**	BC ZSM HYM 12736	Germany	04-Jun-2011	SNSB, Zoologische Staatssammlung Muenchen	BOLD:AAJ4964	629[0n]
**Chrysis analis**	BC ZSM HYM 12737	Germany	25-Jun-2011	SNSB, Zoologische Staatssammlung Muenchen	BOLD:AAJ4964	658[0n]
**Chrysis analis**	BC ZSM HYM 06282	Italy	08-Jun-2007	SNSB, Zoologische Staatssammlung Muenchen	BOLD:AAJ4964	658[0n]
**Chrysis angustula**	ON1564	Germany	05-Jun-2011	Research Collection of Oliver Niehuis	BOLD:AAV7326	676[0n]
**Chrysis angustula**	ON9728	Germany	15-May-2017	Research Collection of Oliver Niehuis	BOLD:AAV7326	665[0n]
**Chrysis angustula**	ON9729	Germany	15-May-2017	Research Collection of Oliver Niehuis	BOLD:AAV7326	664[0n]
**Chrysis angustula**	ON9727	Germany	15-May-2017	Research Collection of Oliver Niehuis	BOLD:AAV7326	676[0n]
**Chrysis angustula**	ON11504	Germany	15-May-2017	Research Collection of Oliver Niehuis	BOLD:AAV7326	676[0n]
**Chrysis angustula**	BC-ZSM-HYM-29774-D07	Germany	15-Jun-2018	SNSB, Zoologische Staatssammlung Muenchen	BOLD:AAV7326	623[0n]
**Chrysis angustula**	BC-ZSM-HYM-29774-D08	Germany	15-Jun-2018	SNSB, Zoologische Staatssammlung Muenchen	BOLD:AAV7326	627[0n]
**Chrysis angustula**	BC-ZSM-HYM-29774-E03	Germany	15-Jul-2018	SNSB, Zoologische Staatssammlung Muenchen	BOLD:AAV7326	632[0n]
**Chrysis angustula**	BC-ZSM-HYM-	Germany	15-Jul-2018	SNSB, Zoologische	BOLD:AAV7326	632[0n]

	29774-E04			Staatssammlung Muenchen		
**Chrysis angustula**	BC-ZSM-HYM-29774-G06	Germany	12-May-2009	SNSB, Zoologische Staatssammlung Muenchen	BOLD:AAV7326	421[0n]
**Chrysis angustula**	BC-ZSM-HYM-29774-H03	Germany	14-Aug-2009	SNSB, Zoologische Staatssammlung Muenchen	BOLD:AAV7326	634[0n]
**Chrysis angustula**	KY430771.1_tmp	Germany	22-Jun-2012	Private Collection of Oliver Niehuis	BOLD:AAV7326	675[0n]
**Chrysis angustula**	BC-ZSM-HYM-29771-E09	Germany	21-Jun-2016	SNSB, Zoologische Staatssammlung Muenchen	BOLD:AAV7326	658[0n]
**Chrysis angustula**	BC-ZSM-HYM-29771-E10	Germany	09-Sep-2016	SNSB, Zoologische Staatssammlung Muenchen	BOLD:AAV7326	629[0n]
**Chrysis angustula**	BC-ZSM-HYM-29771-E05	Germany	22-Jun-2017	SNSB, Zoologische Staatssammlung Muenchen	BOLD:AAV7326	631[0n]
**Chrysis angustula**	BC ZSM HYM 17475	Germany	24-May-2008	SNSB, Zoologische Staatssammlung Muenchen	BOLD:AAV7326	658[0n]
**Chrysis angustula**	BC ZSM HYM 19914	Germany	09-Jun-2013	SNSB, Zoologische Staatssammlung Muenchen	BOLD:AAV7326	658[0n]
**Chrysis angustula**	BC ZSM HYM 19915	Italy	31-Jul-2013	SNSB, Zoologische Staatssammlung Muenchen	BOLD:AAV7326	658[0n]
**Chrysis bicolor**	KY430730.1_tmp	France	23-Jul-1998	Private Collection of Oliver Niehuis	BOLD:AAY6926	675[0n]
**Chrysis bicolor**	BC ZSM HYM 07325	France	12-Jul-2009	SNSB, Zoologische Staatssammlung Muenchen	BOLD:AAY6926	658[0n]
**Chrysis bicolor**	BC ZSM HYM 07832	Germany	26-Jun-2008	SNSB, Zoologische Staatssammlung Muenchen	BOLD:AAY6947	658[0n]
**Chrysis bicolor**	BC ZSM HYM 07833	Germany	26-Jun-2008	SNSB, Zoologische Staatssammlung Muenchen	BOLD:AAY6947	658[0n]
**Chrysis bicolor**	BC ZSM HYM 07834	Germany	07-Jun-2007	SNSB, Zoologische Staatssammlung Muenchen	BOLD:AAY6947	658[0n]
**Chrysis bicolor**	BC ZSM HYM 07835	Germany	19-May-2008	SNSB, Zoologische Staatssammlung Muenchen	BOLD:AAY6947	658[0n]
**Chrysis bicolor**	BC ZSM HYM 17326	Italy	20-Jul-2012	SNSB, Zoologische Staatssammlung Muenchen	BOLD:AEC7638	658[0n]
**Chrysis bicolor**	BC ZSM HYM 17327	Germany	18-Jul-2012	SNSB, Zoologische Staatssammlung Muenchen	BOLD:AAY6947	615[0n]
**Chrysis bicolor**	BC ZSM HYM 17328	Germany	18-Jul-2012	SNSB, Zoologische Staatssammlung Muenchen	BOLD:AAY6947	658[0n]
**Chrysis brevitarsis**	KU854911	Lithuania		Mined from GenBank, NCBI	BOLD:ACG7211	1545[0n]
**Chrysis brevitarsis**	JX292241	Sweden		Mined from GenBank, NCBI	BOLD:ACG7211	765[0n]
**Chrysis clarinicollis**	BC-ZSM-HYM-29774-F05	Germany	18-Aug-2011	SNSB, Zoologische Staatssammlung Muenchen	BOLD:ACQ7862	603[0n]
**Chrysis clarinicollis**	ON_14625	Germany	06-Aug-2021	Private Collection of Oliver Niehuis	BOLD:ACQ7862	658[0n]
**Chrysis clarinicollis**	ON_14626	Germany	06-Aug-2021	Private Collection of Oliver Niehuis	BOLD:ACQ7862	658[0n]
**Chrysis comparata**	KY430721.1_tmp	France	14-Jul-2011	Private Collection of Oliver Niehuis	BOLD:AAU1528	675[0n]

**Chrysis comparata**	BC ZSM HYM 17476	Italy	20-Jul-2012	SNSB, Zoologische Staatssammlung Muenchen	BOLD:AAU1528	658[0n]
**Chrysis consanguinea**	KY430741.1_tmp	Spain	30-May-2011	Private Collection of Oliver Niehuis	BOLD:AED0671	675[0n]
**Chrysis cortii**	KY430735.1_tmp	Germany	24-May-2011	Private Collection of Oliver Niehuis	BOLD:AAR9816	675[0n]
**Chrysis cortii**	BC ZSM HYM 07806	France	10-Jul-2010	SNSB, Zoologische Staatssammlung Muenchen	BOLD:AAR9816	658[0n]
**Chrysis cortii**	BC ZSM HYM 07807	France	10-Jul-2010	SNSB, Zoologische Staatssammlung Muenchen	BOLD:AAR9816	658[0n]
**Chrysis cortii**	BC ZSM HYM 07870	Germany	07-Jul-2002	SNSB, Zoologische Staatssammlung Muenchen	BOLD:AAR9816	658[0n]
**Chrysis cortii**	BC ZSM HYM 07872	Germany	02-Jul-2006	SNSB, Zoologische Staatssammlung Muenchen	BOLD:AAR9816	658[0n]
**Chrysis cortii**	BC ZSM HYM 06286	Portugal	08-Aug-2001	SNSB, Zoologische Staatssammlung Muenchen	BOLD:AAR9816	658[0n]
**Chrysis corusca**	ON1700	Germany	18-Jul-2010	Research Collection of Oliver Niehuis	BOLD:ACF7605	676[1n]
**Chrysis corusca**	ON5232	Sweden	12-Jun-2013	Research Collection of Oliver Niehuis	BOLD:ACF7605	676[0n]
**Chrysis corusca**	ON9734	Germany	15-May-2017	Research Collection of Oliver Niehuis	BOLD:ACF7605	676[0n]
**Chrysis corusca**	ON9710	Germany	15-May-2017	Research Collection of Oliver Niehuis	BOLD:ACF7605	676[0n]
**Chrysis corusca**	ON9735	Germany	15-May-2017	Research Collection of Oliver Niehuis	BOLD:ACF7605	676[0n]
**Chrysis corusca**	ON11421	Germany	15-May-2017	Research Collection of Oliver Niehuis	BOLD:ACF7605	668[0n]
**Chrysis corusca**	ON9724	Germany	15-May-2017	Research Collection of Oliver Niehuis	BOLD:ACF7605	676[0n]
**Chrysis corusca**	ON9740	Germany	15-May-2017	Research Collection of Oliver Niehuis	BOLD:ACF7605	676[0n]
**Chrysis corusca**	ON9731	Germany	15-May-2017	Research Collection of Oliver Niehuis	BOLD:ACF7605	676[0n]
**Chrysis corusca**	ON9733	Germany	15-May-2017	Research Collection of Oliver Niehuis	BOLD:ACF7605	676[0n]
**Chrysis corusca**	ON9721	Germany	15-May-2017	Research Collection of Oliver Niehuis	BOLD:ACF7605	664[0n]
**Chrysis corusca**	ON9781	Germany	15-May-2017	Research Collection of Oliver Niehuis	BOLD:ACF7605	676[0n]
**Chrysis corusca**	ON9720	Germany	15-May-2017	Research Collection of Oliver Niehuis	BOLD:ACF7605	676[0n]
**Chrysis corusca**	ON9739	Germany	15-May-2017	Research Collection of Oliver Niehuis	BOLD:ACF7605	676[0n]
**Chrysis corusca**	ON9736	Germany	15-May-2017	Research Collection of Oliver Niehuis	BOLD:ACF7605	676[0n]

**Chrysis corusca**	ON11188	Germany	15-May-2017	Research Collection of Oliver Niehuis	BOLD:ACF7605	676[0n]
**Chrysis corusca**	ON11114	Germany	15-May-2017	Research Collection of Oliver Niehuis	BOLD:ACF7605	676[0n]
**Chrysis corusca**	ON11192	Germany	15-May-2017	Research Collection of Oliver Niehuis	BOLD:ACF7605	676[0n]
**Chrysis corusca**	ON11198	Germany	15-May-2017	Research Collection of Oliver Niehuis	BOLD:ACF7605	676[0n]
**Chrysis corusca**	ON11194	Germany	15-May-2017	Research Collection of Oliver Niehuis	BOLD:ACF7605	676[0n]
**Chrysis corusca**	ON11199	Germany	15-May-2017	Research Collection of Oliver Niehuis	BOLD:ACF7605	676[0n]
**Chrysis corusca**	ON11202	Germany	15-May-2017	Research Collection of Oliver Niehuis	BOLD:ACF7605	676[0n]
**Chrysis corusca**	ON11204	Germany	15-May-2017	Research Collection of Oliver Niehuis	BOLD:ACF7605	676[0n]
**Chrysis corusca**	ON11205	Germany	15-May-2017	Research Collection of Oliver Niehuis	BOLD:ACF7605	676[0n]
**Chrysis corusca**	ON11206	Germany	15-May-2017	Research Collection of Oliver Niehuis	BOLD:ACF7605	676[0n]
**Chrysis corusca**	ON11203	Germany	15-May-2017	Research Collection of Oliver Niehuis	BOLD:ACF7605	655[0n]
**Chrysis corusca**	ON11210	Germany	15-May-2017	Research Collection of Oliver Niehuis	BOLD:ACF7605	676[0n]
**Chrysis corusca**	ON9778	Germany	15-May-2017	Research Collection of Oliver Niehuis	BOLD:ACF7605	652[0n]
**Chrysis corusca**	ON9793	Germany	15-May-2017	Research Collection of Oliver Niehuis	BOLD:ACF7605	676[0n]
**Chrysis corusca**	ON9792	Germany	15-May-2017	Research Collection of Oliver Niehuis	BOLD:ACF7605	676[0n]
**Chrysis corusca**	ON9786	Germany	15-May-2017	Research Collection of Oliver Niehuis	BOLD:ACF7605	676[0n]
**Chrysis corusca**	ON10493	Germany	15-May-2017	Research Collection of Oliver Niehuis	BOLD:ACF7605	676[0n]
**Chrysis corusca**	ON10497	Germany	15-May-2017	Research Collection of Oliver Niehuis	BOLD:ACF7605	676[0n]
**Chrysis corusca**	ON9785	Germany	15-May-2017	Research Collection of Oliver Niehuis	BOLD:ACF7605	676[0n]
**Chrysis corusca**	ON10538	Germany	15-May-2017	Research Collection of Oliver Niehuis	BOLD:ACF7605	676[0n]
**Chrysis corusca**	ON10547	Germany	15-May-2017	Research Collection of Oliver Niehuis	BOLD:ACF7605	676[0n]
**Chrysis corusca**	ON10534	Germany	15-May-2017	Research Collection of Oliver Niehuis	BOLD:ACF7605	676[0n]
**Chrysis corusca**	ON11084	Germany	15-May-2017	Research Collection of Oliver Niehuis	BOLD:ACF7605	676[0n]
**Chrysis corusca**	ON11111	Germany	15-May-2017	Research Collection of Oliver Niehuis	BOLD:ACF7605	676[0n]

**Chrysis corusca**	ON11075	Germany	15-May-2017	Research Collection of Oliver Niehuis	BOLD:ACF7605	676[0n]
**Chrysis corusca**	ON9779	Germany	15-May-2017	Research Collection of Oliver Niehuis	BOLD:ACF7605	676[1n]
**Chrysis corusca**	ON11466	Germany	15-May-2017	Research Collection of Oliver Niehuis	BOLD:ACF7605	676[0n]
**Chrysis corusca**	ON11477	Germany	15-May-2017	Research Collection of Oliver Niehuis	BOLD:ACF7605	676[0n]
**Chrysis corusca**	ON11480	Germany	15-May-2017	Research Collection of Oliver Niehuis	BOLD:ACF7605	657[0n]
**Chrysis corusca**	ON11506	Germany	15-May-2017	Research Collection of Oliver Niehuis	BOLD:ACF7605	676[0n]
**Chrysis corusca**	ON11508	Germany	15-May-2017	Research Collection of Oliver Niehuis	BOLD:ACF7605	676[0n]
**Chrysis corusca**	ON11518	Germany	15-May-2017	Research Collection of Oliver Niehuis	BOLD:ACF7605	676[0n]
**Chrysis corusca**	ON11532	Germany	15-May-2017	Research Collection of Oliver Niehuis	BOLD:ACF7605	676[0n]
**Chrysis corusca**	BC-ZSM-HYM-29774-G01	Germany	23-May-2009	SNSB, Zoologische Staatssammlung Muenchen	BOLD:ACF7605	633[0n]
**Chrysis corusca**	BC-ZSM-HYM-29774-G02	Germany	24-Jun-2009	SNSB, Zoologische Staatssammlung Muenchen	BOLD:ACF7605	634[0n]
**Chrysis corusca**	KY430769.1_tmp	Germany	24-Jul-2012	Private Collection of Oliver Niehuis	BOLD:ACF7605	675[0n]
**Chrysis corusca**	BC-ZSM-HYM-29771-D12	Germany	30-Jun-2015	SNSB, Zoologische Staatssammlung Muenchen	BOLD:ACF7605	658[0n]
**Chrysis corusca**	BC-ZSM-HYM-27533-B09	Germany	30-May-2014	SNSB, Zoologische Staatssammlung Muenchen	BOLD:ACF7605	658[0n]
**Chrysis equestris**	KY430782.1_tmp	Italy	21-May-2011	Private Collection of Oliver Niehuis	BOLD:AAP1365	675[0n]
**Chrysis equestris**	BC ZSM HYM 06284	Germany	02-Jul-2008	SNSB, Zoologische Staatssammlung Muenchen	BOLD:AAP1365	658[0n]
**Chrysis equestris**	BC ZSM HYM 06285	Germany	01-Jul-2008	SNSB, Zoologische Staatssammlung Muenchen	BOLD:AAP1365	651[0n]
**Chrysis fasciata**	KY430783.1_tmp	Italy	21-May-2011	Private Collection of Oliver Niehuis	BOLD:AAR9820	675[0n]
**Chrysis fasciata**	BC ZSM HYM 14955	Germany	30-Jun-2012	SNSB, Zoologische Staatssammlung Muenchen	BOLD:AAR9820	658[0n]
**Chrysis fasciata**	BC ZSM HYM 14957	Germany	30-Jun-2012	SNSB, Zoologische Staatssammlung Muenchen	BOLD:AAR9820	658[0n]
**Chrysis fasciata**	BC ZSM HYM 14956	Germany	30-Jun-2012	SNSB, Zoologische Staatssammlung Muenchen	BOLD:AAR9820	658[0n]
**Chrysis fasciata**	BC ZSM HYM 07994	Germany	05-Jul-2008	SNSB, Zoologische Staatssammlung Muenchen	BOLD:AAR9820	658[0n]
**Chrysis fasciata**	BC ZSM HYM 07995	Germany	20-Jun-2008	SNSB, Zoologische Staatssammlung Muenchen	BOLD:AAR9820	658[0n]

**Chrysis fasciata**	BC ZSM HYM 07996	Germany	28-Mar-2005	SNSB, Zoologische Staatssammlung Muenchen	BOLD:AAR9820	658[0n]
**Chrysis fasciata**	BC ZSM HYM 07997	Germany	19-Jun-2005	SNSB, Zoologische Staatssammlung Muenchen	BOLD:AAR9820	658[0n]
**Chrysis fulgida**	KY430774.1_tmp	Germany	04-Jun-1998	Private Collection of Oliver Niehuis	BOLD:AAP1068	675[0n]
**Chrysis fulgida**	BC ZSM HYM 17346	Germany	18-Jul-2012	SNSB, Zoologische Staatssammlung Muenchen	BOLD:AAP1068	658[0n]
**Chrysis fulgida**	BC ZSM HYM 06292	Germany	14-Aug-2008	SNSB, Zoologische Staatssammlung Muenchen	BOLD:AAP1068	658[0n]
**Chrysis fulgida**	BC ZSM HYM 06293	Germany	01-Aug-2008	SNSB, Zoologische Staatssammlung Muenchen	BOLD:AAP1068	658[0n]
**Chrysis fulgida**	BC ZSM HYM 06294	Germany	01-Aug-2008	SNSB, Zoologische Staatssammlung Muenchen	BOLD:AAP1068	658[0n]
**Chrysis fulgida**	BC ZSM HYM 06295	Germany	01-Jul-2008	SNSB, Zoologische Staatssammlung Muenchen	BOLD:AAP1068	658[0n]
**Chrysis fulgida**	BC ZSM HYM 14958	Germany	30-Jun-2012	SNSB, Zoologische Staatssammlung Muenchen	BOLD:AAP1068	658[0n]
**Chrysis germari**	BC ZSM HYM 06288	Italy	09-Jul-2006	SNSB, Zoologische Staatssammlung Muenchen	BOLD:AAJ4844	613[0n]
**Chrysis germari**	BC ZSM HYM 06289	Italy	09-Jul-2006	SNSB, Zoologische Staatssammlung Muenchen	BOLD:AAJ4844	646[0n]
**Chrysis germari**	BC ZSM HYM 06290	Italy	08-Jun-2007	SNSB, Zoologische Staatssammlung Muenchen	BOLD:AAJ4844	658[0n]
**Chrysis germari**	BC ZSM HYM 06291	Italy	08-Jun-2007	SNSB, Zoologische Staatssammlung Muenchen	BOLD:AAJ4844	658[0n]
**Chrysis gracillima**	KY430750.1_tmp	Germany	21-May-2011	Private Collection of Oliver Niehuis	BOLD:AAJ4865	675[0n]
**Chrysis gracillima**	BC ZSM HYM 07398	Germany	26-Jul-2010	SNSB, Zoologische Staatssammlung Muenchen	BOLD:AAJ4865	658[0n]
**Chrysis gracillima**	BC ZSM HYM 06301	Italy	25-Jul-1999	SNSB, Zoologische Staatssammlung Muenchen	BOLD:AAJ4865	658[0n]
**Chrysis gracillima**	BC ZSM HYM 06302	Italy	27-Jun-1999	SNSB, Zoologische Staatssammlung Muenchen	BOLD:AAJ4865	658[0n]
**Chrysis gracillima**	BC ZSM HYM 06303	Italy	06-Jul-1995	SNSB, Zoologische Staatssammlung Muenchen	BOLD:AAJ4865	658[0n]
**Chrysis horridula**	ON1257	France	14-Jul-2011	Research Collection of Oliver Niehuis	BOLD:AAU2328	676[0n]
**Chrysis horridula**	BC-ZSM-HYM-29774-F06	Germany	06-Aug-2008	SNSB, Zoologische Staatssammlung Muenchen	BOLD:AAU2328	632[0n]
**Chrysis horridula**	BC-ZSM-HYM-29774-F08	Germany	12-Jun-2013	SNSB, Zoologische Staatssammlung Muenchen	BOLD:AAU2328	631[1n]
**Chrysis horridula**	BC-ZSM-HYM-29774-F09	Germany	18-Jun-2014	SNSB, Zoologische Staatssammlung Muenchen	BOLD:AAU2328	632[1n]
**Chrysis horridula**	BC-ZSM-HYM-29774-G08	Germany	21-Jul-2010	SNSB, Zoologische Staatssammlung Muenchen	BOLD:AAU2328	638[1n]
**Chrysis horridula**	BC-ZSM-HYM-29774-G09	Germany	15-Aug-2012	SNSB, Zoologische Staatssammlung Muenchen	BOLD:AAU2328	632[1n]

**Chrysis horridula**	BC ZSM HYM 15251	Germany	04-Aug-2012	SNSB, Zoologische Staatssammlung Muenchen	BOLD:AAU2328	619[0n]
**Chrysis horridula**	BC-ZSM-HYM-29771-E01	Germany	27-May-2017	SNSB, Zoologische Staatssammlung Muenchen	BOLD:AAU2328	640[0n]
**Chrysis horridula**	BC-ZSM-HYM-29771-E06	Germany	30-Jul-2017	SNSB, Zoologische Staatssammlung Muenchen	BOLD:AAU2328	658[0n]
**Chrysis horridula**	BC ZSM HYM 12746	Germany	19-Jul-2011	SNSB, Zoologische Staatssammlung Muenchen	BOLD:AAU2328	629[0n]
**Chrysis horridula**	BC ZSM HYM 12747	Germany	19-Jul-2011	SNSB, Zoologische Staatssammlung Muenchen	BOLD:AAU2328	581[0n]
**Chrysis horridula**	BC ZSM HYM 19913	Germany	15-May-2013	SNSB, Zoologische Staatssammlung Muenchen	BOLD:AAU2328	658[0n]
**Chrysis ignita**	ON6945	Germany	17-May-2014	Research Collection of Oliver Niehuis	BOLD:AAG0244	676[0n]
**Chrysis ignita**	ON6146	Germany	02-Jul-2013	Research Collection of Oliver Niehuis	BOLD:AAG0244	676[4n]
**Chrysis ignita**	ON4360	Germany	09-Jun-2013	Research Collection of Oliver Niehuis	BOLD:AAG0244	676[5n]
**Chrysis ignita**	ON2127	Germany	01-May-2011	Research Collection of Oliver Niehuis	BOLD:AAG0244	676[6n]
**Chrysis ignita**	ON0759	Germany	21-May-2010	Research Collection of Oliver Niehuis	BOLD:AAG0244	676[1n]
**Chrysis ignita**	ON0756	Germany	21-May-2010	Research Collection of Oliver Niehuis	BOLD:AAG0244	676[3n]
**Chrysis ignita**	ON0752	Germany	21-May-2010	Research Collection of Oliver Niehuis	BOLD:AAG0244	676[1n]
**Chrysis ignita**	ON0614	Germany	28-Jun-2009	Research Collection of Oliver Niehuis	BOLD:AAG0244	676[3n]
**Chrysis ignita**	ON0613	Germany	28-Jun-2009	Research Collection of Oliver Niehuis	BOLD:AAG0244	676[2n]
**Chrysis ignita**	ON0609	Germany	28-Jun-2009	Research Collection of Oliver Niehuis	BOLD:AAG0244	676[6n]
**Chrysis ignita**	KY430761.1_tmp	Germany	28-Jun-2009	Private Collection of Oliver Niehuis	BOLD:AAG0244	675[0n]
**Chrysis illigeri**	KY430729.1_tmp	Germany	22-May-2011	Private Collection of Oliver Niehuis	BOLD:AAV9309	675[0n]
**Chrysis illigeri**	BC ZSM HYM 15249	Germany	12-Aug-2012	SNSB, Zoologische Staatssammlung Muenchen	BOLD:AAV9309	658[0n]
**Chrysis illigeri**	BC ZSM HYM 15250	Germany	12-Aug-2012	SNSB, Zoologische Staatssammlung Muenchen	BOLD:AAV9309	658[0n]
**Chrysis illigeri**	BC ZSM HYM 07339	Italy	11-Jul-2009	SNSB, Zoologische Staatssammlung Muenchen	BOLD:AAV9309	658[0n]
**Chrysis illigeri**	BC ZSM HYM 07340	Italy	11-Jul-2009	SNSB, Zoologische Staatssammlung Muenchen	BOLD:AAV9309	658[0n]
**Chrysis illigeri**	BC ZSM HYM 07341	Italy	11-Jul-2009	SNSB, Zoologische Staatssammlung Muenchen	BOLD:AAV9309	658[0n]

**Chrysis illigeri**	BC ZSM HYM 07840	Germany	14-Jun-2010	SNSB, Zoologische Staatssammlung Muenchen	BOLD:AAV9309	658[0n]
**Chrysis illigeri**	BC ZSM HYM 07841	Germany	14-Jun-2010	SNSB, Zoologische Staatssammlung Muenchen	BOLD:AAV9309	658[0n]
**Chrysis illigeri**	BC ZSM HYM 07842	Germany	14-Jun-2010	SNSB, Zoologische Staatssammlung Muenchen	BOLD:AAV9309	658[0n]
**Chrysis illigeri**	BC ZSM HYM 07843	Germany	14-Jun-2010	SNSB, Zoologische Staatssammlung Muenchen	BOLD:AAV9309	658[0n]
**Chrysis illigeri**	BC ZSM HYM 12755	Germany	15-Jun-2011	SNSB, Zoologische Staatssammlung Muenchen	BOLD:AAV9309	658[0n]
**Chrysis illigeri**	BC ZSM HYM 12756	Germany	15-Jun-2011	SNSB, Zoologische Staatssammlung Muenchen	BOLD:AAV9309	658[0n]
**Chrysis illigeri**	BC ZSM HYM 12758	Germany	09-Jun-1998	SNSB, Zoologische Staatssammlung Muenchen	BOLD:AAV9309	617[0n]
**Chrysis illigeri**	BC ZSM HYM 21084	Germany	08-Jun-2013	SNSB, Zoologische Staatssammlung Muenchen	BOLD:AAV9309	658[0n]
**Chrysis illigeri**	BC ZSM HYM 19916	Germany	09-Jun-2013	SNSB, Zoologische Staatssammlung Muenchen	BOLD:AAV9309	655[0n]
**Chrysis illigeri**	BC ZSM HYM 14962	Germany	19-Jun-2012	SNSB, Zoologische Staatssammlung Muenchen	BOLD:AAV9309	658[0n]
**Chrysis immaculata**	KY430762.1_tmp	Germany	02-Jul-2014	Private Collection of Oliver Niehuis	BOLD:AEC7328	675[0n]
**Chrysis impressa**	ON9033	Netherlands	15-May-2016	Research Collection of Oliver Niehuis	BOLD:AAG0244	676[0n]
**Chrysis impressa**	ON9041	Netherlands	15-May-2016	Research Collection of Oliver Niehuis	BOLD:AAG0244	676[0n]
**Chrysis impressa**	ON8207	Germany	01-Jul-2015	Research Collection of Oliver Niehuis	BOLD:AAG0244	676[0n]
**Chrysis impressa**	ON8138	Germany	01-Jul-2015	Research Collection of Oliver Niehuis	BOLD:AAG0244	676[0n]
**Chrysis impressa**	BC-ZSM-HYM-29774-H05	Germany	17-Jul-2008	SNSB, Zoologische Staatssammlung Muenchen	BOLD:AAG0244	630[0n]
**Chrysis impressa**	KY430763.1_tmp	Sweden	17-Jun-2013	Private Collection of Oliver Niehuis	BOLD:AAG0244	675[0n]
**Chrysis impressa**	BC ZSM HYM 12751	Germany	06-Jul-2011	SNSB, Zoologische Staatssammlung Muenchen	BOLD:AAG0244	658[0n]
**Chrysis impressa**	BC ZSM HYM 12743	Germany	06-Jun-2011	SNSB, Zoologische Staatssammlung Muenchen	BOLD:AAG0244	658[0n]
**Chrysis impressa**	BC ZSM HYM 07999	Germany	03-May-2007	SNSB, Zoologische Staatssammlung Muenchen	BOLD:AAG0244	658[0n]
**Chrysis inaequalis**	KY430795.1_tmp	Germany	04-Jun-2011	Private Collection of Oliver Niehuis	BOLD:AAY6948	675[0n]
**Chrysis inaequalis**	BC ZSM HYM 07844	Germany	04-Jun-2010	SNSB, Zoologische Staatssammlung Muenchen	BOLD:AAY6948	655[0n]
**Chrysis inaequalis**	BC ZSM HYM 07845	Germany	04-Jun-2010	SNSB, Zoologische Staatssammlung Muenchen	BOLD:AAY6948	632[0n]
**Chrysis inaequalis**	BC ZSM HYM 07860	Germany	04-Jun-2010	SNSB, Zoologische Staatssammlung Muenchen	BOLD:AAY6948	658[0n]

**Chrysis inaequalis**	BC ZSM HYM 07861	Germany	04-Jun-2010	SNSB, Zoologische Staatssammlung Muenchen	BOLD:AAY6948	632[0n]
**Chrysis inaequalis**	BC ZSM HYM 12137	Germany	14-Jun-2010	SNSB, Zoologische Staatssammlung Muenchen	BOLD:AAY6948	558[0n]
**Chrysis indigotea**	KY430778.1_tmp	Germany	25-May-2011	Private Collection of Oliver Niehuis	BOLD:AAP1364	586[0n]
**Chrysis indigotea**	BC ZSM HYM 06298	Germany	01-Jul-2008	SNSB, Zoologische Staatssammlung Muenchen	BOLD:AAP1364	658[0n]
**Chrysis indigotea**	BC ZSM HYM 06299	Germany	01-Jul-2008	SNSB, Zoologische Staatssammlung Muenchen	BOLD:AAP1364	658[0n]
**Chrysis iris**	KJ398876	Lithuania	Jun-Aug-2008	Mined from GenBank, NCBI	BOLD:ABU6374	655[0n]
**Chrysis iris**	KJ398877	Lithuania	Jun-Aug-2008	Mined from GenBank, NCBI	BOLD:ABU6374	655[0n]
**Chrysis iris**	KY430775.1_tmp	Germany	12-Jun-2011	Private Collection of Oliver Niehuis	BOLD:ABU6374	675[0n]
**Chrysis lanceolata**	BC ZSM HYM 07873	Germany	15-Jul-2006	SNSB, Zoologische Staatssammlung Muenchen	BOLD:AAY6950	658[0n]
**Chrysis leachii**	BC ZSM HYM 07323	France	13-Jul-2009	SNSB, Zoologische Staatssammlung Muenchen	BOLD:AAY6925	658[0n]
**Chrysis leachii**	BC ZSM HYM 07324	France	13-Jul-2009	SNSB, Zoologische Staatssammlung Muenchen	BOLD:AAY6925	627[0n]
**Chrysis leachii**	BC ZSM HYM 07805	France	12-Jul-2010	SNSB, Zoologische Staatssammlung Muenchen	BOLD:AAY6925	658[0n]
**Chrysis leptomandibularis**	BC-ZSM-HYM-29774-F04	Germany	31-Aug-2008	SNSB, Zoologische Staatssammlung Muenchen	BOLD:ACQ4597	633[0n]
**Chrysis leptomandibularis**	KY430765.1_tmp	Italy	21-May-2011	Private Collection of Oliver Niehuis	BOLD:ACQ4597	675[0n]
**Chrysis longula**	ON3020	Germany	24-Jun-2012	Research Collection of Oliver Niehuis	BOLD:ACF9032	676[0n]
**Chrysis longula**	ON3025	Germany	24-Jun-2012	Research Collection of Oliver Niehuis	BOLD:ACF9032	676[0n]
**Chrysis longula**	ON3133	Germany	21-May-2012	Research Collection of Oliver Niehuis	BOLD:ACF9032	676[0n]
**Chrysis longula**	ON4364	Germany	28-May-2013	Research Collection of Oliver Niehuis	BOLD:ACF9032	676[0n]
**Chrysis longula**	ON4366	Germany	28-May-2013	Research Collection of Oliver Niehuis	BOLD:ACF9032	676[0n]
**Chrysis longula**	ON4555	Germany	15-May-2012	Research Collection of Oliver Niehuis	BOLD:ACF9032	676[0n]
**Chrysis longula**	ON4561	Germany	15-May-2012	Research Collection of Oliver Niehuis	BOLD:ACF9032	676[0n]
**Chrysis longula**	ON5512	Germany	15-May-2012	Research Collection of Oliver Niehuis	BOLD:ACF9032	658[0n]
**Chrysis longula**	ON6817	Germany	04-May-2014	Research Collection of Oliver Niehuis	BOLD:ACF9032	676[0n]
**Chrysis longula**	ON6818	Germany	04-May-2014	Research Collection of Oliver Niehuis	BOLD:ACF9032	676[0n]

**Chrysis longula**	ON6819	Germany	04-May-2014	Research Collection of Oliver Niehuis	BOLD:ACF9032	676[0n]
**Chrysis longula**	ON6820	Germany	04-May-2014	Research Collection of Oliver Niehuis	BOLD:ACF9032	676[0n]
**Chrysis longula**	ON6823	Germany	04-May-2014	Research Collection of Oliver Niehuis	BOLD:ACF9032	676[0n]
**Chrysis longula**	ON6824	Germany	04-May-2014	Research Collection of Oliver Niehuis	BOLD:ACF9032	676[0n]
**Chrysis longula**	ON6825	Germany	04-May-2014	Research Collection of Oliver Niehuis	BOLD:ACF9032	676[0n]
**Chrysis longula**	ON6827	Germany	04-May-2014	Research Collection of Oliver Niehuis	BOLD:ACF9032	676[0n]
**Chrysis longula**	ON6828	Germany	04-May-2014	Research Collection of Oliver Niehuis	BOLD:ACF9032	676[0n]
**Chrysis longula**	ON6951	Germany	17-May-2014	Research Collection of Oliver Niehuis	BOLD:ACF9032	676[0n]
**Chrysis longula**	BC-ZSM-HYM-29774-E05	Germany	15-Jun-2009	SNSB, Zoologische Staatssammlung Muenchen	BOLD:ACF9032	628[0n]
**Chrysis longula**	BC-ZSM-HYM-29774-E06	Germany	01-Jun-2017	SNSB, Zoologische Staatssammlung Muenchen	BOLD:ACF9032	628[0n]
**Chrysis longula**	BC-ZSM-HYM-29774-E07	Germany	07-Jun-2013	SNSB, Zoologische Staatssammlung Muenchen	BOLD:ACF9032	628[0n]
**Chrysis longula**	BC-ZSM-HYM-29774-E08	Germany	07-Jun-2011	SNSB, Zoologische Staatssammlung Muenchen	BOLD:ACF9032	629[0n]
**Chrysis longula**	BC-ZSM-HYM-29774-G11	Germany	09-Jun-2010	SNSB, Zoologische Staatssammlung Muenchen	BOLD:ACF9032	628[0n]
**Chrysis longula**	KY430770.1_tmp	Germany	11-May-2012	Private Collection of Oliver Niehuis	BOLD:ACF9032	675[0n]
**Chrysis longula**	BC ZSM HYM 12750	Germany	06-Jul-2011	SNSB, Zoologische Staatssammlung Muenchen	BOLD:ACF9032	658[0n]
**Chrysis marginata**	KY430714.1_tmp	Greece	12-May-2013	Private Collection of Oliver Niehuis	BOLD:ACG4638	675[0n]
**Chrysis marginata**	BC ZSM HYM 17078	Germany	08-Jul-2007	SNSB, Zoologische Staatssammlung Muenchen	BOLD:ACG4638	658[0n]
**Chrysis mediata**	ON0122	Germany	08-Jun-1999	Research Collection of Oliver Niehuis	BOLD:AAY6949	676[0n]
**Chrysis mediata**	ON0463	Germany	30-May-2009	Research Collection of Oliver Niehuis	BOLD:AAY6949	676[0n]
**Chrysis mediata**	ON0605	Germany	28-Jun-2009	Research Collection of Oliver Niehuis	BOLD:AAY6949	676[0n]
**Chrysis mediata**	ON0748	Germany	25-May-2010	Research Collection of Oliver Niehuis	BOLD:AAY6949	676[0n]
**Chrysis mediata**	ON4629	Germany	15-May-2013	Research Collection of Oliver Niehuis	BOLD:AAY6949	676[0n]
**Chrysis mediata**	ON5538	Germany	15-May-2013	Research Collection of Oliver Niehuis	BOLD:AAY6949	676[0n]
**Chrysis mediata**	ON6150	Germany	02-Jul-2013	Research Collection of Oliver Niehuis	BOLD:AAY6949	676[0n]

**Chrysis mediata**	ON7924	Germany	16-May-2015	Research Collection of Oliver Niehuis	BOLD:AAY6949	676[0n]
**Chrysis mediata**	ON2244	Germany	09-Jun-2010	Research Collection of Oliver Niehuis	BOLD:AAY6949	676[0n]
**Chrysis mediata**	ON1489	Germany	02-Jun-2011	Research Collection of Oliver Niehuis	BOLD:AAY6949	676[0n]
**Chrysis mediata**	ON1867	Germany	15-May-2007	Research Collection of Oliver Niehuis	BOLD:AAY6949	663[3n]
**Chrysis mediata**	BC-ZSM-HYM-29774-G10	Germany	31-May-2007	SNSB, Zoologische Staatssammlung Muenchen	BOLD:AAY6949	629[0n]
**Chrysis mediata**	BC-ZSM-HYM-29774-H09	Germany	31-May-2007	SNSB, Zoologische Staatssammlung Muenchen	BOLD:AAY6949	636[1n]
**Chrysis mediata**	KY430767.1_tmp	Italy	18-Jun-2011	Private Collection of Oliver Niehuis	BOLD:AAY6949	675[0n]
**Chrysis mediata**	BC ZSM HYM 07862	Germany	06-May-2003	SNSB, Zoologische Staatssammlung Muenchen	BOLD:AAY6949	658[0n]
**Chrysis mediata**	BC ZSM HYM 07863	Germany	06-May-2003	SNSB, Zoologische Staatssammlung Muenchen	BOLD:AAY6949	658[0n]
**Chrysis mediata**	BC ZSM HYM 07864	Germany	20-May-2002	SNSB, Zoologische Staatssammlung Muenchen	BOLD:AAY6949	658[0n]
**Chrysis mediata**	BC ZSM HYM 07865	Germany	20-May-2009	SNSB, Zoologische Staatssammlung Muenchen	BOLD:AAY6949	658[0n]
**Chrysis mediata**	BC ZSM HYM 12154	Germany	11-May-2000	SNSB, Zoologische Staatssammlung Muenchen	BOLD:AAY6949	583[0n]
**Chrysis parabrevitarsis**	ON4369	Germany	28-May-2013	Research Collection of Oliver Niehuis	BOLD:ACG6749	676[0n]
**Chrysis parabrevitarsis**	ON4411	Germany	15-Jul-2013	Research Collection of Oliver Niehuis	BOLD:ACG6749	676[0n]
**Chrysis parabrevitarsis**	ON4557	Germany	15-May-2012	Research Collection of Oliver Niehuis	BOLD:ACG6749	676[0n]
**Chrysis parabrevitarsis**	ON5349	Germany	09-Jul-2013	Research Collection of Oliver Niehuis	BOLD:ACG6749	676[0n]
**Chrysis parabrevitarsis**	ON6711	Germany	04-May-2014	Research Collection of Oliver Niehuis	BOLD:ACG6749	676[0n]
**Chrysis parabrevitarsis**	ON6955	Germany	30-May-2014	Research Collection of Oliver Niehuis	BOLD:ACG6749	676[0n]
**Chrysis parabrevitarsis**	ON3143	Germany	19-May-2012	Research Collection of Oliver Niehuis	BOLD:ACG6749	676[0n]
**Chrysis parabrevitarsis**	ON3194	Germany	20-May-2012	Research Collection of Oliver Niehuis	BOLD:ACG6749	676[0n]
**Chrysis parabrevitarsis**	ON3197	Germany	22-Jun-2012	Research Collection of Oliver Niehuis	BOLD:ACG6749	676[0n]
**Chrysis parabrevitarsis**	ON3242	Germany	10-Jun-2012	Research Collection of Oliver Niehuis	BOLD:ACG6749	676[0n]
**Chrysis parabrevitarsis**	ON4394	Germany	09-Jul-2013	Research Collection of Oliver Niehuis	BOLD:ACG6749	676[0n]

**Chrysis parabrevitarsis**	ON4440	Germany	08-Jun-2013	Research Collection of Oliver Niehuis	BOLD:ACG6749	676[0n]
**Chrysis parabrevitarsis**	ON4445	Germany	08-Jun-2013	Research Collection of Oliver Niehuis	BOLD:ACG6749	676[0n]
**Chrysis parabrevitarsis**	ON4559	Germany	15-May-2012	Research Collection of Oliver Niehuis	BOLD:ACG6749	676[0n]
**Chrysis parabrevitarsis**	ON4560	Germany	15-May-2012	Research Collection of Oliver Niehuis	BOLD:ACG6749	676[0n]
**Chrysis parabrevitarsis**	ON4657	Germany	15-Jul-2013	Research Collection of Oliver Niehuis	BOLD:ACG6749	676[0n]
**Chrysis parabrevitarsis**	ON4668	Germany	23-Jun-2013	Research Collection of Oliver Niehuis	BOLD:ACG6749	676[0n]
**Chrysis parabrevitarsis**	ON4673	Germany	21-Jul-2013	Research Collection of Oliver Niehuis	BOLD:ACF7346	676[0n]
**Chrysis parabrevitarsis**	ON4679	Germany	15-Jun-2013	Research Collection of Oliver Niehuis	BOLD:ACG6749	676[0n]
**Chrysis parabrevitarsis**	ON5964	Germany	30-Jun-2013	Research Collection of Oliver Niehuis	BOLD:ACG6749	676[0n]
**Chrysis parabrevitarsis**	ON6006	Germany	08-Jun-2013	Research Collection of Oliver Niehuis	BOLD:ACG6749	676[0n]
**Chrysis parabrevitarsis**	ON7911	Germany	16-May-2015	Research Collection of Oliver Niehuis	BOLD:ACG6749	676[0n]
**Chrysis parabrevitarsis**	ON8235	Germany	17-Jun-2015	Research Collection of Oliver Niehuis	BOLD:ACG6749	663[0n]
**Chrysis parabrevitarsis**	KY430768.1_tmp	Germany	20-May-2012	Private Collection of Oliver Niehuis	BOLD:ACG6749	675[0n]
**Chrysis parabrevitarsis**	BC ZSM HYM 12742	Germany	06-Jun-2011	SNSB, Zoologische Staatssammlung Muenchen	BOLD:ACG6749	658[0n]
**Chrysis parabrevitarsis**	BC ZSM HYM 12748	Germany	07-Jul-2011	SNSB, Zoologische Staatssammlung Muenchen	BOLD:ACG6749	658[0n]
**Chrysis parabrevitarsis**	BC ZSM HYM 12745	Germany	06-Jun-2011	SNSB, Zoologische Staatssammlung Muenchen	BOLD:ACG6749	658[0n]
**Chrysis parietis**	ON1473	Germany	02-Jun-2011	Research Collection of Oliver Niehuis	BOLD:AAU2329	676[0n]
**Chrysis parietis**	ON3462	Hungary	24-Sep-2007	Research Collection of Oliver Niehuis	BOLD:AAU2329	676[0n]
**Chrysis parietis**	ON3468	Germany	15-May-2012	Research Collection of Oliver Niehuis		658[8n]
**Chrysis parietis**	ON3469	Germany	15-May-2012	Research Collection of Oliver Niehuis	BOLD:AAU2329	676[0n]
**Chrysis parietis**	ON7396	Germany	27-Apr-2012	Research Collection of Oliver Niehuis	BOLD:AAU2329	676[0n]
**Chrysis parietis**	BC-ZSM-HYM-29774-F07	Germany	28-May-2017	SNSB, Zoologische Staatssammlung Muenchen	BOLD:AAU2329	631[0n]
**Chrysis parietis**	BC-ZSM-HYM-29774-H01	Germany	22-May-2012	SNSB, Zoologische Staatssammlung Muenchen	BOLD:AAU2329	632[0n]
**Chrysis parietis**	BC-ZSM-HYM-29774-H10	Germany	05-Jun-2013	SNSB, Zoologische Staatssammlung Muenchen	BOLD:AAU2329	633[0n]

**Chrysis parietis**	BC-ZSM-HYM-29771-E03	Germany	10-Jun-2017	SNSB, Zoologische Staatssammlung Muenchen	BOLD:AAU2329	640[0n]
**Chrysis parietis**	BC ZSM HYM 07998	Germany	03-Jun-2007	SNSB, Zoologische Staatssammlung Muenchen	BOLD:AAU2329	658[0n]
**Chrysis parietis**	BC ZSM HYM 08000	Germany	14-Jun-2001	SNSB, Zoologische Staatssammlung Muenchen	BOLD:AAU2329	658[2n]
**Chrysis parietis**	ON_14624	Germany	24-Apr-2021	Private Collection of Oliver Niehuis	BOLD:AAU2329	664[0n]
**Chrysis parietis**	ON_14623	Germany	24-Apr-2021	Private Collection of Oliver Niehuis	BOLD:AAU2329	609[0n]
**Chrysis pseudobrevitarsis**	ON4612	Germany	07-Jul-2013	Research Collection of Oliver Niehuis	BOLD:ACG6983	676[0n]
**Chrysis pseudobrevitarsis**	ON4619	Germany	07-Jul-2013	Research Collection of Oliver Niehuis	BOLD:ACG6983	676[0n]
**Chrysis pseudobrevitarsis**	ON7777	Germany	22-Jun-2014	Research Collection of Oliver Niehuis	BOLD:ACG6983	658[0n]
**Chrysis pseudobrevitarsis**	ON9821	Germany	06-May-1997	Research Collection of Oliver Niehuis	BOLD:ACG6983	676[2n]
**Chrysis pulchella**	KY430796.1_tmp	Spain	09-Jun-2011	Private Collection of Oliver Niehuis	BOLD:AED0619	675[0n]
**Chrysis ruddii**	KY430773.1_tmp	Germany	01-May-2011	Private Collection of Oliver Niehuis	BOLD:AAY6944	675[0n]
**Chrysis ruddii**	BC ZSM HYM 07798	France	15-Jul-2010	SNSB, Zoologische Staatssammlung Muenchen	BOLD:AAY6944	658[0n]
**Chrysis ruddii**	BC ZSM HYM 07874	Germany	30-May-2006	SNSB, Zoologische Staatssammlung Muenchen	BOLD:AAY6944	658[0n]
**Chrysis ruddii**	BC ZSM HYM 07875	Germany	22-May-2007	SNSB, Zoologische Staatssammlung Muenchen	BOLD:AAY6944	658[0n]
**Chrysis ruddii**	BC ZSM HYM 07876	Germany	21-May-2005	SNSB, Zoologische Staatssammlung Muenchen	BOLD:AAY6944	658[0n]
**Chrysis ruddii**	BC ZSM HYM 07877	Germany	24-Apr-2010	SNSB, Zoologische Staatssammlung Muenchen	BOLD:AAY6944	658[0n]
**Chrysis ruddii**	BC ZSM HYM 12159	Germany	07-Jun-2007	SNSB, Zoologische Staatssammlung Muenchen	BOLD:AAY6944	609[0n]
**Chrysis rutilans**	BC ZSM HYM 11086	Germany	16-Jul-2011	SNSB, Zoologische Staatssammlung Muenchen	BOLD:ABA9738	658[0n]
**Chrysis rutilans**	BC ZSM HYM 17347	Germany	18-Jul-2012	SNSB, Zoologische Staatssammlung Muenchen	BOLD:ABA9738	658[0n]
**Chrysis rutilans**	BC ZSM HYM 17348	Germany	18-Jul-2012	SNSB, Zoologische Staatssammlung Muenchen	BOLD:ABA9738	621[0n]
**Chrysis rutilans**	BC ZSM HYM 17349	Germany	18-Jul-2012	SNSB, Zoologische Staatssammlung Muenchen	BOLD:ABA9738	658[0n]
**Chrysis rutilans**	BC ZSM HYM 17350	Germany	18-Jul-2012	SNSB, Zoologische Staatssammlung Muenchen	BOLD:ABA9738	658[0n]
**Chrysis rutiliventris**	KY430772.1_tmp	France	11-Jul-2011	Private Collection of Oliver Niehuis	BOLD:AAY6931	675[0n]

**Chrysis rutiliventris**	BC ZSM HYM 07344	Italy	13-Aug-2008	SNSB, Zoologische Staatssammlung Muenchen	BOLD:AAY6931	658[0n]
**Chrysis rutiliventris**	BC ZSM HYM 07345	Italy	09-Jul-2006	SNSB, Zoologische Staatssammlung Muenchen	BOLD:AAY6931	658[0n]
**Chrysis rutiliventris**	BC ZSM HYM 07797	France	15-Jul-2010	SNSB, Zoologische Staatssammlung Muenchen	BOLD:AAY6931	658[0n]
**Chrysis schencki**	ON11387	Germany	15-May-2017	Research Collection of Oliver Niehuis	BOLD:ABU6375	668[0n]
**Chrysis schencki**	ON11502	Germany	15-May-2017	Research Collection of Oliver Niehuis	BOLD:ABU6375	657[0n]
**Chrysis schencki**	ON11078	Germany	15-May-2017	Research Collection of Oliver Niehuis	BOLD:ACF6219	676[0n]
**Chrysis schencki**	ON9715	Germany	15-May-2017	Research Collection of Oliver Niehuis	BOLD:ABU6375	657[0n]
**Chrysis schencki**	ON9725	Germany	15-May-2017	Research Collection of Oliver Niehuis	BOLD:ABU6375	676[0n]
**Chrysis schencki**	ON11112	Germany	15-May-2017	Research Collection of Oliver Niehuis	BOLD:ABU6375	676[0n]
**Chrysis schencki**	ON10498	Germany	15-May-2017	Research Collection of Oliver Niehuis	BOLD:ABU6375	676[0n]
**Chrysis schencki**	ON9718	Germany	15-May-2017	Research Collection of Oliver Niehuis	BOLD:ABU6375	676[0n]
**Chrysis schencki**	ON11472	Germany	15-May-2017	Research Collection of Oliver Niehuis	BOLD:ABU6375	676[0n]
**Chrysis schencki**	ON11469	Germany	15-May-2017	Research Collection of Oliver Niehuis	BOLD:ACF6219	676[0n]
**Chrysis schencki**	ON11515	Germany	15-May-2017	Research Collection of Oliver Niehuis	BOLD:ACF6219	655[0n]
**Chrysis schencki**	ON11516	Germany	15-May-2017	Research Collection of Oliver Niehuis	BOLD:ACF6219	676[0n]
**Chrysis schencki**	ON11523	Germany	15-May-2017	Research Collection of Oliver Niehuis	BOLD:ACF6219	676[0n]
**Chrysis schencki**	ON11531	Germany	15-May-2017	Research Collection of Oliver Niehuis	BOLD:ACF6219	676[0n]
**Chrysis schencki**	ON11535	Germany	15-May-2017	Research Collection of Oliver Niehuis	BOLD:ACF6219	676[0n]
**Chrysis schencki**	ON11488	Germany	15-May-2017	Research Collection of Oliver Niehuis	BOLD:ACF6219	676[0n]
**Chrysis schencki**	ON11489	Germany	15-May-2017	Research Collection of Oliver Niehuis	BOLD:ACF6219	676[0n]
**Chrysis schencki**	ON11490	Germany	15-May-2017	Research Collection of Oliver Niehuis	BOLD:ACF6219	676[0n]
**Chrysis schencki**	ON11491	Germany	15-May-2017	Research Collection of Oliver Niehuis	BOLD:ACF6219	676[0n]
**Chrysis schencki**	ON11492	Germany	15-May-2017	Research Collection of Oliver Niehuis	BOLD:ACF6219	676[0n]
**Chrysis schencki**	ON9717	Germany	15-May-2017	Research Collection of Oliver Niehuis	BOLD:ACF6219	665[0n]

**Chrysis schencki**	ON9737	Germany	15-May-2017	Research Collection of Oliver Niehuis	BOLD:ACF6219	676[0n]
**Chrysis schencki**	ON11201	Germany	15-May-2017	Research Collection of Oliver Niehuis	BOLD:ACF6219	676[0n]
**Chrysis schencki**	ON11395	Germany	15-May-2017	Research Collection of Oliver Niehuis	BOLD:ACF6219	676[0n]
**Chrysis schencki**	ON9789	Germany	15-May-2017	Research Collection of Oliver Niehuis	BOLD:ACF6219	676[0n]
**Chrysis schencki**	ON9738	Germany	15-May-2017	Research Collection of Oliver Niehuis	BOLD:ACF6219	676[0n]
**Chrysis schencki**	ON9712	Germany	15-May-2017	Research Collection of Oliver Niehuis	BOLD:ACF6219	676[0n]
**Chrysis schencki**	ON9711	Germany	15-May-2017	Research Collection of Oliver Niehuis	BOLD:ACF6219	676[0n]
**Chrysis schencki**	ON9713	Germany	15-May-2017	Research Collection of Oliver Niehuis	BOLD:ACF6219	676[0n]
**Chrysis schencki**	ON9719	Germany	15-May-2017	Research Collection of Oliver Niehuis	BOLD:ACF6219	664[0n]
**Chrysis schencki**	ON11413	Germany	15-May-2017	Research Collection of Oliver Niehuis	BOLD:ACF6219	676[0n]
**Chrysis schencki**	ON11079	Germany	15-May-2017	Research Collection of Oliver Niehuis	BOLD:ACF6219	676[0n]
**Chrysis schencki**	ON11085	Germany	15-May-2017	Research Collection of Oliver Niehuis	BOLD:ACF6219	676[0n]
**Chrysis schencki**	ON11110	Germany	15-May-2017	Research Collection of Oliver Niehuis	BOLD:ACF6219	676[0n]
**Chrysis schencki**	ON11496	Germany	15-May-2017	Research Collection of Oliver Niehuis	BOLD:ACF6219	676[0n]
**Chrysis schencki**	BC-ZSM-HYM-29774-E11	Germany	17-Sep-2007	SNSB, Zoologische Staatssammlung Muenchen	BOLD:ACF6219	636[0n]
**Chrysis schencki**	BC-ZSM-HYM-29774-E12	Germany	23-Jun-2008	SNSB, Zoologische Staatssammlung Muenchen	BOLD:ACF6219	633[0n]
**Chrysis schencki**	BC-ZSM-HYM-29774-F01	Germany	13-Aug-2011	SNSB, Zoologische Staatssammlung Muenchen	BOLD:ACF6219	632[0n]
**Chrysis schencki**	BC-ZSM-HYM-29774-F03	Germany	26-Aug-2011	SNSB, Zoologische Staatssammlung Muenchen	BOLD:ACF6219	636[0n]
**Chrysis schencki**	BC-ZSM-HYM-29774-G04	Germany	21-Jun-2017	SNSB, Zoologische Staatssammlung Muenchen	BOLD:ABU6375	633[0n]
**Chrysis schencki**	BC-ZSM-HYM-29774-G05	Germany	25-Jul-2008	SNSB, Zoologische Staatssammlung Muenchen	BOLD:ACF6219	636[0n]
**Chrysis schencki**	BC-ZSM-HYM-29774-H11	Germany	24-May-2008	SNSB, Zoologische Staatssammlung Muenchen	BOLD:ACF6219	634[0n]
**Chrysis schencki**	KY430764.1_tmp	Sweden	08-Jun-2013	Private Collection of Oliver Niehuis	BOLD:ACF6219	674[0n]
**Chrysis schencki**	BC ZSM HYM 12759	Germany	23-Jun-2011	SNSB, Zoologische Staatssammlung Muenchen	BOLD:ACF6219	658[0n]

**Chrysis schencki**	BC-ZSM-HYM-29771-H11	Germany	30-Aug-2008	SNSB, Zoologische Staatssammlung Muenchen	BOLD:ACF6219	615[0n]
**Chrysis sculpturata**	ON1269	France	14-Jul-2011	Research Collection of Oliver Niehuis	BOLD:ABU6373	676[1n]
**Chrysis scutellaris**	BC ZSM HYM 06306	Italy	08-Jun-2007	SNSB, Zoologische Staatssammlung Muenchen		177[0n]
**Chrysis scutellaris**	KY430717.1_tmp	Germany	07-Jul-2012	Private Collection of Oliver Niehuis	BOLD:AAR9833	675[0n]
**Chrysis scutellaris**	BC ZSM HYM 07868	Germany	26-May-2005	SNSB, Zoologische Staatssammlung Muenchen	BOLD:AAR9833	658[0n]
**Chrysis scutellaris**	BC ZSM HYM 07337	Italy	17-Jun-2009	SNSB, Zoologische Staatssammlung Muenchen	BOLD:AAR9833	658[0n]
**Chrysis scutellaris**	BC ZSM HYM 07338	Italy	17-Jun-2009	SNSB, Zoologische Staatssammlung Muenchen	BOLD:AAR9833	658[0n]
**Chrysis scutellaris**	BC ZSM HYM 07866	Germany	05-Jul-2010	SNSB, Zoologische Staatssammlung Muenchen	BOLD:AAR9833	658[0n]
**Chrysis scutellaris**	BC ZSM HYM 07867	Germany	08-Jun-2007	SNSB, Zoologische Staatssammlung Muenchen	BOLD:AAR9833	395[0n]
**Chrysis scutellaris**	BC ZSM HYM 07869	Germany	02-Jun-2005	SNSB, Zoologische Staatssammlung Muenchen	BOLD:AAR9833	658[0n]
**Chrysis scutellaris**	BC ZSM HYM 11087	Germany	16-Jul-2011	SNSB, Zoologische Staatssammlung Muenchen	BOLD:AAR9833	658[0n]
**Chrysis scutellaris**	BC ZSM HYM 11088	Germany	16-Jul-2011	SNSB, Zoologische Staatssammlung Muenchen	BOLD:AAR9833	658[0n]
**Chrysis scutellaris**	BC ZSM HYM 17351	Italy	20-Jul-2012	SNSB, Zoologische Staatssammlung Muenchen	BOLD:AAR9833	658[0n]
**Chrysis scutellaris**	BC ZSM HYM 17352	Italy	20-Jul-2012	SNSB, Zoologische Staatssammlung Muenchen		233[2n]
**Chrysis scutellaris**	BC ZSM HYM 17354	Italy	20-Jul-2012	SNSB, Zoologische Staatssammlung Muenchen	BOLD:AAR9833	658[0n]
**Chrysis scutellaris**	BC ZSM HYM 06307	Italy	27-Jul-2007	SNSB, Zoologische Staatssammlung Muenchen	BOLD:AAR9833	658[0n]
**Chrysis solida**	ON5248	Sweden	05-Jul-2013	Research Collection of Oliver Niehuis	BOLD:AAY6949	676[0n]
**Chrysis solida**	ON0112	Germany	06-Aug-1999	Research Collection of Oliver Niehuis	BOLD:AAY6949	676[0n]
**Chrysis solida**	ON4803	Germany	17-Jun-2012	Research Collection of Oliver Niehuis	BOLD:AAY6949	676[0n]
**Chrysis solida**	ON7189	Germany	13-Jun-2014	Research Collection of Oliver Niehuis	BOLD:AAY6949	676[1n]
**Chrysis solida**	ON7535	Italy	06-Jun-2014	Research Collection of Oliver Niehuis	BOLD:AAY6949	676[0n]
**Chrysis solida**	ON7536	Italy	05-Jun-2014	Research Collection of Oliver Niehuis	BOLD:AAY6949	676[0n]
**Chrysis solida**	ON7664	Italy	15-Sep-2014	Research Collection of Oliver Niehuis	BOLD:AAY6949	676[0n]
**Chrysis solida**	ON7665	Italy	15-Sep-2014	Research Collection of Oliver Niehuis	BOLD:AAY6949	676[0n]

**Chrysis solida**	ON7667	Italy	16-Aug-2014	Research Collection of Oliver Niehuis	BOLD:AAY6949	676[0n]
**Chrysis solida**	ON7669	Italy	19-Jun-2014	Research Collection of Oliver Niehuis	BOLD:AAY6949	676[1n]
**Chrysis solida**	ON7775	Germany	13-Jun-2014	Research Collection of Oliver Niehuis	BOLD:AAY6949	676[0n]
**Chrysis solida**	ON9764	Germany	15-May-2017	Research Collection of Oliver Niehuis	BOLD:AAY6949	676[0n]
**Chrysis solida**	ON7197	Germany	13-Jun-2014	Research Collection of Oliver Niehuis	BOLD:AAY6949	676[0n]
**Chrysis solida**	ON9120	Germany	15-May-2016	Research Collection of Oliver Niehuis	BOLD:AAY6949	676[0n]
**Chrysis solida**	BC-ZSM-HYM-29774-E09	Germany	30-Jun-2008	SNSB, Zoologische Staatssammlung Muenchen	BOLD:AAY6949	634[0n]
**Chrysis solida**	BC-ZSM-HYM-29774-E10	Germany	06-Sep-2014	SNSB, Zoologische Staatssammlung Muenchen	BOLD:AAY6949	626[0n]
**Chrysis solida**	BC-ZSM-HYM-29774-G07	Germany	05-Sep-2007	SNSB, Zoologische Staatssammlung Muenchen	BOLD:AAY6949	634[0n]
**Chrysis solida**	BC-ZSM-HYM-29774-H06	Germany	21-May-2017	SNSB, Zoologische Staatssammlung Muenchen	BOLD:AAY6949	638[0n]
**Chrysis solida**	BC-ZSM-HYM-29774-H07	Germany	20-Aug-2014	SNSB, Zoologische Staatssammlung Muenchen	BOLD:AAY6949	639[0n]
**Chrysis solida**	KY430766.1_tmp	Germany	26-Jun-1998	Private Collection of Oliver Niehuis	BOLD:AAY6949	675[0n]
**Chrysis solida**	BC-ZSM-HYM-29771-E02	Germany	08-Aug-2017	SNSB, Zoologische Staatssammlung Muenchen	BOLD:AAY6949	658[0n]
**Chrysis solida**	BC ZSM HYM 12752	Germany	07-Jul-2011	SNSB, Zoologische Staatssammlung Muenchen	BOLD:AAY6949	658[0n]
**Chrysis solida**	BC ZSM HYM 12753	Germany	31-May-2011	SNSB, Zoologische Staatssammlung Muenchen	BOLD:AAY6949	658[0n]
**Chrysis solida**	BC ZSM HYM 12754	Germany	31-May-2011	SNSB, Zoologische Staatssammlung Muenchen	BOLD:AAY6949	658[0n]
**Chrysis solida**	BC-ZSM-HYM-29771-D11	Germany	24-Jul-2016	SNSB, Zoologische Staatssammlung Muenchen	BOLD:AAY6949	641[0n]
**Chrysis solida**	BC-ZSM-HYM-29771-D10	Germany	24-Jun-2016	SNSB, Zoologische Staatssammlung Muenchen	BOLD:AAY6949	634[0n]
**Chrysis splendidula**	KY430744.1_tmp	Italy	14-Jun-2011	Private Collection of Oliver Niehuis	BOLD:AAR9835	675[0n]
**Chrysis splendidula**	BC ZSM HYM 07796	France	10-Jul-2010	SNSB, Zoologische Staatssammlung Muenchen	BOLD:AAR9835	658[0n]
**Chrysis splendidula**	BC ZSM HYM 11089	Germany	09-Jul-2011	SNSB, Zoologische Staatssammlung Muenchen	BOLD:AAR9835	658[0n]
**Chrysis splendidula**	BC ZSM HYM 24680	Germany	11-Jun-2015	SNSB, Zoologische Staatssammlung Muenchen	BOLD:AAR9835	658[0n]
**Chrysis splendidula**	BC ZSM HYM 12900	Czech Republic	26-Aug-2011	SNSB, Zoologische Staatssammlung Muenchen	BOLD:AAR9835	658[0n]

**Chrysis subcoriacea**	BC-ZSM-HYM-29774-H04	Germany	12-Jun-2008	SNSB, Zoologische Staatssammlung Muenchen	BOLD:ACF9031	630[0n]
**Chrysis subcoriacea**	BC ZSM HYM 12744	Germany	06-Jul-2011	SNSB, Zoologische Staatssammlung Muenchen	BOLD:ACF9031	658[0n]
**Chrysis subcoriacea**	BC ZSM HYM 17477	Italy	20-Jul-2012	SNSB, Zoologische Staatssammlung Muenchen	BOLD:ACF9031	658[0n]
**Chrysis succincta**	KY430731.1_tmp	France	11-Jul-2011	Private Collection of Oliver Niehuis	BOLD:AEC9270	675[0n]
**Chrysis succincta**	ZSM-HYM-29808-F09	Germany	30-Jul-2021	SNSB, Zoologische Staatssammlung Muenchen	BOLD:AEC9270	655[0n]
**Chrysis succincta**	ZSM-HYM-29808-F08	Germany	06-Aug-2021	SNSB, Zoologische Staatssammlung Muenchen	BOLD:AEC9270	655[0n]
**Chrysis succincta**	ZSM-HYM-29808-F07	Germany	06-Aug-2021	SNSB, Zoologische Staatssammlung Muenchen	BOLD:AEC9270	655[0n]
**Chrysis succincta**	ZSM-HYM-29808-F06	Germany	06-Aug-2021	SNSB, Zoologische Staatssammlung Muenchen	BOLD:AEC9270	655[0n]
**Chrysis sybarita**	KY430779.1_tmp	Germany	17-Jun-1999	Private Collection of Oliver Niehuis	BOLD:AAJ4866	675[0n]
**Chrysis sybarita**	BC ZSM HYM 07836	Germany	06-May-2008	SNSB, Zoologische Staatssammlung Muenchen	BOLD:AAJ4866	658[0n]
**Chrysis sybarita**	BC ZSM HYM 07837	Germany	19-May-2004	SNSB, Zoologische Staatssammlung Muenchen	BOLD:AAJ4866	658[0n]
**Chrysis sybarita**	BC ZSM HYM 07838	Germany	28-May-2004	SNSB, Zoologische Staatssammlung Muenchen	BOLD:AAJ4866	658[0n]
**Chrysis sybarita**	BC ZSM HYM 07839	Germany	21-May-2005	SNSB, Zoologische Staatssammlung Muenchen	BOLD:AAJ4866	658[1n]
**Chrysis sybarita**	BC ZSM HYM 06283	Germany	19-Jul-2008	SNSB, Zoologische Staatssammlung Muenchen	BOLD:AAJ4866	649[0n]
**Chrysis terminata**	ON5241	Sweden	08-Jun-2013	Research Collection of Oliver Niehuis	BOLD:ABY5626	676[0n]
**Chrysis terminata**	ON5953	Germany	07-Jun-2013	Research Collection of Oliver Niehuis	BOLD:ABY5626	676[0n]
**Chrysis terminata**	ON7214	Germany	19-May-2014	Research Collection of Oliver Niehuis	BOLD:ABY5626	676[0n]
**Chrysis terminata**	ON7242	Germany	02-Jul-2014	Research Collection of Oliver Niehuis	BOLD:ABY5626	676[0n]
**Chrysis terminata**	ON7569	Italy	05-Jun-2014	Research Collection of Oliver Niehuis	BOLD:ABY5626	676[0n]
**Chrysis terminata**	ON9404	Germany	10-May-2017	Research Collection of Oliver Niehuis	BOLD:ABY5626	676[0n]
**Chrysis terminata**	BC-ZSM-HYM-29774-F02	Germany	24-Jun-2011	SNSB, Zoologische Staatssammlung Muenchen	BOLD:ABY5626	626[0n]
**Chrysis terminata**	BC-ZSM-HYM-29774-F10	Germany	30-Jun-2008	SNSB, Zoologische Staatssammlung Muenchen	BOLD:ABY5626	638[0n]
**Chrysis terminata**	BC-ZSM-HYM-29774-F11	Germany	24-Aug-2013	SNSB, Zoologische Staatssammlung Muenchen	BOLD:ABY5626	636[0n]
**Chrysis terminata**	BC-ZSM-HYM-29774-F12	Germany	24-Jun-2009	SNSB, Zoologische Staatssammlung Muenchen	BOLD:ABY5626	624[0n]

**Chrysis terminata**	BC-ZSM-HYM-29774-G12	Germany	08-Jul-2017	SNSB, Zoologische Staatssammlung Muenchen	BOLD:ABY5626	635[0n]
**Chrysis terminata**	BC-ZSM-HYM-29774-H02	Germany	19-Jun-2016	SNSB, Zoologische Staatssammlung Muenchen	BOLD:ABY5626	635[0n]
**Chrysis terminata**	BC-ZSM-HYM-29774-H08	Germany	25-May-2015	SNSB, Zoologische Staatssammlung Muenchen	BOLD:ABY5626	636[0n]
**Chrysis terminata**	KY430760.1_tmp	Germany	04-Jun-2011	Private Collection of Oliver Niehuis	BOLD:ABY5626	675[0n]
**Chrysis terminata**	BC ZSM HYM 12738	Germany	22-May-2010	SNSB, Zoologische Staatssammlung Muenchen	BOLD:ABY5626	658[0n]
**Chrysis terminata**	BC-ZSM-HYM-29771-E04	Germany	27-May-2017	SNSB, Zoologische Staatssammlung Muenchen	BOLD:ABY5626	640[0n]
**Chrysis terminata**	BC-ZSM-HYM-29771-E08	Germany	13-Apr-2014	SNSB, Zoologische Staatssammlung Muenchen	BOLD:ABY5626	630[0n]
**Chrysis terminata**	BC ZSM HYM 12749	Germany	15-Jun-2011	SNSB, Zoologische Staatssammlung Muenchen	BOLD:ABY5626	658[0n]
**Chrysis terminata**	BC-ZSM-HYM-29771-H09	Germany	10-Jun-2009	SNSB, Zoologische Staatssammlung Muenchen	BOLD:ABY5626	658[0n]
**Chrysis terminata**	BC ZSM HYM 11091	Germany	09-Jul-2011	SNSB, Zoologische Staatssammlung Muenchen	BOLD:ABY5626	658[0n]
**Chrysis terminata**	BC ZSM HYM 11092	Germany	09-Jul-2011	SNSB, Zoologische Staatssammlung Muenchen	BOLD:ABY5626	658[0n]
**Chrysis terminata**	BC ZSM HYM 12739	Germany	18-Apr-2011	SNSB, Zoologische Staatssammlung Muenchen	BOLD:ABY5626	658[0n]
**Chrysis terminata**	BC ZSM HYM 12740	Germany	07-Jul-2011	SNSB, Zoologische Staatssammlung Muenchen	BOLD:ABY5626	658[0n]
**Chrysis terminata**	BC ZSM HYM 12741	Germany	07-Jul-2011	SNSB, Zoologische Staatssammlung Muenchen	BOLD:ABY5626	658[0n]
**Chrysis terminata**	GBOL 01977	France	15-Jun-2013	SNSB, Zoologische Staatssammlung Muenchen	BOLD:ABY5626	658[0n]
**Chrysis terminata**	BC-ZSM-HYM-29771-E07	Germany	30-Jul-2017	SNSB, Zoologische Staatssammlung Muenchen	BOLD:ABY5626	658[0n]
**Chrysis terminata**	BC-ZSM-HYM-27533-B10	Germany	06-May-2014	SNSB, Zoologische Staatssammlung Muenchen	BOLD:ABY5626	658[0n]
**Chrysis terminata**	BC-ZSM-HYM-27533-B12	Germany	19-Jun-2014	SNSB, Zoologische Staatssammlung Muenchen	BOLD:ABY5626	658[0n]
**Chrysis vanlithi**	KJ398929	Switzerland	25-Aug-1999	Mined from GenBank, NCBI	BOLD:ACJ4881	655[0n]
**Chrysis viridula**	KY430740.1_tmp	Germany	18-Apr-2011	Private Collection of Oliver Niehuis	BOLD:AAJ0207	675[0n]
**Chrysis viridula**	BC ZSM HYM 07799	France	15-Jul-2010	SNSB, Zoologische Staatssammlung Muenchen	BOLD:AAJ0207	658[0n]
**Chrysis viridula**	BC ZSM HYM 00057	Germany	27-Jun-2006	SNSB, Zoologische Staatssammlung Muenchen	BOLD:AAJ0207	658[0n]
**Chrysis viridula**	BC ZSM HYM 06304	Germany	02-Jul-2008	SNSB, Zoologische Staatssammlung Muenchen	BOLD:AAJ0207	658[0n]
**Chrysis viridula**	BC ZSM HYM	Germany	02-Jul-2008	SNSB, Zoologische	BOLD:AAJ0207	658[0n]

	06305			Staatssammlung Muenchen		
**Chrysura austriaca**	KY430704.1_tmp	Germany	26-Apr-2011	Private Collection of Oliver Niehuis	BOLD:AAJ3472	675[0n]
**Chrysura austriaca**	BC ZSM HYM 09786	Germany	14-Jun-2011	SNSB, Zoologische Staatssammlung Muenchen	BOLD:AAJ3472	658[0n]
**Chrysura austriaca**	BC ZSM HYM 09787	Germany	14-Jun-2011	SNSB, Zoologische Staatssammlung Muenchen	BOLD:AAJ3472	658[0n]
**Chrysura austriaca**	BC ZSM HYM 09788	Germany	14-Jun-2011	SNSB, Zoologische Staatssammlung Muenchen	BOLD:AAJ3472	658[0n]
**Chrysura austriaca**	BC ZSM HYM 09789	Germany	14-Jun-2011	SNSB, Zoologische Staatssammlung Muenchen	BOLD:AAJ3472	658[0n]
**Chrysura austriaca**	BC ZSM HYM 06271	Germany	17-Jun-2002	SNSB, Zoologische Staatssammlung Muenchen	BOLD:AAJ3472	658[0n]
**Chrysura austriaca**	BC ZSM HYM 06272	Germany	30-Jun-2001	SNSB, Zoologische Staatssammlung Muenchen	BOLD:AAJ3472	658[0n]
**Chrysura austriaca**	BC ZSM HYM 06273	Germany	17-Jun-2002	SNSB, Zoologische Staatssammlung Muenchen	BOLD:AAJ3472	658[0n]
**Chrysura austriaca**	BC ZSM HYM 06365	Germany	17-Jun-2002	SNSB, Zoologische Staatssammlung Muenchen	BOLD:AAJ3472	614[0n]
**Chrysura austriaca**	BC ZSM HYM 14960	Germany	30-Jun-2012	SNSB, Zoologische Staatssammlung Muenchen	BOLD:AAJ3472	658[0n]
**Chrysura austriaca**	BC ZSM HYM 14961	Germany	30-Jun-2012	SNSB, Zoologische Staatssammlung Muenchen	BOLD:AAJ3472	658[0n]
**Chrysura austriaca**	BC ZSM HYM 14959	Germany	30-Jun-2012	SNSB, Zoologische Staatssammlung Muenchen	BOLD:AAJ3472	658[0n]
**Chrysura cuprea**	KY430696.1_tmp	Italy	10-May-2011	Private Collection of Oliver Niehuis	BOLD:AAP1055	675[0n]
**Chrysura cuprea**	BC ZSM HYM 06274	Italy	13-May-2006	SNSB, Zoologische Staatssammlung Muenchen	BOLD:AAP1055	658[0n]
**Chrysura cuprea**	BC ZSM HYM 06275	Italy	13-May-2006	SNSB, Zoologische Staatssammlung Muenchen	BOLD:AAP1055	658[0n]
**Chrysura cuprea**	BC ZSM HYM 06276	Italy	13-May-2006	SNSB, Zoologische Staatssammlung Muenchen	BOLD:AAP1055	623[0n]
**Chrysura cuprea**	BC ZSM HYM 06277	Italy	13-May-2006	SNSB, Zoologische Staatssammlung Muenchen	BOLD:AAP1055	658[0n]
**Chrysura cuprea**	BC ZSM HYM 06278	Italy	13-May-2006	SNSB, Zoologische Staatssammlung Muenchen	BOLD:AAP1055	632[0n]
**Chrysura dichroa**	KY430699.1_tmp	Italy	05-May-2011	Private Collection of Oliver Niehuis	BOLD:AFA5928	675[0n]
**Chrysura dichroa**	GBOL04187	Hungary	22-Apr-2014	SNSB, Zoologische Staatssammlung Muenchen	BOLD:AFA5928	658[0n]
**Chrysura dichroa**	GBOL04249	Hungary	22-Apr-2014	SNSB, Zoologische Staatssammlung Muenchen	BOLD:AFA5928	658[0n]
**Chrysura dichroa**	BC ZSM HYM 06279	Italy	13-May-2006	SNSB, Zoologische Staatssammlung Muenchen	BOLD:AAJ3474	602[0n]
**Chrysura dichroa**	BC ZSM HYM 06280	Italy	13-May-2006	SNSB, Zoologische Staatssammlung Muenchen	BOLD:AFA5928	658[0n]

**Chrysura dichroa**	BC ZSM HYM 06281	Italy	13-May-2006	SNSB, Zoologische Staatssammlung Muenchen	BOLD:AFA5928	658[0n]
**Chrysura hirsuta**	KY430697.1_tmp	France	14-Jul-2010	Private Collection of Oliver Niehuis	BOLD:AAY6945	675[0n]
**Chrysura hirsuta**	BC ZSM HYM 07800	France	09-Jul-2010	SNSB, Zoologische Staatssammlung Muenchen	BOLD:AAY6945	658[0n]
**Chrysura hirsuta**	BC ZSM HYM 07801	France	13-Jul-2010	SNSB, Zoologische Staatssammlung Muenchen	BOLD:AAY6945	658[0n]
**Chrysura hirsuta**	BC-ZSM-HYM-27676-D08	Germany	21-Jun-2014	SNSB, Zoologische Staatssammlung Muenchen	BOLD:AAY6945	630[0n]
**Chrysura hybrida**	KY430703.1_tmp	France	12-Jul-2011	Private Collection of Oliver Niehuis	BOLD:AAY6924	675[0n]
**Chrysura hybrida**	BC ZSM HYM 07322	France	15-Jul-2009	SNSB, Zoologische Staatssammlung Muenchen	BOLD:AAY6924	658[0n]
**Chrysura laevigata**	BC ZSM HYM 17478	Italy	20-Jul-2012	SNSB, Zoologische Staatssammlung Muenchen	BOLD:ACG1433	658[0n]
**Chrysura radians**	KY430698.1_tmp	Germany	05-Jun-2010	Private Collection of Oliver Niehuis	BOLD:ABX8519	675[0n]
**Chrysura radians**	BC ZSM HYM 12291	Germany	02-Jun-2009	SNSB, Zoologische Staatssammlung Muenchen	BOLD:ABX8519	658[0n]
**Chrysura rufiventris**	KY430695.1_tmp	Germany	26-Apr-2008	Private Collection of Oliver Niehuis	BOLD:AEC6882	675[0n]
**Chrysura simplex**	KY430705.1_tmp	Italy	03-Jul-1999	Private Collection of Oliver Niehuis	BOLD:AAY6923	675[0n]
**Chrysura simplex**	BC ZSM HYM 07318	France	17-Jul-2009	SNSB, Zoologische Staatssammlung Muenchen	BOLD:AAY6923	632[0n]
**Chrysura simplex**	BC ZSM HYM 07319	France	17-Jul-2009	SNSB, Zoologische Staatssammlung Muenchen	BOLD:AAY6923	627[0n]
**Chrysura simplex**	BC ZSM HYM 07320	France	15-Jul-2009	SNSB, Zoologische Staatssammlung Muenchen	BOLD:AAY6923	658[0n]
**Chrysura simplex**	BC ZSM HYM 07321	Italy	17-Jun-2009	SNSB, Zoologische Staatssammlung Muenchen	BOLD:AAY6923	633[0n]
**Chrysura trimaculata**	KY430694.1_tmp	Germany	24-Apr-2010	Private Collection of Oliver Niehuis	BOLD:AAR9311	675[0n]
**Chrysura trimaculata**	BC ZSM HYM 07878	Germany	06-Jun-2010	SNSB, Zoologische Staatssammlung Muenchen	BOLD:AAR9311	658[0n]
**Chrysura trimaculata**	BC ZSM HYM 07879	Germany	24-Apr-2010	SNSB, Zoologische Staatssammlung Muenchen	BOLD:AAR9311	658[0n]
**Chrysura trimaculata**	BC ZSM HYM 07880	Germany	06-Apr-2010	SNSB, Zoologische Staatssammlung Muenchen	BOLD:AAR9311	658[0n]
**Chrysura trimaculata**	BC ZSM HYM 12764	Germany	11-May-2011	SNSB, Zoologische Staatssammlung Muenchen	BOLD:AAR9311	658[0n]
**Chrysura trimaculata**	BC ZSM HYM 12765	Germany	11-May-2011	SNSB, Zoologische Staatssammlung Muenchen	BOLD:AAR9311	658[0n]
**Chrysura trimaculata**	BC ZSM HYM 12766	Germany	11-Apr-2011	SNSB, Zoologische Staatssammlung Muenchen	BOLD:AAR9311	658[0n]

**Chrysura trimaculata**	BC ZSM HYM 07993	Germany	13-Apr-2009	SNSB, Zoologische Staatssammlung Muenchen	BOLD:AAR9311	658[0n]
**Cleptes nitidulus**	BC ZSM HYM 24682	Germany	07-Jul-2015	SNSB, Zoologische Staatssammlung Muenchen	BOLD:AAZ1160	657[0n]
**Cleptes nitidulus**	BC ZSM HYM 24683	Germany	11-Jun-2015	SNSB, Zoologische Staatssammlung Muenchen	BOLD:AAZ1160	657[0n]
**Cleptes nitidulus**	BC ZSM HYM 24684	Germany	04-Jun-2015	SNSB, Zoologische Staatssammlung Muenchen	BOLD:AAZ1160	657[0n]
**Cleptes nitidulus**	BC ZSM HYM 08002	Germany	16-Aug-2008	SNSB, Zoologische Staatssammlung Muenchen	BOLD:AAZ1160	658[0n]
**Cleptes nitidulus**	BC ZSM HYM 08003	Germany	16-Jul-2009	SNSB, Zoologische Staatssammlung Muenchen	BOLD:AAZ1160	421[0n]
**Cleptes nitidulus**	BC ZSM HYM 08005	Germany	06-Jul-2010	SNSB, Zoologische Staatssammlung Muenchen	BOLD:AAZ1160	421[0n]
**Cleptes semiauratus**	KY430862.1_tmp	Germany	12-Jun-1999	Private Collection of Oliver Niehuis	BOLD:AAJ3895	675[0n]
**Cleptes semiauratus**	BC ZSM HYM 05077	Germany	01-Jul-2008	SNSB, Zoologische Staatssammlung Muenchen	BOLD:AAM4041	658[0n]
**Cleptes semiauratus**	BC ZSM HYM 05078	Germany	14-Aug-2008	SNSB, Zoologische Staatssammlung Muenchen	BOLD:AAM4041	658[0n]
**Cleptes semiauratus**	BC ZSM HYM 05079	Germany	02-Jul-2008	SNSB, Zoologische Staatssammlung Muenchen	BOLD:AAJ3895	658[0n]
**Cleptes semiauratus**	BC ZSM HYM 05080	Germany	02-Jul-2008	SNSB, Zoologische Staatssammlung Muenchen	BOLD:AAJ3895	658[0n]
**Cleptes semiauratus**	BC ZSM HYM 20587	Germany	11-Jun-2011	SNSB, Zoologische Staatssammlung Muenchen	BOLD:AAM4041	664[0n]
**Cleptes semiauratus**	BC ZSM HYM 14123	Germany	14-Apr-2005	SNSB, Zoologische Staatssammlung Muenchen	BOLD:AAM4041	658[0n]
**Cleptes splendidus**	KY430861.1_tmp	Germany	09-Jul-2011	Private Collection of Oliver Niehuis	BOLD:AAR9529	657[0n]
**Cleptes splendidus**	BC ZSM HYM 08004	Germany	07-Jun-2008	SNSB, Zoologische Staatssammlung Muenchen	BOLD:AAR9529	658[0n]
**Cleptes striatipleuris**	KY430863.1_tmp	Germany	23-Jun-2013	Private Collection of Oliver Niehuis	BOLD:AAU2238	675[0n]
**Elampus bidens**	KY430834.1_tmp	Italy	18-Jul-2002	Private Collection of Oliver Niehuis	BOLD:AEC6927	657[0n]
**Elampus constrictus**	BC ZSM HYM 13190	Czech Republic	01-Jul-2008	SNSB, Zoologische Staatssammlung Muenchen	BOLD:ACC2184	658[0n]
**Elampus konowi**	KY430833.1_tmp	Germany	22-May-2011	Private Collection of Oliver Niehuis	BOLD:AAY9882	668[0n]
**Elampus konowi**	BC ZSM HYM 08006	Germany	04-Jun-2010	SNSB, Zoologische Staatssammlung Muenchen	BOLD:AAY9882	633[0n]
**Elampus konowi**	BC ZSM HYM 08007	Germany	04-Jun-2010	SNSB, Zoologische Staatssammlung Muenchen	BOLD:AAY9882	634[1n]
**Elampus konowi**	BC ZSM HYM 08008	Germany	04-Jun-2010	SNSB, Zoologische Staatssammlung Muenchen	BOLD:AAY9882	658[0n]
**Elampus konowi**	BC ZSM HYM 08009	Germany	04-Jun-2010	SNSB, Zoologische Staatssammlung Muenchen	BOLD:AAY9882	658[0n]

**Elampus panzeri**	KY430835.1_tmp	Germany	22-Jun-2014	Private Collection of Oliver Niehuis	BOLD:AEA0923	675[0n]
**Euchroeus purpuratus**	KY430810.1_tmp	Morocco	04-Jul-2002	Private Collection of Oliver Niehuis	BOLD:AED0036	675[0n]
**Hedychridium aereolum**	BC ZSM HYM 07333	France	14-Jul-2009	SNSB, Zoologische Staatssammlung Muenchen	BOLD:AAY6930	624[0n]
**Hedychridium aereolum**	BC ZSM HYM 07334	Italy	11-Jul-2009	SNSB, Zoologische Staatssammlung Muenchen	BOLD:AAY6930	624[0n]
**Hedychridium aereolum**	BC ZSM HYM 07811	France	14-Jul-2010	SNSB, Zoologische Staatssammlung Muenchen	BOLD:AAY6930	606[0n]
**Hedychridium aereolum**	BC ZSM HYM 07812	France	08-Jul-2010	SNSB, Zoologische Staatssammlung Muenchen	BOLD:AAY6930	624[1n]
**Hedychridium ardens**	KY430854.1_tmp	Germany	29-May-2010	Private Collection of Oliver Niehuis	BOLD:AAK4640	675[0n]
**Hedychridium ardens**	BC ZSM HYM 07400	Germany	08-Jun-2008	SNSB, Zoologische Staatssammlung Muenchen	BOLD:AAK4640	627[0n]
**Hedychridium ardens**	BC ZSM HYM 07401	Germany	25-May-2008	SNSB, Zoologische Staatssammlung Muenchen	BOLD:AAK4640	627[6n]
**Hedychridium ardens**	BC ZSM HYM 07402	Germany	03-Jul-2008	SNSB, Zoologische Staatssammlung Muenchen	BOLD:AAK4640	627[0n]
**Hedychridium ardens**	BC ZSM HYM 07403	Germany	30-Jun-2008	SNSB, Zoologische Staatssammlung Muenchen	BOLD:AAK4640	633[0n]
**Hedychridium ardens**	BC ZSM HYM 12776	Germany	15-Jun-2011	SNSB, Zoologische Staatssammlung Muenchen	BOLD:AAK4640	636[0n]
**Hedychridium ardens**	BC ZSM HYM 12777	Germany	18-Aug-2011	SNSB, Zoologische Staatssammlung Muenchen	BOLD:AAK4640	620[0n]
**Hedychridium ardens**	BC ZSM HYM 12778	Germany	11-May-2011	SNSB, Zoologische Staatssammlung Muenchen	BOLD:AAK4640	658[1n]
**Hedychridium ardens**	BC ZSM HYM 09790	Germany	14-Jun-2011	SNSB, Zoologische Staatssammlung Muenchen	BOLD:AAK4640	623[0n]
**Hedychridium ardens**	BC ZSM HYM 09791	Germany	14-Jun-2011	SNSB, Zoologische Staatssammlung Muenchen	BOLD:AAK4640	632[0n]
**Hedychridium ardens**	BC ZSM HYM 09792	Germany	14-Jun-2011	SNSB, Zoologische Staatssammlung Muenchen	BOLD:AAK4640	626[0n]
**Hedychridium ardens**	BC ZSM HYM 09793	Germany	27-Jun-2011	SNSB, Zoologische Staatssammlung Muenchen	BOLD:AAK4640	632[0n]
**Hedychridium ardens**	BC ZSM HYM 09800	Germany	27-Jun-2011	SNSB, Zoologische Staatssammlung Muenchen	BOLD:AAK4640	633[0n]
**Hedychridium ardens**	BC ZSM HYM 08012	Germany	15-Jun-2010	SNSB, Zoologische Staatssammlung Muenchen	BOLD:AAK4640	631[0n]
**Hedychridium ardens**	BC ZSM HYM 12954	Germany	28-Jun-2004	SNSB, Zoologische Staatssammlung Muenchen	BOLD:AAK4640	565[0n]
**Hedychridium ardens**	BC ZSM HYM 14966	Germany	19-Jun-2012	SNSB, Zoologische Staatssammlung Muenchen	BOLD:AAK4640	636[0n]
**Hedychridium ardens**	BC ZSM HYM 08010	Germany	19-Aug-2010	SNSB, Zoologische Staatssammlung Muenchen	BOLD:AAK4640	642[0n]

**Hedychridium ardens**	BC ZSM HYM 08011	Germany	01-Jul-2010	SNSB, Zoologische Staatssammlung Muenchen	BOLD:AAK4640	636[0n]
**Hedychridium ardens**	BC ZSM HYM 08013	Germany	14-Aug-2010	SNSB, Zoologische Staatssammlung Muenchen	BOLD:AAK4640	637[2n]
**Hedychridium caputaureum**	KY430845.1_tmp	Germany	21-Jun-2012	Private Collection of Oliver Niehuis	BOLD:AAU0775	675[0n]
**Hedychridium caputaureum**	BC ZSM HYM 14964	Germany	19-Jun-2012	SNSB, Zoologische Staatssammlung Muenchen	BOLD:AAU0775	658[0n]
**Hedychridium caputaureum**	GBOL19242	Germany	25-Jun-2016	SNSB, Zoologische Staatssammlung Muenchen	BOLD:AAU0775	454[0n]
**Hedychridium coriaceum**	KY430841.1_tmp	Germany	24-Jul-2012	Private Collection of Oliver Niehuis	BOLD:AAY6943	675[0n]
**Hedychridium coriaceum**	BC ZSM HYM 07404	Germany	02-Jul-2009	SNSB, Zoologische Staatssammlung Muenchen	BOLD:AAY6943	658[0n]
**Hedychridium coriaceum**	BC ZSM HYM 07405	Germany	22-Jul-2009	SNSB, Zoologische Staatssammlung Muenchen	BOLD:AAY6943	658[0n]
**Hedychridium coriaceum**	BC ZSM HYM 11085	Germany	09-Jul-2011	SNSB, Zoologische Staatssammlung Muenchen	BOLD:AAY6943	658[0n]
**Hedychridium coriaceum**	BC ZSM HYM 14965	Germany	19-Jun-2012	SNSB, Zoologische Staatssammlung Muenchen	BOLD:AAY6943	658[0n]
**Hedychridium coriaceum**	BC ZSM HYM 08014	Germany	21-Aug-2009	SNSB, Zoologische Staatssammlung Muenchen	BOLD:AAY6943	658[0n]
**Hedychridium coriaceum**	BC ZSM HYM 08015	Germany	19-Aug-2010	SNSB, Zoologische Staatssammlung Muenchen	BOLD:AAY6943	658[0n]
**Hedychridium coriaceum**	BC ZSM HYM 08016	Germany	14-Aug-2010	SNSB, Zoologische Staatssammlung Muenchen	BOLD:AAY6943	658[0n]
**Hedychridium coriaceum**	BC ZSM HYM 08017	Germany	25-Aug-2010	SNSB, Zoologische Staatssammlung Muenchen	BOLD:AAY6943	633[0n]
**Hedychridium cupratum**	BC ZSM HYM 07809	France	14-Jul-2010	SNSB, Zoologische Staatssammlung Muenchen	BOLD:AAY6946	658[0n]
**Hedychridium cupratum**	BC ZSM HYM 07810	France	14-Jul-2010	SNSB, Zoologische Staatssammlung Muenchen	BOLD:AAY6946	631[0n]
**Hedychridium cupratum**	BC ZSM HYM 17342	Italy	20-Jul-2012	SNSB, Zoologische Staatssammlung Muenchen	BOLD:AAY6946	658[0n]
**Hedychridium cupreum**	KY430843.1_tmp	Germany	29-May-2010	Private Collection of Oliver Niehuis	BOLD:AAY9838	675[0n]
**Hedychridium cupreum**	BC ZSM HYM 08018	Germany	25-May-2010	SNSB, Zoologische Staatssammlung Muenchen	BOLD:AAY9838	658[0n]
**Hedychridium cupreum**	BC ZSM HYM 08019	Germany	15-May-2010	SNSB, Zoologische Staatssammlung Muenchen	BOLD:AAY9838	633[0n]
**Hedychridium cupreum**	BC ZSM HYM 08020	Germany	29-May-2010	SNSB, Zoologische Staatssammlung Muenchen	BOLD:AAY9838	658[0n]
**Hedychridium cupreum**	BC ZSM HYM 08021	Germany	29-May-2010	SNSB, Zoologische Staatssammlung Muenchen	BOLD:AAY9838	658[0n]
**Hedychridium elegantulum**	KY430846.1_tmp	Germany	28-Jun-2009	Private Collection of Oliver Niehuis	BOLD:AAY9837	675[0n]
**Hedychridium elegantulum**	BC ZSM HYM 08022	Germany	26-Jul-2006	SNSB, Zoologische Staatssammlung Muenchen	BOLD:AAY9837	658[0n]

**Hedychridium elegantulum**	BC ZSM HYM 08023	Germany	26-Jul-2006	SNSB, Zoologische Staatssammlung Muenchen	BOLD:AAY9837	658[0n]
**Hedychridium elegantulum**	BC ZSM HYM 08024	Germany	06-Jul-2006	SNSB, Zoologische Staatssammlung Muenchen	BOLD:AAY9837	658[0n]
**Hedychridium elegantulum**	BC ZSM HYM 08025	Germany	06-Jul-2006	SNSB, Zoologische Staatssammlung Muenchen	BOLD:AAY9837	658[0n]
**Hedychridium femoratum**	BC-ZSM-HYM-23654-F02	Slovakia	06-Aug-2008	SNSB, Zoologische Staatssammlung Muenchen	BOLD:AAV6886	601[0n]
**Hedychridium jucundum**	BC-ZSM-HYM-23654-F10	Slovakia	06-Aug-2008	SNSB, Zoologische Staatssammlung Muenchen	BOLD:AAU1479	658[0n]
**Hedychridium jucundum**	BC ZSM HYM 13200	Czech Republic	29-Jul-2008	SNSB, Zoologische Staatssammlung Muenchen	BOLD:AAU1479	658[0n]
**Hedychridium krajniki**	KY430842.1_tmp	Germany	21-Jun-2012	Private Collection of Oliver Niehuis	BOLD:AAZ0056	661[0n]
**Hedychridium krajniki**	BC ZSM HYM 08029	Germany	15-Jul-2008	SNSB, Zoologische Staatssammlung Muenchen	BOLD:AAZ0056	658[0n]
**Hedychridium krajniki**	BC ZSM HYM 13191	Czech Republic	29-Jul-2008	SNSB, Zoologische Staatssammlung Muenchen	BOLD:AAZ0056	658[0n]
**Hedychridium krajniki**	BC ZSM HYM 08026	Germany	19-Aug-2010	SNSB, Zoologische Staatssammlung Muenchen	BOLD:AAZ0056	658[0n]
**Hedychridium krajniki**	BC ZSM HYM 08027	Germany	05-Aug-2009	SNSB, Zoologische Staatssammlung Muenchen	BOLD:AAZ0056	658[0n]
**Hedychridium monochroum**	KY430850.1_tmp	Italy	18-Jun-2011	Private Collection of Oliver Niehuis	BOLD:AAY1978	675[0n]
**Hedychridium niemelai**	BC ZSM HYM 12772	Germany	27-Jul-2010	SNSB, Zoologische Staatssammlung Muenchen	BOLD:AAU1294	658[0n]
**Hedychridium niemelai**	BC ZSM HYM 12775	Germany	07-Jul-2011	SNSB, Zoologische Staatssammlung Muenchen	BOLD:AAU1294	658[0n]
**Hedychridium purpurascens**	ZSM-HYM-29808-F11	Germany	04-Aug-2021	SNSB, Zoologische Staatssammlung Muenchen	BOLD:AEP5116	658[0n]
**Hedychridium roseum**	KY430844.1_tmp	Germany	22-May-2011	Private Collection of Oliver Niehuis	BOLD:AAE3259	675[0n]
**Hedychridium roseum**	BC ZSM HYM 07335	Italy	10-Jul-2009	SNSB, Zoologische Staatssammlung Muenchen	BOLD:AAE3259	658[0n]
**Hedychridium roseum**	BC ZSM HYM 07846	Germany	31-Jul-2009	SNSB, Zoologische Staatssammlung Muenchen	BOLD:AAE3259	658[0n]
**Hedychridium roseum**	BC ZSM HYM 07847	Germany	14-Aug-2010	SNSB, Zoologische Staatssammlung Muenchen	BOLD:AAE3259	658[0n]
**Hedychridium roseum**	BC ZSM HYM 07848	Germany	05-Jul-2006	SNSB, Zoologische Staatssammlung Muenchen	BOLD:AAE3259	658[0n]
**Hedychridium roseum**	BC ZSM HYM 07849	Germany	06-Jun-2008	SNSB, Zoologische Staatssammlung Muenchen	BOLD:AAE3259	658[0n]
**Hedychridium roseum**	BC ZSM HYM 09801	Germany	27-Jun-2011	SNSB, Zoologische Staatssammlung Muenchen	BOLD:ACG6690	658[0n]
**Hedychridium roseum**	BC ZSM HYM 11109	Italy	17-Jul-2001	SNSB, Zoologische Staatssammlung Muenchen	BOLD:AAE3259	658[0n]

**Hedychridium roseum**	BC ZSM HYM 19917	Germany	07-Jul-2013	SNSB, Zoologische Staatssammlung Muenchen	BOLD:ACG6690	658[0n]
**Hedychridium roseum**	BC ZSM HYM 19918	Germany	17-Jul-2011	SNSB, Zoologische Staatssammlung Muenchen	BOLD:AAE3259	658[0n]
**Hedychridium roseum**	BC ZSM HYM 19921	Italy	08-Jun-2007	SNSB, Zoologische Staatssammlung Muenchen	BOLD:AAE3260	658[0n]
**Hedychridium roseum**	GBOL19245	Germany	08-Jun-2016	SNSB, Zoologische Staatssammlung Muenchen	BOLD:ACG6690	475[0n]
**Hedychridium roseum**	BC ZSM HYM 21987	Germany	02-Jul-2014	SNSB, Zoologische Staatssammlung Muenchen	BOLD:ACG6690	658[0n]
**Hedychridium rossicum**	BC ZSM HYM 07850	Germany	15-Aug-2009	SNSB, Zoologische Staatssammlung Muenchen	BOLD:AAE3258	658[0n]
**Hedychridium rossicum**	BC ZSM HYM 07851	Germany	07-Jul-2010	SNSB, Zoologische Staatssammlung Muenchen	BOLD:AAE3258	658[0n]
**Hedychridium rossicum**	BC ZSM HYM 07852	Germany	02-Jul-2008	SNSB, Zoologische Staatssammlung Muenchen	BOLD:AAE3258	658[0n]
**Hedychridium rossicum**	BC ZSM HYM 07853	Germany	07-Jun-2008	SNSB, Zoologische Staatssammlung Muenchen	BOLD:AAE3258	658[0n]
**Hedychridium rossicum**	BC ZSM HYM 12760	Germany	10-Jul-2011	SNSB, Zoologische Staatssammlung Muenchen	BOLD:AAE3258	627[0n]
**Hedychridium rossicum**	BC ZSM HYM 12761	Germany	14-Aug-2000	SNSB, Zoologische Staatssammlung Muenchen	BOLD:AAE3258	658[0n]
**Hedychridium rossicum**	BC ZSM HYM 12762	Germany	04-Jul-2005	SNSB, Zoologische Staatssammlung Muenchen	BOLD:AAE3258	658[0n]
**Hedychridium rossicum**	BC ZSM HYM 12763	Germany	04-Jul-2005	SNSB, Zoologische Staatssammlung Muenchen	BOLD:AAE3258	658[0n]
**Hedychridium rossicum**	BC ZSM HYM 24678	Germany	30-Jun-2015	SNSB, Zoologische Staatssammlung Muenchen	BOLD:AAE3258	658[0n]
**Hedychridium zelleri**	BC ZSM HYM 18332	Germany	27-Jun-2011	SNSB, Zoologische Staatssammlung Muenchen	BOLD:ACG7149	630[0n]
**Hedychridium zelleri**	BC ZSM HYM 18334	Germany	27-Jun-2011	SNSB, Zoologische Staatssammlung Muenchen	BOLD:ACG7149	604[0n]
**Hedychridium zelleri**	BC ZSM HYM 18335	Germany	27-Jun-2011	SNSB, Zoologische Staatssammlung Muenchen	BOLD:ACG7149	609[0n]
**Hedychrum chalybaeum**	BC ZSM HYM 07399	Germany	22-Jul-2009	SNSB, Zoologische Staatssammlung Muenchen	BOLD:AAY6942	658[0n]
**Hedychrum chalybaeum**	BC ZSM HYM 07854	Germany	15-Jul-2009	SNSB, Zoologische Staatssammlung Muenchen	BOLD:AAY6942	658[0n]
**Hedychrum chalybaeum**	BC ZSM HYM 07855	Germany	15-Jul-2009	SNSB, Zoologische Staatssammlung Muenchen	BOLD:AAY6942	658[0n]
**Hedychrum chalybaeum**	BC ZSM HYM 07856	Germany	21-Jul-2009	SNSB, Zoologische Staatssammlung Muenchen	BOLD:AAY6942	658[0n]
**Hedychrum chalybaeum**	BC ZSM HYM 07857	Germany	15-Jul-2009	SNSB, Zoologische Staatssammlung Muenchen	BOLD:AAY6942	658[0n]
**Hedychrum chalybaeum**	BC ZSM HYM 09794	Germany	22-Jul-2009	SNSB, Zoologische Staatssammlung Muenchen	BOLD:AAY6942	658[0n]
**Hedychrum chalybaeum**	BC ZSM HYM 11077	Germany	16-Jul-2011	SNSB, Zoologische Staatssammlung Muenchen	BOLD:AAY6942	658[0n]

**Hedychrum chalybaeum**	BC ZSM HYM 17329	Germany	18-Jul-2012	SNSB, Zoologische Staatssammlung Muenchen	BOLD:AAY6942	658[0n]
**Hedychrum chalybaeum**	BC ZSM HYM 17330	Germany	18-Jul-2012	SNSB, Zoologische Staatssammlung Muenchen	BOLD:AAY6942	658[0n]
**Hedychrum chalybaeum**	BC ZSM HYM 17331	Germany	18-Jul-2012	SNSB, Zoologische Staatssammlung Muenchen	BOLD:AAY6942	658[0n]
**Hedychrum gerstaeckeri**	KY430856.1_tmp	Germany	24-Jul-2012	Private Collection of Oliver Niehuis	BOLD:AAM3758	668[1n]
**Hedychrum gerstaeckeri**	BC ZSM HYM 07881	Germany	15-Jul-2009	SNSB, Zoologische Staatssammlung Muenchen	BOLD:AAM3758	658[0n]
**Hedychrum gerstaeckeri**	BC ZSM HYM 17332	Germany	18-Jul-2012	SNSB, Zoologische Staatssammlung Muenchen	BOLD:AAM3758	658[0n]
**Hedychrum gerstaeckeri**	BC ZSM HYM 17335	Germany	18-Jul-2012	SNSB, Zoologische Staatssammlung Muenchen	BOLD:AAM3758	658[0n]
**Hedychrum gerstaeckeri**	BC ZSM HYM 05069	Germany	24-Jul-2008	SNSB, Zoologische Staatssammlung Muenchen	BOLD:AAM3758	658[0n]
**Hedychrum gerstaeckeri**	BC ZSM HYM 05070	Germany	08-Jun-2008	SNSB, Zoologische Staatssammlung Muenchen	BOLD:AAM3758	658[0n]
**Hedychrum gerstaeckeri**	BC ZSM HYM 05071	Germany	19-Jul-2008	SNSB, Zoologische Staatssammlung Muenchen	BOLD:AAM3758	658[0n]
**Hedychrum gerstaeckeri**	BC ZSM HYM 05072	Germany	24-Jul-2008	SNSB, Zoologische Staatssammlung Muenchen	BOLD:AAM3758	658[0n]
**Hedychrum gerstaeckeri**	BC ZSM HYM 14067	Germany	16-Jul-2011	SNSB, Zoologische Staatssammlung Muenchen	BOLD:AAM3758	658[0n]
**Hedychrum gerstaeckeri**	BC ZSM HYM 14068	Germany	16-Jul-2011	SNSB, Zoologische Staatssammlung Muenchen	BOLD:AAM3758	658[0n]
**Hedychrum gerstaeckeri**	BC ZSM HYM 14069	Germany	16-Jul-2011	SNSB, Zoologische Staatssammlung Muenchen	BOLD:AAM3758	658[0n]
**Hedychrum gerstaeckeri**	BC ZSM HYM 14070	Germany	16-Jul-2011	SNSB, Zoologische Staatssammlung Muenchen	BOLD:AAM3758	658[0n]
**Hedychrum gerstaeckeri**	BC ZSM HYM 14111	Germany	22-Jul-2009	SNSB, Zoologische Staatssammlung Muenchen	BOLD:AAM3758	658[0n]
**Hedychrum gerstaeckeri**	BC ZSM HYM 14112	Germany	22-Jul-2009	SNSB, Zoologische Staatssammlung Muenchen	BOLD:AAM3758	658[0n]
**Hedychrum gerstaeckeri**	BC ZSM HYM 14113	Germany	02-Jul-2009	SNSB, Zoologische Staatssammlung Muenchen	BOLD:AAM3758	658[0n]
**Hedychrum gerstaeckeri**	BC ZSM HYM 14114	Germany	22-Jul-2009	SNSB, Zoologische Staatssammlung Muenchen	BOLD:AAM3758	658[0n]
**Hedychrum gerstaeckeri**	BC ZSM HYM 14118	Germany	27-Jul-2010	SNSB, Zoologische Staatssammlung Muenchen	BOLD:AAM3758	658[0n]
**Hedychrum longicolle**	KY430857.1_tmp	France	14-Jul-2011	Private Collection of Oliver Niehuis	BOLD:AED0972	675[0n]
**Hedychrum niemelai**	KY430859.1_tmp	Germany	25-Jul-2012	Private Collection of Oliver Niehuis		675[10n]
**Hedychrum niemelai**	BC ZSM HYM 07346	Italy	08-Jun-2007	SNSB, Zoologische Staatssammlung Muenchen	BOLD:AAU1294	658[0n]

**Hedychrum niemelai**	BC ZSM HYM 07383	Germany	22-Jul-2009	SNSB, Zoologische Staatssammlung Muenchen	BOLD:AAU1294	658[0n]
**Hedychrum niemelai**	BC ZSM HYM 07384	Germany	22-Jul-2009	SNSB, Zoologische Staatssammlung Muenchen	BOLD:AAU1294	658[0n]
**Hedychrum niemelai**	BC ZSM HYM 07385	Germany	22-Jul-2009	SNSB, Zoologische Staatssammlung Muenchen	BOLD:AAU1294	658[0n]
**Hedychrum niemelai**	BC ZSM HYM 07858	Germany	19-Jun-2008	SNSB, Zoologische Staatssammlung Muenchen	BOLD:AAU1294	658[0n]
**Hedychrum niemelai**	BC ZSM HYM 07859	Germany	24-Jul-2010	SNSB, Zoologische Staatssammlung Muenchen	BOLD:AAU1294	658[0n]
**Hedychrum niemelai**	BC ZSM HYM 07882	Germany	12-Jun-2009	SNSB, Zoologische Staatssammlung Muenchen	BOLD:AAU1294	658[0n]
**Hedychrum niemelai**	BC ZSM HYM 12773	Germany	27-Jul-2010	SNSB, Zoologische Staatssammlung Muenchen	BOLD:AAU1294	658[0n]
**Hedychrum niemelai**	BC ZSM HYM 12774	Germany	27-Jul-2010	SNSB, Zoologische Staatssammlung Muenchen	BOLD:AAU1294	658[0n]
**Hedychrum niemelai**	BC ZSM HYM 09795	Germany	22-Jul-2009	SNSB, Zoologische Staatssammlung Muenchen	BOLD:AAU1294	658[0n]
**Hedychrum niemelai**	BC ZSM HYM 09796	Germany	22-Jul-2009	SNSB, Zoologische Staatssammlung Muenchen	BOLD:AAU1294	658[0n]
**Hedychrum niemelai**	BC ZSM HYM 11074	Germany	09-Jul-2011	SNSB, Zoologische Staatssammlung Muenchen	BOLD:AAU1294	658[0n]
**Hedychrum niemelai**	BC ZSM HYM 11075	Germany	09-Jul-2011	SNSB, Zoologische Staatssammlung Muenchen	BOLD:AAU1294	658[0n]
**Hedychrum niemelai**	BC ZSM HYM 11076	Germany	09-Jul-2011	SNSB, Zoologische Staatssammlung Muenchen	BOLD:AAU1294	658[0n]
**Hedychrum niemelai**	BC ZSM HYM 17333	Germany	18-Jul-2012	SNSB, Zoologische Staatssammlung Muenchen	BOLD:AAU1294	658[0n]
**Hedychrum niemelai**	BC ZSM HYM 21080	Germany	17-Jul-2012	SNSB, Zoologische Staatssammlung Muenchen	BOLD:AAU1294	658[0n]
**Hedychrum niemelai**	BC ZSM HYM 14105	Germany	16-Jul-2011	SNSB, Zoologische Staatssammlung Muenchen	BOLD:AAU1294	658[0n]
**Hedychrum niemelai**	BC ZSM HYM 14115	Germany	22-Jul-2009	SNSB, Zoologische Staatssammlung Muenchen	BOLD:AAU1294	658[0n]
**Hedychrum niemelai**	BC ZSM HYM 14117	Germany	22-Jul-2009	SNSB, Zoologische Staatssammlung Muenchen	BOLD:AAU1294	658[0n]
**Hedychrum niemelai**	BC ZSM HYM 14119	Germany	09-Jul-2011	SNSB, Zoologische Staatssammlung Muenchen	BOLD:AAU1294	658[0n]
**Hedychrum niemelai**	BC ZSM HYM 14120	Germany	09-Jul-2011	SNSB, Zoologische Staatssammlung Muenchen	BOLD:AAU1294	658[0n]
**Hedychrum niemelai**	BC ZSM HYM 14121	Germany	09-Jul-2011	SNSB, Zoologische Staatssammlung Muenchen	BOLD:AAU1294	658[0n]
**Hedychrum niemelai**	BC ZSM HYM 19920	Germany	18-Jul-2012	SNSB, Zoologische Staatssammlung Muenchen	BOLD:AAU1294	658[0n]
**Hedychrum nobile**	KY430860.2_tmp	Germany	13-Jun-2009	Private Collection of Oliver Niehuis	BOLD:AAK4644	675[1n]
**Hedychrum nobile**	BC ZSM HYM 05062	Germany	19-Jul-2008	SNSB, Zoologische Staatssammlung Muenchen	BOLD:AAK4644	658[0n]

**Hedychrum nobile**	BC ZSM HYM 00059	Germany	08-Jun-2008	SNSB, Zoologische Staatssammlung Muenchen	BOLD:AAK4644	658[0n]
**Hedychrum nobile**	BC ZSM HYM 17334	Italy	20-Jul-2012	SNSB, Zoologische Staatssammlung Muenchen	BOLD:AAK4644	658[0n]
**Hedychrum nobile**	BC ZSM HYM 17336	Germany	18-Jul-2012	SNSB, Zoologische Staatssammlung Muenchen	BOLD:AAK4644	658[0n]
**Hedychrum nobile**	BC ZSM HYM 05061	Germany	19-Jul-2008	SNSB, Zoologische Staatssammlung Muenchen	BOLD:AAK4644	658[0n]
**Hedychrum nobile**	BC ZSM HYM 05063	Germany	30-Jun-2008	SNSB, Zoologische Staatssammlung Muenchen	BOLD:AAK4644	658[0n]
**Hedychrum nobile**	BC ZSM HYM 05064	Germany	08-Jun-2008	SNSB, Zoologische Staatssammlung Muenchen	BOLD:AAK4644	658[0n]
**Hedychrum nobile**	BC ZSM HYM 14107	Germany	27-Jun-2011	SNSB, Zoologische Staatssammlung Muenchen	BOLD:AAK4644	658[0n]
**Hedychrum nobile**	BC ZSM HYM 14108	Germany	27-Jun-2011	SNSB, Zoologische Staatssammlung Muenchen	BOLD:AAK4644	658[0n]
**Hedychrum nobile**	BC ZSM HYM 14109	Germany	27-Jun-2011	SNSB, Zoologische Staatssammlung Muenchen	BOLD:AAK4644	658[0n]
**Hedychrum nobile**	BC ZSM HYM 14110	Germany	27-Jun-2011	SNSB, Zoologische Staatssammlung Muenchen	BOLD:AAK4644	658[0n]
**Hedychrum nobile**	BC ZSM HYM 14116	Germany	26-Jul-2010	SNSB, Zoologische Staatssammlung Muenchen	BOLD:AAK4644	658[0n]
**Hedychrum rutilans**	BC ZSM HYM 17338	Italy	20-Jul-2012	SNSB, Zoologische Staatssammlung Muenchen	BOLD:AAK4643	658[0n]
**Hedychrum rutilans**	BC ZSM HYM 17339	Italy	20-Jul-2012	SNSB, Zoologische Staatssammlung Muenchen	BOLD:AAK4643	658[0n]
**Hedychrum rutilans**	BC ZSM HYM 05065	Germany	03-Jul-2008	SNSB, Zoologische Staatssammlung Muenchen	BOLD:AAK4643	658[0n]
**Hedychrum rutilans**	BC ZSM HYM 05067	Germany	15-Jul-2008	SNSB, Zoologische Staatssammlung Muenchen	BOLD:AAK4643	658[0n]
**Hedychrum rutilans**	BC ZSM HYM 05068	Germany	22-Aug-2008	SNSB, Zoologische Staatssammlung Muenchen	BOLD:AAK4643	658[0n]
**Hedychrum rutilans**	BC ZSM HYM 14102	Germany	22-Jul-2009	SNSB, Zoologische Staatssammlung Muenchen	BOLD:AAK4643	658[0n]
**Hedychrum rutilans**	BC ZSM HYM 14103	Germany	27-Jun-2011	SNSB, Zoologische Staatssammlung Muenchen	BOLD:AAK4643	658[0n]
**Hedychrum rutilans**	BC ZSM HYM 14104	Germany	27-Jun-2011	SNSB, Zoologische Staatssammlung Muenchen	BOLD:AAK4643	658[0n]
**Hedychrum rutilans**	BC ZSM HYM 14950	Germany	02-Jul-2012	SNSB, Zoologische Staatssammlung Muenchen	BOLD:AAK4643	658[0n]
**Hedychrum rutilans**	BC ZSM HYM 14951	Germany	02-Jul-2012	SNSB, Zoologische Staatssammlung Muenchen	BOLD:AAK4643	658[0n]
**Hedychrum viridiaureum**	KY430858.2_tmp	Germany	24-Jul-2012	Private Collection of Oliver Niehuis	BOLD:AAM3491	675[0n]
**Hedychrum viridiaureum**	BC ZSM HYM 11078	Germany	16-Jul-2011	SNSB, Zoologische Staatssammlung Muenchen	BOLD:AAM3491	658[0n]

**Hedychrum viridiaureum**	BC ZSM HYM 11079	Germany	16-Jul-2011	SNSB, Zoologische Staatssammlung Muenchen	BOLD:AAM3491	658[0n]
**Hedychrum viridiaureum**	BC ZSM HYM 17340	Germany	18-Jul-2012	SNSB, Zoologische Staatssammlung Muenchen	BOLD:AAM3491	658[0n]
**Hedychrum viridiaureum**	BC ZSM HYM 17341	Germany	18-Jul-2012	SNSB, Zoologische Staatssammlung Muenchen	BOLD:AAM3491	658[0n]
**Hedychrum viridiaureum**	BC ZSM HYM 05066	Germany	22-Jun-2008	SNSB, Zoologische Staatssammlung Muenchen	BOLD:AAM3491	658[0n]
**Hedychrum viridiaureum**	BC ZSM HYM 14106	Germany	16-Jul-2011	SNSB, Zoologische Staatssammlung Muenchen	BOLD:AAM3491	658[0n]
**Holopyga australis**	KY430840.1_tmp	Germany	04-May-2014	Private Collection of Oliver Niehuis	BOLD:ABX5007	675[0n]
**Holopyga australis**	BC ZSM HYM 07883	Germany	06-Jun-2010	SNSB, Zoologische Staatssammlung Muenchen	BOLD:ABX5007	658[0n]
**Holopyga australis**	BC ZSM HYM 07884	Germany	06-Jun-2010	SNSB, Zoologische Staatssammlung Muenchen	BOLD:ABX5007	658[0n]
**Holopyga australis**	BC ZSM HYM 07885	Germany	25-May-2010	SNSB, Zoologische Staatssammlung Muenchen	BOLD:ABX5007	658[0n]
**Holopyga australis**	BC ZSM HYM 07981	Germany	02-Jun-2010	SNSB, Zoologische Staatssammlung Muenchen	BOLD:ABX5007	658[0n]
**Holopyga chrysonota**	KY430837.1_tmp	Germany	02-Jun-2011	Private Collection of Oliver Niehuis	BOLD:AAY9689	675[0n]
**Holopyga chrysonota**	BC ZSM HYM 08031	Germany	28-Jul-2006	SNSB, Zoologische Staatssammlung Muenchen	BOLD:AAY9689	658[0n]
**Holopyga chrysonota**	BC ZSM HYM 08032	Germany	07-Jun-2008	SNSB, Zoologische Staatssammlung Muenchen	BOLD:AAY9689	658[0n]
**Holopyga chrysonota**	BC ZSM HYM 08033	Germany	14-Jun-2010	SNSB, Zoologische Staatssammlung Muenchen	BOLD:AAY9689	658[0n]
**Holopyga chrysonota**	BC ZSM HYM 08034	Germany	10-Jun-2010	SNSB, Zoologische Staatssammlung Muenchen	BOLD:AAY9689	658[0n]
**Holopyga chrysonota**	BC ZSM HYM 17470	Italy	20-Jul-2012	SNSB, Zoologische Staatssammlung Muenchen	BOLD:AAY9689	658[0n]
**Holopyga chrysonota**	BC ZSM HYM 19900	Germany	08-Jul-2013	SNSB, Zoologische Staatssammlung Muenchen	BOLD:AAV7063	658[0n]
**Holopyga chrysonota**	BC ZSM HYM 07989	Germany	07-Jun-2008	SNSB, Zoologische Staatssammlung Muenchen	BOLD:AAY9689	632[0n]
**Holopyga fervida**	BC ZSM HYM 11082	Germany	16-Jul-2011	SNSB, Zoologische Staatssammlung Muenchen	BOLD:ACV6331	658[0n]
**Holopyga fervida**	BC ZSM HYM 11090	Germany	16-Jul-2011	SNSB, Zoologische Staatssammlung Muenchen	BOLD:ACV6331	658[0n]
**Holopyga fervida**	BC ZSM HYM 14963	Germany	19-Jun-2012	SNSB, Zoologische Staatssammlung Muenchen	BOLD:AAX1104	625[0n]
**Holopyga fervida**	BC ZSM HYM 07982	Spain	22-Jun-2010	SNSB, Zoologische Staatssammlung Muenchen	BOLD:AAY9735	658[0n]
**Holopyga fervida**	BC ZSM HYM 07983	Spain	22-Jun-2010	SNSB, Zoologische Staatssammlung Muenchen	BOLD:AAY9735	658[0n]
**Holopyga fervida**	BC ZSM HYM 07984	Germany	13-Jun-2006	SNSB, Zoologische Staatssammlung Muenchen	BOLD:AAY9735	658[0n]

**Holopyga fervida**	BC ZSM HYM 07985	Spain	22-Jun-2010	SNSB, Zoologische Staatssammlung Muenchen	BOLD:AAY9735	658[0n]
**Holopyga generosa**	KY430838.1_tmp	Germany	03-Jul-2010	Private Collection of Oliver Niehuis	BOLD:AAY6928	675[0n]
**Holopyga generosa**	BC ZSM HYM 07326	France	12-Jul-2009	SNSB, Zoologische Staatssammlung Muenchen	BOLD:AAY6927	618[1n]
**Holopyga generosa**	BC ZSM HYM 07327	France	12-Jul-2009	SNSB, Zoologische Staatssammlung Muenchen	BOLD:AAY6928	584[0n]
**Holopyga generosa**	BC ZSM HYM 07328	France	12-Jul-2009	SNSB, Zoologische Staatssammlung Muenchen	BOLD:AAY6928	538[0n]
**Holopyga generosa**	BC ZSM HYM 07329	France	12-Jul-2009	SNSB, Zoologische Staatssammlung Muenchen	BOLD:AAY6927	658[0n]
**Holopyga generosa**	BC ZSM HYM 12768	Germany	27-Jul-2010	SNSB, Zoologische Staatssammlung Muenchen	BOLD:ACC3318	658[0n]
**Holopyga generosa**	BC ZSM HYM 12769	Germany	27-Jul-2010	SNSB, Zoologische Staatssammlung Muenchen	BOLD:ACC3318	658[0n]
**Holopyga generosa**	BC ZSM HYM 12770	Germany	05-Jun-2011	SNSB, Zoologische Staatssammlung Muenchen	BOLD:ACC3318	627[0n]
**Holopyga generosa**	BC ZSM HYM 17297	Italy	20-Jul-2012	SNSB, Zoologische Staatssammlung Muenchen	BOLD:ACC3318	658[0n]
**Holopyga generosa**	BC ZSM HYM 24681	Germany	07-Jul-2015	SNSB, Zoologische Staatssammlung Muenchen	BOLD:ACC3318	658[0n]
**Holopyga generosa**	BC ZSM HYM 13197	Czech Republic	29-May-2005	SNSB, Zoologische Staatssammlung Muenchen	BOLD:ACC3318	658[0n]
**Holopyga generosa**	BC ZSM HYM 21988	Germany	23-Jul-2014	SNSB, Zoologische Staatssammlung Muenchen	BOLD:ACC3318	658[0n]
**Holopyga generosa**	BC ZSM HYM 07986	Germany	14-Aug-2010	SNSB, Zoologische Staatssammlung Muenchen	BOLD:AAY6928	604[0n]
**Holopyga generosa**	BC ZSM HYM 07987	Germany	08-Jul-2010	SNSB, Zoologische Staatssammlung Muenchen	BOLD:AAY6928	658[5n]
**Holopyga generosa**	BC ZSM HYM 07988	Germany	09-Jun-2009	SNSB, Zoologische Staatssammlung Muenchen	BOLD:AAZ6194	658[0n]
**Holopyga generosa**	BC ZSM HYM 21989	Germany	02-Jul-2014	SNSB, Zoologische Staatssammlung Muenchen	BOLD:ACC3318	658[0n]
**Holopyga minuma**	BC ZSM HYM 13194	Slovakia	06-Aug-2008	SNSB, Zoologische Staatssammlung Muenchen	BOLD:AAV7065	658[0n]
**Holopyga similis**	KY430836.1_tmp	Germany	09-Jul-1999	Private Collection of Oliver Niehuis	BOLD:AED0274	675[0n]
**Omalus aeneus**	KY430827.1_tmp	Hungary	24-Sep-2007	Private Collection of Oliver Niehuis	BOLD:ACQ9469	675[0n]
**Omalus aeneus**	BC ZSM HYM 12915	Italy	04-Aug-2011	SNSB, Zoologische Staatssammlung Muenchen	BOLD:ACC4462	658[0n]
**Omalus aeneus**	BC-ZSM-HYM-23654-E05	Slovakia	05-Aug-2008	SNSB, Zoologische Staatssammlung Muenchen	BOLD:ACG9650	658[0n]
**Omalus biaccinctus**	KY430828.1_tmp	Germany	21-Jun-2012	Private Collection of Oliver Niehuis	BOLD:AFA2340	675[0n]

**Omalus biaccinctus**	BC ZSM HYM 24679	Germany	11-Jun-2015	SNSB, Zoologische Staatssammlung Muenchen	BOLD:AFA2340	658[0n]
**Omalus puncticollis**	KY430826.1_tmp	Germany	21-Jun-2012	Private Collection of Oliver Niehuis	BOLD:ACC4462	675[0n]
**Parnopes grandior**	KY430816.2_tmp	Italy	14-Jun-2011	Private Collection of Oliver Niehuis	BOLD:AAL3875	444[0n]
**Parnopes grandior**	BC ZSM HYM 07316	France	16-Jul-2009	SNSB, Zoologische Staatssammlung Muenchen	BOLD:AAL3875	658[0n]
**Parnopes grandior**	BC ZSM HYM 07317	France	16-Jul-2009	SNSB, Zoologische Staatssammlung Muenchen	BOLD:AAL3875	658[0n]
**Parnopes grandior**	BC ZSM HYM 06313	Germany	03-Jul-2001	SNSB, Zoologische Staatssammlung Muenchen	BOLD:AAL3875	633[0n]
**Parnopes grandior**	BC ZSM HYM 06314	Italy	08-Jun-2007	SNSB, Zoologische Staatssammlung Muenchen	BOLD:AAL3875	658[0n]
**Parnopes grandior**	BC ZSM HYM 06315	Italy	08-Jun-2007	SNSB, Zoologische Staatssammlung Muenchen	BOLD:AAL3875	658[0n]
**Parnopes grandior**	BC ZSM HYM 14953	Germany	05-Jul-2011	SNSB, Zoologische Staatssammlung Muenchen	BOLD:AAL3875	658[0n]
**Parnopes grandior**	BC ZSM HYM 14952	Germany	02-Jul-2012	SNSB, Zoologische Staatssammlung Muenchen	BOLD:AAL3875	634[0n]
**Parnopes grandior**	BC ZSM HYM 14954	Germany	05-Jul-2011	SNSB, Zoologische Staatssammlung Muenchen	BOLD:AAL3875	634[0n]
**Philoctetes bidentulus**	BC ZSM HYM 08030	Germany	30-Jul-2006	SNSB, Zoologische Staatssammlung Muenchen	BOLD:AAY9791	658[0n]
**Philoctetes bidentulus**	BC ZSM HYM 17360	Italy	20-Jul-2012	SNSB, Zoologische Staatssammlung Muenchen	BOLD:AAY9791	618[0n]
**Philoctetes bidentulus**	BC ZSM HYM 07990	Germany	16-Jun-2010	SNSB, Zoologische Staatssammlung Muenchen	BOLD:AAY9791	658[0n]
**Philoctetes bidentulus**	BC ZSM HYM 07991	Germany	10-Jun-2008	SNSB, Zoologische Staatssammlung Muenchen	BOLD:AAY9791	658[0n]
**Philoctetes bidentulus**	BC ZSM HYM 07992	Germany	13-Jul-2008	SNSB, Zoologische Staatssammlung Muenchen	BOLD:AAY9791	658[0n]
**Philoctetes putoni**	BC ZSM HYM 16243	France	13-Jul-2010	SNSB, Zoologische Staatssammlung Muenchen	BOLD:ACE0415	658[0n]
**Philoctetes truncatus**	ON_14388	Germany	27-May-2019	Private Collection of Oliver Niehuis	BOLD:AEJ8804	618[0n]
**Pseudochrysis neglecta**	KY430801.1_tmp	Germany	21-Apr-2011	Private Collection of Oliver Niehuis	BOLD:AAL1591	675[0n]
**Pseudochrysis neglecta**	BC ZSM HYM 07808	Germany	26-Jun-2010	SNSB, Zoologische Staatssammlung Muenchen	BOLD:AAL1591	658[0n]
**Pseudochrysis neglecta**	BC ZSM HYM 06310	Germany	17-Jun-2002	SNSB, Zoologische Staatssammlung Muenchen	BOLD:AAL1591	658[0n]
**Pseudochrysis neglecta**	BC ZSM HYM 06311	Germany	16-Jun-2008	SNSB, Zoologische Staatssammlung Muenchen	BOLD:AAL1591	658[0n]
**Pseudochrysis neglecta**	BC ZSM HYM 06312	Germany	17-Jun-2002	SNSB, Zoologische Staatssammlung Muenchen	BOLD:AAL1591	654[0n]
**Pseudomalus auratus**	KY430823.1_tmp	Germany	05-Jun-2011	Private Collection of Oliver Niehuis	BOLD:AAH8217	675[0n]

**Pseudomalus auratus**	BC ZSM HYM 15248	Germany	04-Aug-2012	SNSB, Zoologische Staatssammlung Muenchen	BOLD:AAH8217	658[0n]
**Pseudomalus auratus**	BC ZSM HYM 00058	Austria	23-Jun-2006	SNSB, Zoologische Staatssammlung Muenchen	BOLD:AAH8217	658[0n]
**Pseudomalus auratus**	BC ZSM HYM 17357	Italy	20-Jul-2012	SNSB, Zoologische Staatssammlung Muenchen	BOLD:AAH8217	658[0n]
**Pseudomalus auratus**	BC ZSM HYM 05073	Germany	02-Jul-2008	SNSB, Zoologische Staatssammlung Muenchen	BOLD:AAH8217	658[0n]
**Pseudomalus auratus**	BC ZSM HYM 05074	Germany	02-Jul-2008	SNSB, Zoologische Staatssammlung Muenchen	BOLD:AAH8217	658[0n]
**Pseudomalus auratus**	BC ZSM HYM 05075	Germany	02-Jul-2008	SNSB, Zoologische Staatssammlung Muenchen	BOLD:AAH8217	658[0n]
**Pseudomalus auratus**	BC ZSM HYM 05076	Austria	02-Jul-2006	SNSB, Zoologische Staatssammlung Muenchen	BOLD:AAH8217	658[0n]
**Pseudomalus auratus**	BC ZSM HYM 14122	Germany	19-Jul-2008	SNSB, Zoologische Staatssammlung Muenchen	BOLD:AAH8217	658[0n]
**Pseudomalus pusillus**	KY430822.1_tmp	Germany	29-Jul-2009	Private Collection of Oliver Niehuis	BOLD:AAL1775	657[0n]
**Pseudomalus pusillus**	BC ZSM HYM 08035	Germany	14-Jun-2010	SNSB, Zoologische Staatssammlung Muenchen	BOLD:AAL1775	658[2n]
**Pseudomalus pusillus**	BC ZSM HYM 08036	Germany	17-Jul-2010	SNSB, Zoologische Staatssammlung Muenchen	BOLD:AAL1775	658[0n]
**Pseudomalus pusillus**	BC ZSM HYM 08037	Germany	27-Jul-2010	SNSB, Zoologische Staatssammlung Muenchen	BOLD:AAL1775	658[0n]
**Pseudomalus pusillus**	BC ZSM HYM 08040	Germany	16-Jul-2010	SNSB, Zoologische Staatssammlung Muenchen	BOLD:AAL1775	658[0n]
**Pseudomalus pusillus**	BC ZSM HYM 07407	Germany	22-Jul-2009	SNSB, Zoologische Staatssammlung Muenchen	BOLD:AAL1775	658[0n]
**Pseudomalus pusillus**	BC ZSM HYM 11083	Germany	16-Jul-2011	SNSB, Zoologische Staatssammlung Muenchen	BOLD:AAL1775	658[0n]
**Pseudomalus pusillus**	BC ZSM HYM 11084	Germany	16-Jul-2011	SNSB, Zoologische Staatssammlung Muenchen	BOLD:AAL1775	658[0n]
**Pseudomalus pusillus**	BC ZSM HYM 17358	Germany	18-Jul-2012	SNSB, Zoologische Staatssammlung Muenchen	BOLD:AAL1775	658[0n]
**Pseudomalus pusillus**	BC ZSM HYM 17359	Germany	18-Jul-2012	SNSB, Zoologische Staatssammlung Muenchen	BOLD:AAL1775	658[0n]
**Pseudomalus pusillus**	BC-ZSM-HYM-23654-F06	Slovakia	06-Aug-2008	SNSB, Zoologische Staatssammlung Muenchen	BOLD:AAL1775	658[0n]
**Pseudomalus pusillus**	BC ZSM HYM 14062	Germany	16-Jul-2011	SNSB, Zoologische Staatssammlung Muenchen	BOLD:AAL1775	658[0n]
**Pseudomalus pusillus**	BC ZSM HYM 14063	Germany	16-Jul-2011	SNSB, Zoologische Staatssammlung Muenchen	BOLD:AAL1775	658[0n]
**Pseudomalus pusillus**	BC ZSM HYM 14064	Germany	16-Jul-2011	SNSB, Zoologische Staatssammlung Muenchen	BOLD:AAL1775	658[0n]
**Pseudomalus pusillus**	BC-ZSM-HYM-23654-F05	Slovakia	06-Aug-2008	SNSB, Zoologische Staatssammlung Muenchen	BOLD:AAL1775	658[0n]

**Pseudomalus pusillus**	BC ZSM HYM 14967	Germany	19-Jun-2012	SNSB, Zoologische Staatssammlung Muenchen	BOLD:AAL1775	658[0n]
**Pseudomalus pusillus**	BC ZSM HYM 14968	Germany	02-Jul-2012	SNSB, Zoologische Staatssammlung Muenchen	BOLD:AAL1775	658[0n]
**Pseudomalus triangulifer**	ON7898	Germany	15-May-2014	Research Collection of Oliver Niehuis		623[21n]
**Pseudomalus triangulifer**	ON7196	Germany	13-Jun-2014	Research Collection of Oliver Niehuis		652[11n]
**Pseudomalus triangulifer**	ON4528	Germany	15-May-2012	Research Collection of Oliver Niehuis		637[10n]
**Pseudomalus triangulifer**	ON11505	Germany	15-May-2017	Research Collection of Oliver Niehuis	BOLD:AEC6303	676[1n]
**Pseudomalus triangulifer**	ON6708	Germany	15-May-2013	Research Collection of Oliver Niehuis	BOLD:AEC6303	698[1n]
**Pseudomalus violaceus**	BC ZSM HYM 12107	Germany	15-Aug-2007	SNSB, Zoologische Staatssammlung Muenchen	BOLD:ABX9998	553[2n]
**Spinolia unicolor**	BC ZSM HYM 06308	Germany	03-Jul-2008	SNSB, Zoologische Staatssammlung Muenchen	BOLD:AAP1301	658[0n]
**Spinolia unicolor**	BC ZSM HYM 06309	Germany	03-Jul-2008	SNSB, Zoologische Staatssammlung Muenchen	BOLD:AAP1301	658[0n]
**Spintharina versicolor**	KY430789.1_tmp	Italy	10-May-2011	Private Collection of Oliver Niehuis	BOLD:AAJ3630	675[0n]
**Spintharina versicolor**	BC ZSM HYM 07342	Italy	17-Jun-2009	SNSB, Zoologische Staatssammlung Muenchen	BOLD:AAJ3630	658[0n]
**Spintharina versicolor**	BC ZSM HYM 07343	Italy	17-Jun-2009	SNSB, Zoologische Staatssammlung Muenchen	BOLD:AAJ3630	658[0n]
**Spintharina versicolor**	BC ZSM HYM 07813	France	15-Jul-2010	SNSB, Zoologische Staatssammlung Muenchen	BOLD:AAJ3630	658[0n]
**Spintharina versicolor**	BC ZSM HYM 07814	France	15-Jul-2010	SNSB, Zoologische Staatssammlung Muenchen	BOLD:AAJ3630	658[0n]
**Stilbum calens**	BC ZSM HYM 17355	Italy	20-Jul-2012	SNSB, Zoologische Staatssammlung Muenchen	BOLD:AAJ4206	658[0n]
**Stilbum calens**	BC ZSM HYM 17356	Italy	20-Jul-2012	SNSB, Zoologische Staatssammlung Muenchen	BOLD:AAJ4206	658[0n]
**Trichrysis cyanea**	KY430758.1_tmp	Germany	30-May-2009	Private Collection of Oliver Niehuis	BOLD:AAH7935	657[0n]
**Trichrysis cyanea**	BC ZSM HYM 14693	Germany	21-May-2012	SNSB, Zoologische Staatssammlung Muenchen	BOLD:AAH7935	658[0n]
**Trichrysis cyanea**	BC ZSM HYM 14694	Germany	21-May-2012	SNSB, Zoologische Staatssammlung Muenchen	BOLD:AAH7935	621[0n]
**Trichrysis cyanea**	BC ZSM HYM 14695	Germany	21-May-2012	SNSB, Zoologische Staatssammlung Muenchen	BOLD:AAH7935	658[0n]
**Trichrysis cyanea**	BC ZSM HYM 14696	Germany	21-May-2012	SNSB, Zoologische Staatssammlung Muenchen	BOLD:AAH7935	658[0n]
**Trichrysis cyanea**	BC ZSM HYM 05058	Germany	14-Aug-2008	SNSB, Zoologische Staatssammlung Muenchen	BOLD:AAH7935	658[0n]
**Trichrysis cyanea**	BC ZSM HYM 00060	Germany	20-May-2007	SNSB, Zoologische Staatssammlung Muenchen	BOLD:AAH7935	658[0n]

**Trichrysis cyanea**	BC ZSM HYM 17343	Germany	18-Jul-2012	SNSB, Zoologische Staatssammlung Muenchen	BOLD:AAH7935	658[0n]
**Trichrysis cyanea**	BC ZSM HYM 17344	Germany	18-Jul-2012	SNSB, Zoologische Staatssammlung Muenchen	BOLD:AAH7935	658[0n]
**Trichrysis cyanea**	BC ZSM HYM 17345	Germany	24-May-2008	SNSB, Zoologische Staatssammlung Muenchen	BOLD:AAH7935	658[0n]
**Trichrysis cyanea**	BC ZSM HYM 05057	Germany	10-Jun-2006	SNSB, Zoologische Staatssammlung Muenchen	BOLD:AAH7935	658[0n]
**Trichrysis cyanea**	BC ZSM HYM 05059	Germany	03-Jul-2008	SNSB, Zoologische Staatssammlung Muenchen	BOLD:AAH7935	658[0n]
**Trichrysis cyanea**	BC ZSM HYM 05060	Germany	19-Jul-2008	SNSB, Zoologische Staatssammlung Muenchen	BOLD:AAH7935	658[0n]
**Trichrysis cyanea**	BC ZSM HYM 14065	Germany	16-Jul-2011	SNSB, Zoologische Staatssammlung Muenchen	BOLD:AAH7935	658[0n]
**Trichrysis cyanea**	BC ZSM HYM 14066	Germany	16-Jul-2011	SNSB, Zoologische Staatssammlung Muenchen	BOLD:AAH7935	658[0n]
