# Supplementary material for: DNA Barcoding of German Cuckoo Wasps (Hymenoptera: Chrysididae) Suggests Cryptic Species in Several Widely Distributed Species
Source: Insects. 2024 Oct 30;15(11):850. doi: 10.3390/insects15110850 (PMC11594385; doi:10.3390/insects15110850)
Supplement: Supplementary file 1 [file insects-15-00850-s001.zip › Supplementary materials/Supplement 3 Chrysididae statistics.pdf]

Supplement 3: Barcoding statistics of with mean intraspecific distance, maximum intraspecific distance, nearest neighbour species, distance to nearest neighbour species, Barcode Index Number (BIN), country, and number of specimens. Asterisks indicate species with BIN sharing.

Subfamily	Species	Mean	Max	Nearest species	NN distance	BIN	Country	Specimens
**Chrysidinae**	Chrysellampus sculpticollis	N/A	0.00	Elampus panzeri	11.77	BOLD:AEJ0421	Italy	1
	Chrysidea disclusa	N/A	0.00	Chrysura hirsuta	9.49	BOLD:AEC9712	Italy	1
	Chrysis analis	0.10	0.31	Chrysis marginata	10.20	BOLD:AAJ4964	Germany	9
							Italy	1
	Chrysis angustula	0.72	1.70	Chrysis longula	6.89	BOLD:AAV7326	Germany	17
							Italy	1
	Chrysis bicolor	1.65	3.39	Chrysis germari	7.90	BOLD:AAY6926	France	2
						BOLD:AAY6947	Germany	6
						BOLD:AEC7638	Italy	1
	Chrysis brevitarsis	0.13	0.13	Chrysis parabrevitarsis	2.50	BOLD:ACG7211	Sweden	1
							Lithuania	1
	Chrysis clarinicollis	0.00	0.00	Chrysis longula	7.56	BOLD:ACQ7862	Germany	3
	Chrysis comparata	0.00	0.00	Chrysura hirsuta	13.31	BOLD:AAU1528	France	1
							Italy	1
	Chrysis consanguinea	N/A	0.00	Chrysis viridula	7.68	BOLD:AED0671	Spain	1
	Chrysis cortii	1.00	1.88	Chrysis leachii	11.90	BOLD:AAR9816	France	2
							Germany	3
							Portugal	1
	Chrysis corusca	0.08	0.48	Chrysis vanlithi	8.23	BOLD:ACF7605	Germany	52

							Sweden	1
	Chrysis equestris	0.31	0.31	Chrysis fasciata	7.75	BOLD:AAP1365	Germany	2
							Italy	1
	Chrysis fasciata	0.36	0.61	Chrysis equestris	7.75	BOLD:AAR9820	Germany	7
							Italy	1
	Chrysis fulgida	0.18	0.46	Chrysis vanlithi	9.09	BOLD:AAP1068	Germany	7
	Chrysis germari	0.13	0.31	Chrysis bicolor	7.90	BOLD:AAJ4844	Italy	4
	Chrysis gracillima	0.06	0.15	Chrysura hirsuta	8.10	BOLD:AAJ4865	Germany	2
							Italy	3
	Chrysis horridula	0.43	1.37	Chrysis ignita	5.95	BOLD:AAU2328	France	1
							Germany	11
	Chrysis ignita	0.00	0.00	Chrysis impressa	0.60	BOLD:AAG0244*	Germany	11
	Chrysis illigeri	0.61	1.56	Chrysis bicolor	11.08	BOLD:AAV9309	Germany	13
							Italy	3
	Chrysis immaculata	N/A	0.00	Chrysis corusca	8.65	BOLD:AEC7328	Germany	1
	Chrysis impressa	0.15	0.48	Chrysis ignita	0.60	BOLD:AAG0244*	Germany	6
							Netherlands	2
							Sweden	1
	Chrysis inaequalis	0.14	0.31	Chrysis marginata	12.51	BOLD:AAY6948	Germany	6
	Chrysis indigotea	0.11	0.17	Chrysis impressa	10.32	BOLD:AAP1364	Germany	3
	Chrysis iris	0.51	0.77	Chrysis fulgida	9.63	BOLD:ABU6374	Germany	1
							No BIN	2
	Chrysis lanceolata	N/A	0.00	Chrysis germari	11.92	BOLD:AAY6950	Germany	1

	Chrysis leachii	0.21	0.32	Chrysis cortii	11.90	BOLD:AAY6925	France	3
	Chrysis leptomandibularis	0.00	0.00	Chrysis impressa	2.26	BOLD:ACQ4597	Germany	1
							Italy	1
	Chrysis longula	0.20	1.13	Chrysis subcoriacea	6.71	BOLD:ACF9032	Germany	25
	Chrysis marginata	0.46	0.46	Chrysura hirsuta	9.18	BOLD:ACG4638	Germany	1
							Greece	1
	Chrysis mediata	0.26	1.29	Chrysis solida	0.15	BOLD:AAY6949*	Germany	18
							Italy	1
	Chrysis parabrevitarsis	0.23	1.96	Chrysis brevitarsis	2.50	BOLD:ACF7346	Germany	1
						BOLD:ACG6749	Germany	26
	Chrysis parietis	0.50	1.50	Chrysis schencki	1.80	BOLD:AAU2329	Germany	11
							Hungary	1
						No BIN	Germany	1
	Chrysis pseudobrevitarsis	0.38	0.60	Chrysis parabrevitarsis	3.81	BOLD:ACG6983	Germany	4
	Chrysis pulchella	N/A	0.00	Chrysura hirsuta	10.36	BOLD:AED0619	Spain	1
	Chrysis ruddii	0.44	0.93	Chrysis rutiliventris	9.18	BOLD:AAY6944	France	1
							Germany	6
	Chrysis rutilans	0.00	0.00	Chrysis splendidula	8.06	BOLD:ABA9738	Germany	5
	Chrysis rutiliventris	0.31	0.62	Chrysis ruddii	9.18	BOLD:AAY6931	France	2
							Italy	2
	Chrysis schencki	0.65	2.41	Chrysis parietis	1.80	BOLD:ABU6375	Germany	9
						BOLD:ACF6219	Germany	35

							Sweden	1
	Chrysis sculpturata	N/A	0.00	Chrysis ignita	6.92	BOLD:ABU6373	France	1
	Chrysis scutellaris	0.59	1.24	Chrysis horridula	9.29	BOLD:AAR9833	Germany	7
							Italy	5
						No BIN	Italy	2
	Chrysis solida	0.22	0.66	Chrysis mediata	0.15	BOLD:AAY6949*	Germany	19
							Italy	6
							Sweden	1
	Chrysis splendidula	0.18	0.31	Chrysis rutilans	8.06	BOLD:AAR9835	Czech Republic	1
							France	1
							Germany	2
							Italy	1
	Chrysis subcoriacea	0.00	0.00	Chrysis vanlithi	6.15	BOLD:ACF9031	Germany	2
							Italy	1
	Chrysis succincta	0.06	0.15	Chrysis bicolor	10.72	BOLD:AEC9270	France	1
							Germany	4
	Chrysis sybarita	0.16	0.47	Chrysura hirsuta	11.40	BOLD:AAJ4866	Germany	6
	Chrysis terminata	0.38	1.44	Chrysis impressa	1.96	BOLD:ABY5626	France	1
							Germany	25
							Italy	1
							Sweden	1
	Chrysis vanlithi	N/A	0.00	Chrysis subcoriacea	6.15	BOLD:ACJ4881	Switzerland	1
	Chrysis viridula	0.06	0.15	Chrysis consanguinea	7.68	BOLD:AAJ0207	France	1
							Germany	4
	Chrysura austriaca	0.00	0.00	Chrysura hirsuta	9.84	BOLD:AAJ3472	Germany	12

	Chrysura cuprea	0.19	0.49	Chrysura hirsuta	6.75	BOLD:AAP1055	Italy	6
	Chrysura dichroa	1.24	2.56	Chrysura laevigata	5.26	BOLD:AAJ3474	Italy	1
						BOLD:AFA5928	Hungary	2
							Italy	3
	Chrysura hirsuta	0.58	0.93	Chrysura laevigata	6.08	BOLD:AAY6945	France	3
							Germany	1
	Chrysura hybrida	0.00	0.00	Chrysura hirsuta	9.19	BOLD:AAY6924	France	2
	Chrysura laevigata	N/A	0.00	Chrysura dichroa	5.26	BOLD:ACG1433	Italy	1
	Chrysura radians	0.00	0.00	Chrysura hirsuta	7.76	BOLD:ABX8519	Germany	2
	Chrysura rufiventris	N/A	0.00	Chrysura cuprea	8.26	BOLD:AEC6882	Germany	1
	Chrysura simplex	0.29	0.50	Chrysura dichroa	10.02	BOLD:AAY6923	France	3
							Italy	2
	Chrysura trimaculata	0.00	0.00	Chrysura laevigata	8.67	BOLD:AAR9311	Germany	8
	Elampus bidens	N/A	0.00	Elampus panzeri	10.89	BOLD:AEC6927	Italy	1
	Elampus constrictus	N/A	0.00	Elampus konowi	9.16	BOLD:ACC2184	Czech Republic	1
	Elampus konowi	0.09	0.16	Elampus constrictus	9.16	BOLD:AAY9882	Germany	5
	Elampus panzeri	N/A	0.00	Elampus constrictus	9.46	BOLD:AEA0923	Germany	1
	Euchroeus purpuratus	N/A	0.00	Chrysis gracillima	12.68	BOLD:AED0036	Morocco	1
	Hedychridium aereolum	0.82	1.30	Hedychridium ardens	11.75	BOLD:AAY6930	France	3
							Italy	1
	Hedychridium ardens	0.22	1.09	Hedychridium aereolum	11.75	BOLD:AAK4640	Germany	19

	Hedychridium caputaureum	0.44	0.67	Hedychridium roseum	7.20	BOLD:AAU0775	Germany	3
	Hedychridium coriaceum	0.70	2.35	Hedychridium krajniki	10.88	BOLD:AAY6943	Germany	9
	Hedychridium cupratum	0.42	0.64	Hedychridium coriaceum	14.93	BOLD:AAY6946	France	2
							Italy	1
	Hedychridium cupreum	0.09	0.16	Hedychridium purpurascens	11.30	BOLD:AAY9838	Germany	5
	Hedychridium elegantulum	0.00	0.00	Hedychridium femoratum	11.94	BOLD:AAY9837	Germany	5
	Hedychridium femoratum	N/A	0.00	Hedychridium elegantulum	11.94	BOLD:AAV6886	Slovakia	1
	Hedychridium jucundum	0.77	0.77	Hedychridium ardens	12.64	BOLD:AAU1479	Czech Republic	1
							Slovakia	1
	Hedychridium krajniki	0.25	0.61	Hedychridium coriaceum	10.88	BOLD:AAZ0056	Czech Republic	1
							Germany	4
	Hedychridium monochroum	N/A	0.00	Hedychridium roseum	16.51	BOLD:AAY1978	Italy	1
	Hedychridium niemelai	0.00	0.00	Hedychrum niemelai	0.00	BOLD:AAU1294*	Germany	2
	Hedychridium purpurascens	N/A	0.00	Hedychridium cupreum	11.30	BOLD:AEP5116	Germany	1
	Hedychridium roseum	1.50	5.85	Hedychridium rossicum	7.08	BOLD:AAE3259	Germany	6
							Italy	2
						BOLD:AAE3260	Italy	1
						BOLD:ACG6690	Germany	4
	Hedychridium rossicum	0.61	1.15	Hedychridium roseum	7.08	BOLD:AAE3258	Germany	9
	Hedychridium zelleri	0.00	0.00	Hedychridium jucundum	16.27	BOLD:ACG7149	Germany	3
	Hedychrum chalybaeum	0.19	0.46	Hedychrum niemelai	10.61	BOLD:AAY6942	Germany	10

	Hedychrum gerstaeckeri	0.04	0.31	Hedychrum longicolle	9.32	BOLD:AAM3758	Germany	17
	Hedychrum longicolle	N/A	0.00	Hedychrum gerstaeckeri	9.32	BOLD:AED0972	France	1
	Hedychrum niemelai	0.23	0.77	Hedychridium niemelai	0.00	BOLD:AAU1294*	Germany	22
							Italy	1
						No BIN	Germany	1
	Hedychrum nobile	0.41	1.24	Hedychrum niemelai	9.03	BOLD:AAK4644	Germany	12
							Italy	1
	Hedychrum rutilans	0.63	1.55	Hedychrum viridiaureum	4.78	BOLD:AAK4643	Germany	8
							Italy	2
	Hedychrum viridiaureum	0.09	0.31	Hedychrum rutilans	4.78	BOLD:AAM3491	Germany	7
	Holopyga austrialis	0.06	0.15	Holopyga chrysonota	9.49	BOLD:ABX5007	Germany	5
	Holopyga chrysonota	1.43	5.11	Holopyga similis	9.32	BOLD:AAV7063	Germany	1
						BOLD:AAY9689	Germany	6
							Italy	1
	Holopyga fervida	4.59	7.28	Holopyga generosa	12.10	BOLD:AAX1104	Germany	1
						BOLD:AAY9735	Germany	1
							Spain	3
						BOLD:ACV6331	Germany	2
	Holopyga generosa	6.72	13.42	Holopyga chrysonota	9.80	BOLD:AAY6927	France	2
						BOLD:AAY6928	France	2
							Germany	3
						BOLD:AAZ6194	Germany	1

						BOLD:ACC3318	Czech Republic	1
							Germany	6
							Italy	1
	Holopyga minuma	N/A	0.00	Holopyga generosa	11.02	BOLD:AAV7065	Slovakia	1
	Holopyga similis	N/A	0.00	Holopyga chrysonota	9.32	BOLD:AED0274	Germany	1
	Omalus aeneus	7.94	9.13	Omalus puncticollis	0.15	BOLD:ACC4462*	Italy	1
						BOLD:ACG9650	Slovakia	1
						BOLD:ACQ9469	Hungary	1
	Omalus biaccinctus	0.62	0.62	Omalus aeneus	8.93	BOLD:AFA2340	Germany	2
	Omalus puncticollis	N/A	0.00	Omalus aeneus	0.15	BOLD:ACC4462*	Germany	1
	Parnopes grandior	1.29	2.34	Chrysis rutilans	19.74	BOLD:AAL3875	France	2
							Germany	4
							Italy	3
	Philoctetes bidentulus	0.68	1.48	Pseudomalus auratus	11.20	BOLD:AAY9791	Germany	4
							Italy	1
	Philoctetes putoni	N/A	0.00	Philoctetes bidentulus	11.43	BOLD:ACE0415	France	1
	Philoctetes truncatus	N/A	0.00	Elampus constrictus	11.81	BOLD:AEJ8804	Germany	1
	Pseudochrysis neglecta	1.07	2.68	Chrysura dichroa	15.77	BOLD:AAL1591	Germany	5
	Pseudomalus auratus	0.73	2.02	Pseudomalus pusillus	7.90	BOLD:AAH8217	Austria	2
							Germany	6
							Italy	1
	Pseudomalus pusillus	0.22	0.62	Pseudomalus auratus	7.90	BOLD:AAL1775	Germany	15
							Slovakia	2

	Pseudomalus triangulifer	0.10	0.17	Pseudomalus auratus	9.43	BOLD:AEC6303	Germany	2
						No BIN	Germany	3
	Pseudomalus violaceus	N/A	0.00	Pseudomalus triangulifer	12.74	BOLD:ABX9998	Germany	1
	Spinolia unicolor	0.61	0.61	Chrysis gracillima	13.12	BOLD:AAP1301	Germany	2
	Spintharina versicolor	0.34	0.61	Chrysura hirsuta	10.54	BOLD:AAJ3630	France	2
							Italy	3
	Stilbum calens	0.00	0.00	Chrysis ignita	18.95	BOLD:AAJ4206	Italy	2
	Trichrysis cyanea	0.02	0.16	Chrysis marginata	12.51	BOLD:AAH7935	Germany	15
**Cleptinae**	Cleptes nitidulus	0.09	0.31	Cleptes semiauratus	17.11	BOLD:AAZ1160	Germany	6
	Cleptes semiauratus	1.73	3.14	Cleptes striatipleuris	10.54	BOLD:AAJ3895	Germany	3
						BOLD:AAM4041	Germany	4
	Cleptes splendidus	0.31	0.31	Cleptes semiauratus	17.15	BOLD:AAR9529	Germany	2
	Cleptes striatipleuris	N/A	0.00	Cleptes semiauratus	10.54	BOLD:AAU2238	Germany	1
